# Identification of potent chromone embedded [1,2,3]-triazoles as novel anti-tubercular agents

**DOI:** 10.1098/rsos.171750

**Published:** 2018-04-04

**Authors:** Viswanadh Nalla, Aslam Shaikh, Sanket Bapat, Renu Vyas, M. Karthikeyan, P. Yogeeswari, D. Sriram, M. Muthukrishnan

**Affiliations:** 1 CSIR-National Chemical Laboratory, Dr Homi Bhabha Road, Pune 411008, India; 2 Academy of Scientific and Innovative Research (AcSIR), New Delhi 110 025, India; 3 MIT School of Bioengineering Sciences and Research, MIT Art, Design and Technology University, Pune 412 201, India; 4 Tuberculosis Drug Discovery Laboratory, Pharmacy Group, Birla Institute of Technology and Science-Pilani, Hyderabad Campus, Hyderabad 500 0078, India

**Keywords:** chromone, triazole, molecular docking, anti-mycobacterial activity

## Abstract

A series of 20 novel chromone embedded [1,2,3]-triazoles derivatives were synthesized via an easy and convenient synthetic procedure starting from 2-hydroxy acetophenone. The *in vitro* anti-mycobacterial evaluation studies carried out in this work reveal that seven compounds exhibit significant inhibition against *Mycobacterium tuberculosis* H37Rv strain with MIC in the range of 1.56–12.5 µg ml^−1^. Noticeably, compound **6s** was the most potent compound *in vitro* with a MIC value of 1.56 µg ml^−1^. Molecular docking and chemoinformatics studies revealed that compound **6s** displayed drug-like properties against the enoyl-acyl carrier protein reductase of *M. tuberculosis* further establishing its potential as a potent inhibitor.

## 1. Introduction

Tuberculosis is a major infectious disease caused by *Mycobacterium tuberculosis* (*Mtb*) and it is estimated that there were 10.4 million new cases and 1.4 million deaths in 2015 alone, of which developing countries showed a major share [1]. Recent study reveals that the numbers of TB cases in India are two to three times higher than previously estimated suggesting that the global number of TB cases might be largely underestimated [2]. Furthermore, the emergence of a drug-resistant microorganism responsible for TB, especially multidrug-resistant one, along with lethal combination of TB and HIV infection makes this disease even more challenging [3–5]. In the last 50 years, only a few drugs have been approved by the FDA to treat TB. Therefore, the discovery and development of novel anti-TB agents with new chemotypes acting on novel drug targets is an important task for infectious diseases research programmes.

**Figure 1.**
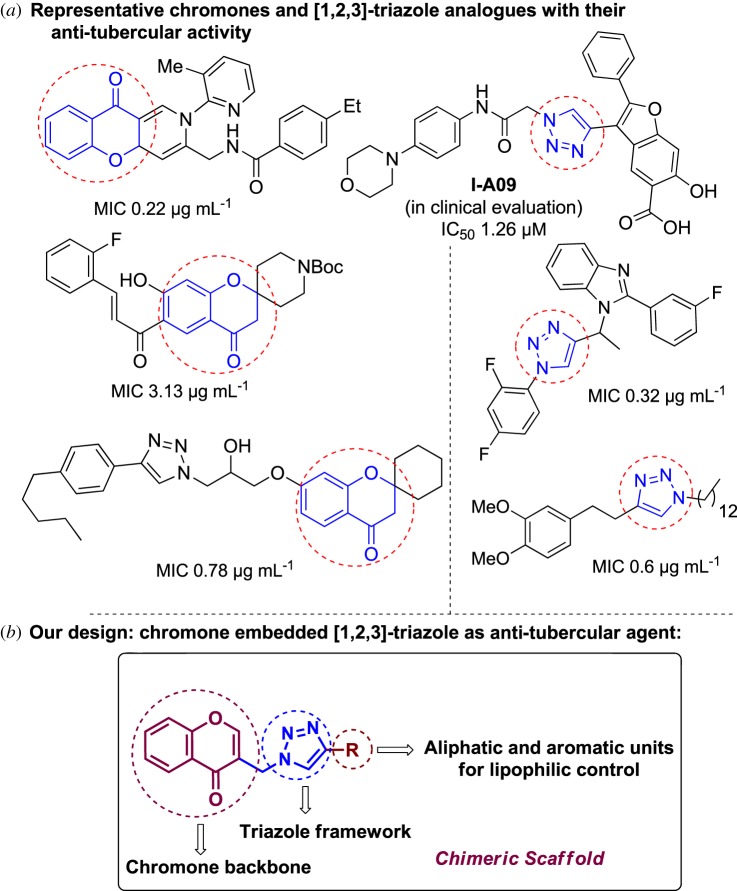
(*a*) Representative example of chromones and [1,2,3]-triazole analogues and their anti-tubercular activity; (*b*) our design of chromone embedded [1,2,3]-triazole framework as chimeric scaffold.

Natural products have a rich history as lead compounds for drug discovery. Further, natural products have contributed significantly to the current portfolio of anti-TB drugs, with one first-line drug (rifampicin) and several second-line agents (kanamycin, viomycin, etc.) being either natural products themselves or being derived from a natural product lead [6,7]. Chromone frameworks are frequently found in a diverse array of natural products, that includes natural flavone/isoflavone products, therapeutically active drugs such as anti-inflammatory, anti-platelet, anti-microbial, anti-obesity, anti-cancer agents, drug candidates for neurodegenerative diseases and adenosine receptors [8–10]. In fact, biological activities of these chromone molecules mainly depend on the conjugated bi- and tricyclic motifs with ketone functionality, but vary depending on the nature, position and variation of substituents. In this context, and in view of our continuing interest in the chemistry of privileged chromone motif [11–13], in particular, the design and synthesis of natural products like small molecules based on the chromone motif for various biological applications, herein, we designed and synthesized a series of novel chromone embedded 1,4 disubstituted [1,2,3]-triazole analogues using chimeric approach [14–16] and evaluated their anti-mycobacterial potential against *M. tuberculosis* H37Rv (figure 1). The interest in incorporation of [1,2,3]-triazole moiety stems from the advent of click chemistry protocol [17,18], which has been used in various applications including drug discovery process. In addition, triazole embedded heterocyclic frameworks exhibit plethora of biological activities, especially anti-mycobacterial activity (figure 1) [19–22]. Furthermore, these triazole products are considered as *aggressive pharmacophores* that can actively engage in drug–receptor interactions while maintaining an excellent chemical and metabolic profile [23].

## 2. Results and discussion

### 2.1. Synthesis

**Scheme 1.**
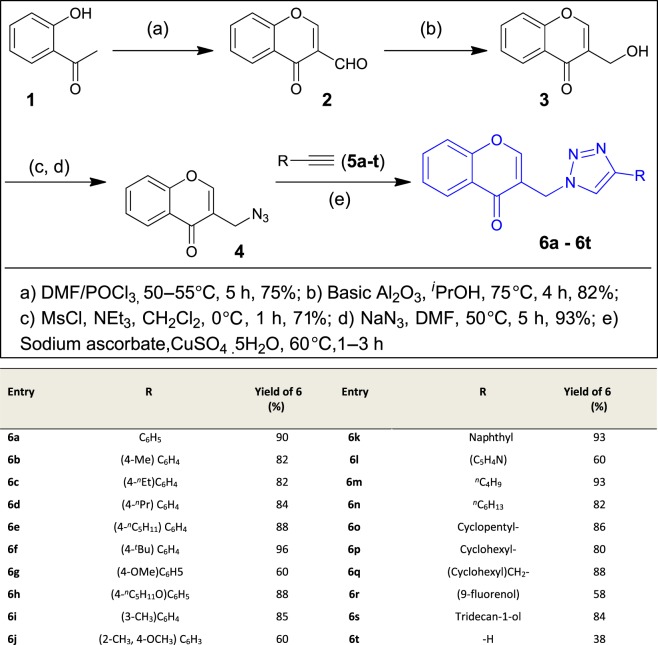
Synthesis of chromone embedded [1,2,3]-triazoles.

A four-step synthetic strategy was followed for the preparation of novel chromone embedded [1,2,3]-triazoles **6a–t** as outlined in scheme 1. At first, 3-formyl chromone **2** was synthesized by formylation of *o*-hydroxylacetophenone **1** using Vilsmeier-Haack reagent (POCl_3_ in DMF) at 55°C for 5 h in 75% yield. Further, 3-formyl chromone **2** was treated for reduction with solid supported basic alumina in isopropanol at 75°C for 4 h to yield 3-hydroxyl methyl chromone **3** in 82% which was subsequently mesylated followed by azidation with sodium azide in DMF at 50°C for 5 h affording the key chromone embedded azide intermediate **4** in 93% yield. Finally, the [1,2,3]-triazole core was incorporated through copper catalysed 1,3 dipolar cycloaddition of 2-azido methyl chromone (**4**) with commercially available different alkyl/aryl terminal alkynes (**5a–t**) in the presence of sodium ascorbate in *t*-BuOH/H_2_O (1 : 1, v/v) solvent mixture. This resulted in the formation of chromone embedded triazole compounds (**6a–t**), respectively, in good to excellent yields (scheme 1). The structures of all the newly synthesized compounds **6a–t** were confirmed by the ^1^H NMR, ^13^C NMR and mass spectral data (electronic supplementary material). In the ^1^H NMR spectra of compound **6a** (representative example), a signal corresponding to the CH_2_ protons that bridge the chromone with triazole moiety was observed at *δ* 5.48 ppm (as a singlet). The corresponding ^13^C resonance signal was delineated at *δ* 45.5 ppm and the chromone carbonyl was discernible at *δ* 176.7 ppm. In addition, the appearance of a sharp singlet for 1 proton observed at *δ* 8.22 ppm in the PMR, suggested the presence of 1,2,3 triazole C–H. The appearance of a sharp singlet (1H) observed at *δ* 8.15 ppm in the PMR, suggested the presence olefinic C–H of chromone moiety. The HRMS (ESI) for **6a** shows the *m/z* at 304.1086 for C_18_H_13_O_2_N_3_ [M + H]^+^.

### 2.2. Anti-mycobacterial evaluation

All the new chromone embedded [1,2,3]-triazole derivatives (**6a–t**) were screened for their *in vitro* anti-tubercular activity against *Mycobacterium tuberculosis* H37Rv (ATCC27294) using MABA assay method (see the electronic supplementary material for detailed experimental procedure). The minimum inhibitory concentration (MIC; µg ml^−1^) was determined for each compound. The MIC is defined as the lowest concentration at which complete inhibition of bacterial growth was observed. Ethambutol and rifampicin were used as reference compounds. The MIC values of the synthesized compounds along with the standard drugs for comparison are reported in table 1.

**Table 1.** *In vitro* anti-tubercular activity of chromone embedded [1,2,3]-triazoles against *Mycobacterium tuberculosis* H37Rv.

		MIC				MIC	
entry	compound	(μg ml^−1^)^a^	cytotoxicity*^b^*	entry	compound	(μg ml^−1^)^a^	cytotoxicity*^b^*
**6a**	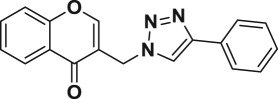	25	12.86	**6k**	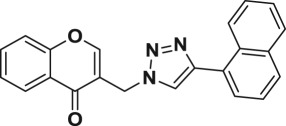	50	26.12
**6b**	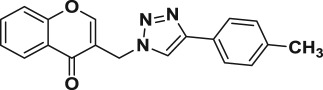	25	26.82	**6l**	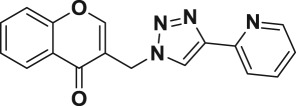	50	30.60
**6c**	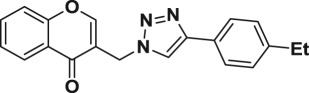	25	16.12	**6m**	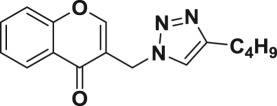	6.25	36.82
**6d**	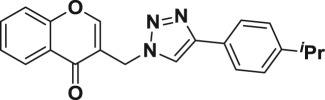	50	20.60	**6n**	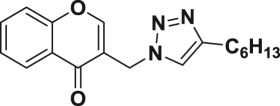	50	23.74
**6e**	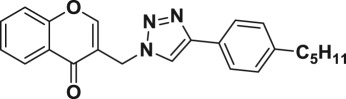	25	20.12	**6o**	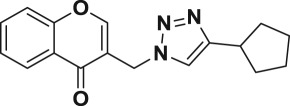	3.125	18.42
**6f**	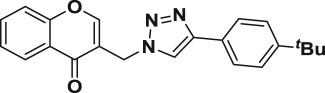	3.125	20.12	**6p**	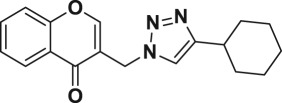	12.5	20.12
**6g**	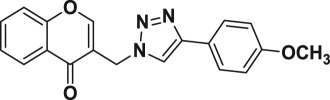	6.25	28.40	**6q**	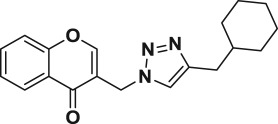	25	28.62
**6h**	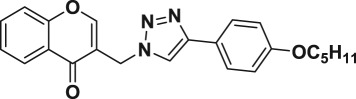	3.125	18.68	**6r**	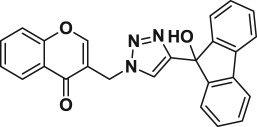	50	30.60
**6i**	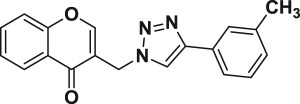	25	26.82	**6s**	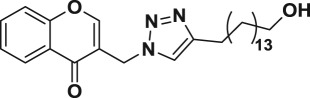	1.56	24.68
**6j**	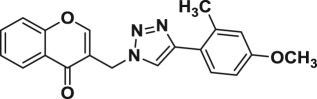	50	30.34	**6t**	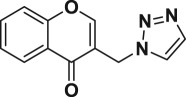	50	30.12

^a^Rifampicin (MIC, 0.24 µg ml^−1^); ethambutol (MIC, 7.64 µg ml^−1^).

^b^Cytotoxicity at 50 µg ml^−1^ (RAW 264.7 cells).

Among the 20 chromone embedded [1,2,3]-triazole derivatives tested, seven compounds (**6f**–**6h**, **6m**, **6o**, **6p** and **6s**) were found to be active with MIC values in the range of 1.56–12.5 µg ml^−1^. The compound **6s** was found to be highly active among all the compounds tested with a MIC value of 1.56 µg ml^−1^, which is 4.8 times more active than the standard drug ethambutol (MIC, 7.64 µg ml^−1^). The preliminary SAR of the chromone embedded triazole analogues reveals that the compounds bearing phenyl group (**6a**) as well as substituted phenyl such as 4-methyl, 4-ethyl, 4-propyl and 4-pentyl (**6b**, **6c**, **6d** and **6e**) do not favour better activity, with the exception of **6f** possessing 4-*t-*butyl group (MIC, 3.125 µg ml^−1^). It was also observed that alkoxy substitution at 4-position of the phenyl ring (**6g** and **6h**) enhances the activity against *Mtb*. However, addition of another methyl group at 2-position of **6g** leads to complete loss of activity, **6i** (MIC, 50 µg ml^−1^). Further, replacement of phenyl group with naphthyl (**6k**) and pyridyl (**6l**) does not appear to enhance the activity (MIC, greater than 50 µg ml^−1^).

Interestingly, modification of the triazole core by changing R group from aromatic to aliphatic group (cyclic or acyclic) enhances the activity against *Mtb*. For example, the compound **6o** possessing cyclopentyl substituent at R position and compound **6m** possessing *n*-butyl substituent exhibit better activity (MIC, 3.125 and 6.25 µg ml^−1^, respectively), with an exception of **6n** possessing *n*-hexyl group (MIC, greater than 50 µg ml^−1^). Importantly, the most active compound in the series, **6s** possess long aliphatic chain terminated with hydrophilic –OH as a capping group (MIC, 1.56 µg ml^−1^).

All chromone embedded [1,2,3]-triazole analogues were also tested for *in vitro* cytotoxicity against RAW 264.7 cells at 50 µg concentration using (4,5-dimethylthiazol-2-yl)-2,5-diphenyl-tetrazoliumbromide (MTT) assay. All the analogues showed less than 50% inhibition, percentage inhibitions of cells are represented in table 1. The most promising anti-tubercular analogues **6f**, **6g**, **6h**, **6m**, **6o** and **6s** exhibited 20.12%, 28.40%, 18.68%, 36.82%, 18.42% and 24.68% growth inhibition, respectively, at 50 µg ml^−1^. The results indicated that potent analogues **6f**, **6h**, **6o** and **6s** are comparatively less toxic and are suitable for further studies.

## 3. Computational studies

*Mycobacterium tuberculosis* inhibitors perform inhibitory action via different mechanistic pathways in the cell. We selected six validated protein targets from each pathway based on their role and importance (table 2) [24]. The biological significance of the selected proteins is discussed in detail herein. Thymidylate kinase (PDB ID: 1G3U) plays a role in the catalysis of the transfer of the phosphoryl moiety from the phosphoryl donor, ATP to TMP which is key intermediate for the DNA-blocking builds [25]. Lumazine synthase (PDB ID: 1W19) catalyses certain steps in riboflavin biosynthesis [26].

**Table 2.** List of tuberculosis targets and mechanistic pathway class.

PDB ID	name of targets	class
1G3U	thymidylate kinase	DNA synthesis
1W19	6,7-dimethyl-8-ribityllumazine synthase	cofactor biosynthesis
1ZID	enoyl-acyl carrier protein	mycolic acid biosynthesis
2OZ5	MTB phosphotyrosine phosphatase B	arrest of phagosome maturation
3IUB	pantothenate synthetase	β-alanine metabolism
1DG5	dihydrofolate reductase	folate metabolism

Enoyl-acyl carrier protein (PDB ID: 1ZID) is essential for fatty acid synthase system (FAS-II) pathway in mycobacterial cells [27], whereas pantothenate synthase (PDB ID: 3IUB) catalyses the condensation of pantonate with β-alanine to form pantothenate, a precursor coenzyme A biosynthesis [28]. MTB phosphotyosine B [MtbPtpB] (PDB ID: 2OZ5) blocks the signal regulated kinase and p-38 mediated by IL-6 thereby promoting mycobacterial survival in the host [29]. Dihydrofolate reductase (PDB ID: 1DG5) helps in regulating the amount of tetrahydrofolate in the cell. Tetrahydrofolate derivatives are key components in purine and thymidylate synthesis, which is important for cell proliferation and cell growth [30].

## 4. Methodology

### 4.1. Preparation of ligands

The two-dimensional structures (.mol) of four compounds, i.e. **6f**, **6h**, **6o** and **6s**, were drawn and the structure was analysed by using Marvin view. The compounds were converted to three-dimensional structure (.pdb) using LigPrep tool [31]. LigPrep is a Schrödinger suite tool which is used to generate three-dimensional structures from two-dimensional structures, search tautomers, isomers for compounds and carry out energy minimization by applying the OPLS 2005 force field.

### 4.2. Preparation of macromolecule

The protein targets retrieved from RCSB Protein Data Bank are proteins associated with metabolic functioning and proliferation of *M. tuberculosis*. The proteins listed in table 2 served as docking receptors. The proteins were fixed for errors in atomic representations and optimized using Protein Preparation Wizard Maestro v. 10.3 (Maestro, v. 10.3: Schrödinger, LLC, New York, NY, USA). The bond orders were assigned to residues, hydrogen atoms were added at pH 7.0. Minimization was carried out using OPLS 2005 force field with a RMSD cut-off value of 0.3 Ǻ.

### 4.3. Molecular docking

The molecular docking was performed and analysed via the Glide v. 6.8 docking tool [32]. The receptor grid was centred based on the active site of the protein using receptor grid generation tool. Ligands prepared using LigPrep were flexibly docked in grid box using Monte Carlo-based simulation algorithm. An extra precision (XP) method was employed that generated binding poses based on energy. The favourably docked molecules were ranked according to the Glide Score (tables 3 and 4).

**Table 3.** Molecular docking analysis of 6 protein targets with selected compounds. The binding energy was calculated for Glide in kcal mol^−1^.

	Glide score binding energy (kcal mol^−1^)			
PDB target	compound **6f**	compound **6h**	compound **6o**	compound **6s**
1G3U	−6.551	−6.782	−5.617	−5.912
1W19	−6.852	−4.880	−4.165	−5.600
1ZID	−7.826	−9.189	−7.316	−11.123
2OZ5	−6.899	−7.344	−5.572	−7.967
3IUB	−6.600	−6.602	−5.104	−5.291
1DG5	−4.521	−4.793	−4.475	−6.233

**Table 4.** Molecular docking analysis of selected compounds.

protein target	compound name	amino acids involved in intermolecular interactions	binding energy (kcal mol^−1^)
1ZID	compound **6f**	Thr196	−7.826
		Phe149	
	compound **6h**	Met98	−9.189
		Arg32	
	**compound 6s**	Asp64	**−11**.**123**
		Trp222	
		Tyr158	
	compound **6o**	Thr196	−7.316

### 4.4. Molecular docking analysis

**Figure 2.**
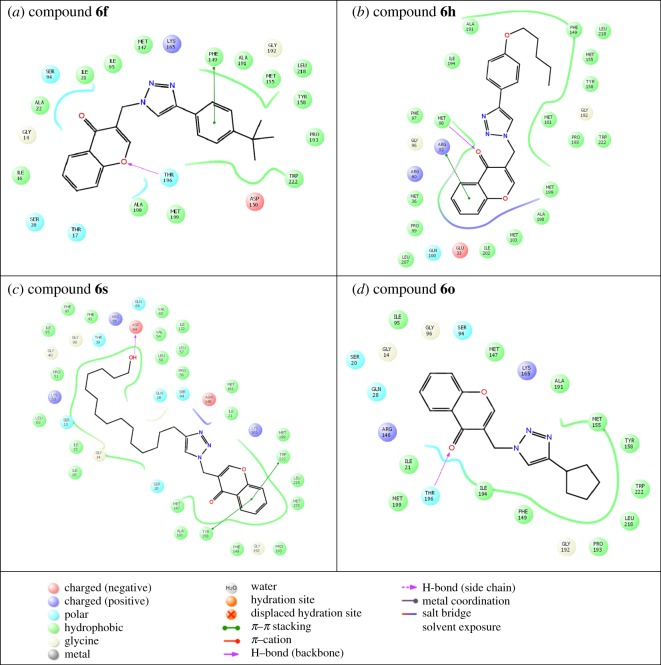
Amino acids involved in intermolecular interactions.

**Figure 3.**
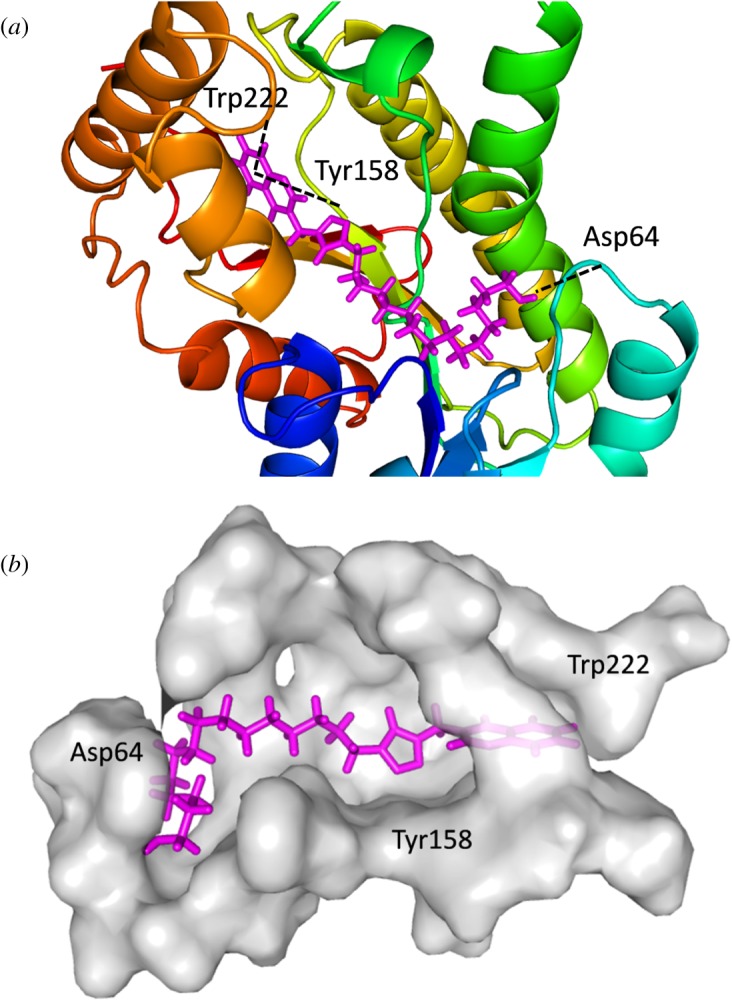
(*a*) Compound **6s** (in magenta) in enoyl-acyl carrier protein (PDB ID:1ZID). (*b*) Alignment of compound **6s** in the binding pocket.

Automated docking was used to assess the binding modes and conformation of the ligand molecules. Among the 20 chromone embedded [1,2,3]-triazoles, compounds **6f**, **6h**, **6o** and **6s** were considered as they showed significant activities (table 1). 1ZID, enoyl-acyl carrier protein yielded better binding scores with four chromone-based triazoles when compared with the rest of the proteins (table 2). Compound **6s** gave a better score when compared with other compounds for the target proteins, with binding score ranging from −7.3 to −11.123 kcal mol^−1^. Enoyl-acyl carrier protein reductase is involved in mycolic acid biosynthesis, the inhibition of which leads to the lysis of *Mtb.* The key intermolecular protein ligand interactions are depicted in figure 2. Figure 2 represents the intermolecular amino acid interaction with the compounds **6f**, **6h, 6s** and **6o**. Compound **6s** showed highest binding energy values of −11.123 kcal mol^−1^. Asp64, Trp222 and Tyr158 amino acids interacted with compound **6s** showing high ligand exposure. Trp222 and Tyr158 had π−π interaction with the compound **6s**. Compound **6f** and **6o** similarly showed π−π interaction with Phe149 and Thr196, respectively. Thus, the above results suggest that π−π interaction improves the docking scores. Compound **6s** is bound to the active site amino acid residues in the pocket region as shown in figure 3*a,b*. The pocket region of 1ZID is present in a loop region flanked by alpha helix chains seen in figure 3*a*. The location and orientation of the triazole group are complementary to the surrounding InhA side chains, which create a specific binding pocket. These observations indicate that compound **6s** may have an important role in anchoring within the active site of the receptor.

### 4.5. Chemoinformatics analysis

Six active compounds were analysed for their drug-like properties (table 5). Lipinski rule of five were predicted using Screening Assistant 2 tool [33]. All these compounds including compound **6s** displayed good drug-like properties. The drug-like and lead-like property analysis for the compounds generated a score of 0.25 which gave support to the positive results obtained in rule of five. ADME properties were predicted using PreADMET software [34] in order to check their potential as anti-tubercular compounds.

**Table 5.** Chemoinformatics analysis.

	compounds					
properties	**6f**	**6g**	**6h**	**6m**	**6o**	**6s**
**Lipinski rule**^a^						
molecular weight	359.429	333.347	389.455	283.331	295.342	453.627
HB accept	3	4	4	3	3	4
HB donor	0	0	0	0	0	1
LogP	3.335	1.792	3.631	1.533	1.409	5.188
**chemical properties**						
Weiner path^a^	2075	1661	2737	1015	1117	4787
ring count^a^	4	4	8	5	3	17
PDL/PLL^a^	0.25	0.25	0.25	0.25	0.25	0.25
**ADME properties**						
BBB (−3.0–1.2)^b^	2.88269	1.161	0.23527	0.5112	0.36551	1.14616
CaCo_2_ (nms) (<25, poor, >500, best)^b^	34.0619	23.231	32.9739	25.026	16.0711	39.8231
HIA (50–100%)^b^	97.41	97.16	97.72	98.34	98.27	96.48
rotatable bonds (0–15)^a^	4	4	8	5	3	17
TPSA (7.0–200.0)^b^	57.01	66.24	66.24	57.01	57.01	77.24
**Toxicity properties**^c^						
DSSTox carcinogenic potency mutagenecity	Neg.	Neg.	Neg.	Neg.	Neg.	Neg.
	(*p*: 0.161)	(*p*: 0.202)	(*p*: 0.112)	(*p*: 0.110)	(*p*: 0.189)	(*p*: 0.115)
DSSTox carcinogenic potency mouse	Neg.	Neg.	Neg.	Neg.	Neg.	Neg.
	(*p*: 0.083)	(*p*: 0.093)	(*p*: 0.099)	(*p*: 0.107)	(*p*: 0.141)	(*p*: 0.208)

^a^Computed using Screening Assistant 2 program. PDL, progressive drug like; PLL, progressive lead like.

^b^PreADMET software.

^c^LAZAR wherein Neg., negative and *p*, probability value.

The blood brain barrier (BBB) model values for compound **6s** was 1.14616 which clearly lay in range suggesting the compound can penetrate the BBB on theoretical grounds. Most compounds displayed CaCo_2_ cell permeability values above 25 nms [35], topological polar surface area (TPSA) above 7.0 and the human intestinal absorption (HIA) quantities in the 50–100% range, indicating that they may be further developed in an oral dosage form [36]. Lazy structure activity relationships (LAZAR) software [37] predicted all the compounds as non-carcinogenic and non-mutagenic, and the probability greater than 0.025 suggesting the predictions to be reliable. The predicted favourable ADME features for compound **6s** further indicates that it is a promising anti-tubercular lead candidate.

## 5. Conclusion

In summary, a series of novel chromone embedded [1,2,3]-triazole derivatives were synthesized via an easy and convenient synthetic protocol starting from 2-hydroxy acetophenone. The novel 20 analogues **6a–t** accomplished in four-step synthetic sequences using click chemistry in the key step were fully characterized by their NMR and mass spectral data. The *in vitro* anti-mycobacterial evaluation study of all the compounds revealed seven compounds found to be active against *M. tuberculosis* H37Rv. The compound **6s** is the most potent compound *in vitro* with a MIC value of 1.56 µg ml^−1^*.* Cross docking studies revealed compound **6s** to be more effective against the enoyl-acyl carrier protein reductase of *Mtb*. Molecular docking and chemoinformatics studies proved that compound **6s** possesses drug-like properties. Docking results indicated that Asp64, Trp222 and Tyr158 amino acids in binding pocket as potential ligand binding hot-spot residues. Further, the molecular variation as well as *in vivo* studies to prove their specificity towards *Mtb* are underway.

## 6. Experimental section

### 6.1. General methods

Solvents were purified and dried by standard procedures prior to use. ^1^H NMR and ^13^C NMR spectra were recorded on a Bruker AC-200, 400 & 500 NMR spectrometer. Spectra were obtained in CDCl_3_. Monitoring of reactions was carried out using TLC plates Merck silica gel 60 F_254_ and visualization with UV light (254 and 365 nm), I_2_ and anisaldehyde in ethanol as development reagents. Mass spectra were recorded at ionization energy 70 eV on API Q Star Pulsar spectrometer using electrospray ionization. UV detector: *λ* 280 nm; elution: mixtures of acetonitrile and water.

#### 6.1.1. 4-oxo-4*H*-chromene-3-carbaldehyde (2)

To a stirred solution of dry DMF (40 ml), POCl_3_ (20.6 ml, 220.34 mmol) was added dropwise at 5°C. The mixture was stirred for 15 min and then the solution of 2-hydroxyacetophenone (10 g, 73.44 mmol) in DMF (20 ml) was added dropwise at 5°C. The reaction mixture was stirred at the same temperature for 30 min, then heated and stirred at 55°C for another 4 h. The mixture was cooled to room temperature, poured into ice-water (approx. 400 ml) and stirred for 1.5 h. The precipitate was filtered off, washed with ethanol afforded **2;** White solid, yield = 75%; mp = 152–154°C. IR (CHCl_3_, cm^−1^) *ν*_max_ = 3370, 3023, 2921, 2403, 1659, 1612, 1569, 1464, 1423, 1310, 1217, 1027, 930, 766, 671; ^1^H NMR (400 MHz, CDCl_3_) *δ* = 7.50–7.56 (m, 2H), 7.74–7.79 (m, 1H), 8.31 (dd, *J *= 7.9, 1.6 Hz, 1H), 8.56 (s, 1H), 10.40 (s, 1H); ^13^C NMR (100 MHz, CDCl_3_) *δ* = 188.6 (CO), 175.9 (CO), 160.6 (CH), 156.2 (C), 134.8 (CH), 126.6 (CH), 126.2 (CH), 125.3 (C), 120.3 (C), 118.6 (CH); HRMS(ESI) *m/z* = Calcd for C_10_H_6_O_3_ [M + H]^+^ 175.0390, found 175.0392.

#### 6.1.2. 3-(hydroxymethyl)-4*H*-chromen-4-one (3)

To a stirred solution of 3-formyl chromone **2** (2 g, 5% of alumina weight) in 100 ml of 2-propanol, about 40 g of basic alumina was added. The resulting solution was stirred at 75°C for 4 h. The reaction mixture was filtered through celite bed and the solvent was removed under reduced pressure and the residue was purified by column chromatography on silica gel using 7 : 3 pet ether/ethyl acetate to afford compound **3;** Viscous liquid, yield = 82%; IR (CHCl_3_, cm^−1^) *ν*_max _= 3423, 3019, 2925, 2403, 1643, 1469, 1406, 1347, 1217, 1155, 1023, 971, 918, 852, 763, 670; ^1^H NMR (200 MHz, CDCl_3_) *δ* = 2.12 (bs, 1H), 4.60 (s, 2H), 7.40–7.51 (m, 2H), 7.67–7.75 (m, 1H), 7.96 (s, 1H), 8.24 (dd, *J *= 7.9, 1.7 Hz, 1H); ^13^C NMR (100 MHz, CDCl_3_) *δ* = 178.4 (CO), 156.6 (C), 152.8 (CH), 133.9 (CH), 125.6 (CH), 125.23 (CH), 123.8 (C), 123.3 (C), 118.2 (CH), 58.5 (CH_2_); HRMS(ESI) *m/z* = Calcd for C_10_H_8_O_3_ [M + H]^+^ 177.0546, found 177.0546.

#### 6.1.3. 3-(azidomethyl)-4*H*-chromen-4-one (4)

To a stirred solution of **3** (2.5 g, 14.2 mmol) and Et_3_N (5.14 ml, 36.92 mmol), methanesulfonyl chloride (1.49 ml, 18.46 mmol) in CH_2_Cl_2_ (30 ml) was added dropwise at 0°C. The resulting reaction mixture was stirred at 0°C for 1 h. After completion of the reaction (monitored by TLC), the reaction mixture was diluted with water (approx. 20 ml) and extracted with CH_2_Cl_2_ (3 × 10 ml). The combined organic layers were washed with water and brine. The organic layer was dried over Na_2_SO_4_, filtered and concentrated. The crude mesylated product **3a** (2.56 g, 71%) was further used for next step without any purification.

To a solution of crude mesylate **3a** (2.5 g, 9.84 mol) in anhydrous DMF (20 ml), sodium azide (1.6 g, 24.6 mmol) was added batchwise at room temperature. The resulting solution was heated to 50°C for 5 h. After completion of reaction (monitored by TLC) reaction mixture was poured into ice cold water (approx. 20 ml) and extracted with ethyl acetate (3 × 10 ml). The combined ethyl acetate layers were washed with brine, dried over Na_2_SO_4_, and evaporated *in vacuo*. The residue was purified by flash chromatography to affored azide **4;** White solid, yield = 93%; mp = 50–52°C; IR (CHCl_3_, cm^−1^) *ν*_max _= 3369, 3018, 2922, 2855, 2107, 1648, 1416, 1407, 1349, 1268, 1217, 1106, 1028, 842, 759, 668; ^1^H NMR (200 MHz, CDCl_3_) *δ* = 4.33 (s, 2H), 7.41–7.51 (m, 2H), 7.67–7.76 (m, 1H), 7.97 (s, 1H), 8.26 (dd, *J *= 7.9, 1.7 Hz, 1H); ^13^C NMR (50 MHz, CDCl_3_)*δ* = 176.9 (CO), 156.5 (C), 153.8 (CH), 134.0 (CH), 125.9 (CH), 125.5 (CH), 123.7 (C), 119.7 (C), 118.2 (CH), 46.4 (CH_2_); HRMS(ESI) *m/z* = Calcd for C_10_H_7_O_2_N_3_Na [M + Na]^+^ 224.0430, found 224.0432.

#### 6.1.4. General procedure for synthesis of chromone embedded [1,2,3]-triazole derivatives (6a–t)

To a stirred solution of azide **4** (1 equiv) and aliphatic/aromatic alkynes (**5a–t**) (1.3 equiv) in *t-*butanol (3 ml) was added sequentially copper sulfate pentahydrate (20 mol %), sodium ascorbate (20 mol %) and distilled water (3 ml). The resulting reaction mixture was stirred for 1–3 h at 60°C. After completion of the reaction (monitored by TLC), the reaction mixture was diluted with EtOAc (1 × 10 ml) and then washed with water (2 × 5 ml), the organic layer was separated, washed with brine solution (2 × 5 ml), dried over anhydrous sodium sulfate and concentrated *in vacuo*. The crude residue thus obtained was purified over silica gel column chromatography eluted with pet ether/ethyl acetate (1 : 1) to furnish corresponding chromone embedded [1,2,3]-triazole derivatives (**6a–t**).

#### 6.1.5. 3-((4-phenyl-1*H*-1,2,3-triazol-1-yl)methyl)-4*H*-chromen-4-one (6a)

Yellow solid; yield = 90%; mp = 154–155°C; IR (CHCl_3_, cm^−1^) *ν*_max _= 3685, 3357, 3022, 2923, 2402, 1649, 1523, 1469, 1423, 1353, 1216, 1030, 927, 765, 671; ^1^H NMR (400 MHz, CDCl_3_) *δ* = 5.48 (s, 2H), 7.30–7.34 (m, 1H), 7.39–7.51 (m, 4H), 7.70–7.74 (m, 1H), 7.83 (d, *J *= 7.3 Hz, 2H), 8.15 (s, 1H), 8.22 (s, 1H), 8.24 (dd, *J *= 8.0, 1.6 Hz, 1H); ^13^C NMR (100 MHz, CDCl_3_) *δ* = 176.7 (CO), 156.5 (C), 155.8 (CH), 134.4 (CH, 2 carbons), 130.1 (C), 128.8 (CH, 2 carbons), 128.3 (CH), 125.8 (CH, 3 carbons), 123.8 (C), 121.3 (C), 119.1 (C), 118.4 (CH, 2 carbons), 45.5 (CH_2_); HRMS(ESI) *m/z* = Calcd for C_18_H_13_O_2_N_3_ [M + H]^+^ 304.1081, found 304.1086.

#### 6.1.6. 3-((4-(*p*-tolyl)-1*H*-1,2,3-triazol-1-yl)methyl)-4*H*-chromen-4-one (6b)

White solid; yield = 82%; mp = 170–172°C; IR (CHCl_3_, cm^−1^) *ν*_max _= 3687, 3189, 3022, 2403, 2356, 1645, 1523, 1469, 1422, 1216, 1037, 927, 770, 672; ^1^H NMR (200 MHz, CDCl_3_) *δ* = 2.37 (s, 3H), 5.47 (s, 2H), 7.20 (s, 1H), 7.24 (s, 1H), 7.42–7.51 (m, 2H), 7.68–7.77 (m, 3H), 8.08 (s, 1H), 8.20 (s, 1H), 8.24 (dd, *J = *7.9, 1.6 Hz, 1H); ^13^C NMR (50 MHz, CDCl_3_) *δ* = 176.7 (CO), 156.5 (C), 155.6 (CH), 148.0 (C), 137.9 (C), 134.3 (C), 129.4 (CH, 2 carbons), 127.6 (C), 125.8 (CH), 125.8 (CH), 125.6 (CH, 2 carbons), 123.8 (C), 120.6 (CH), 119.3 (C), 118.4 (CH), 45.2 (CH_2_), 21.2 (CH_3_); HRMS(ESI) *m/z* = Calcd for C_19_H_15_O_2_N_3_ [M + H]^+^ 318.1237, found 318.1240.

#### 6.1.7. 3-((4-(4-ethylphenyl)-1*H*-1,2,3-triazol-1-yl)methyl)-4*H*-chromen-4-one (6c)

Pale yellow solid; yield = 82%; mp = 49–150°C; IR (CHCl_3_, cm^−1^) *ν*_max_ = 3687, 3394, 3022, 2403, 1648, 1529, 1424, 1217, 1030, 927, 769, 672; ^1^H NMR (200 MHz, CDCl_3_) *δ* = 1.25 (t, *J *= 7.6 Hz, 3H), 2.67 (q, *J *= 15.3, 7.6 Hz, 2H), 5.47 (s, 2H), 7.22 (s, 1H), 7.26 (s, 1H), 7.42–7.51 (m, 2H), 7.68–7.76 (m, 3H), 8.09 (s, 1H), 8.20 (s, 1H), 8.20 (s, 1H), 8.24 (dd, *J *= 7.9, 1.6 Hz, 1H); ^13^C NMR (100 MHz, CDCl_3_) *δ* = 176.7 (CO), 156.5 (C), 155.6 (CH), 148.1 (C), 144.3 (C), 134.3 (CH), 128.2 (CH, 2 carbons), 127.9 (C), 125.8 (CH), 125.8 (CH), 125.7 (CH, 2 carbons), 123.8 (C), 120.7 (CH), 119.3 (C), 118.4 (CH), 45.2 (CH_2_), 28.6 (CH_2_), 15.5 (CH_3_); HRMS(ESI): *m/z* = Calcd for C_20_H_17_O_2_N_3_ [M + H]^+^ 332.1394, found 332.1401.

#### 6.1.8. 3-((4-(4-propylphenyl)-1*H*-1,2,3-triazol-1-yl)methyl)-4*H*-chromen-4-one (6d)

White solid; yield = 84%; mp = 135–136°C; IR (CHCl_3_, cm^−1^) *ν*_max_ = 3188, 3019, 2596, 2406, 1631, 1433, 1218, 1041, 768, 671; ^1^H NMR (200 MHz, CDCl_3_) *δ* = 0.94 (t, *J *= 7.3 Hz, 3H), 1.56–1.75 (m, 4H), 2.60 (t, *J *= 7.6 Hz, 2H), 5.47 (s, 2H), 7.20 (s, 1H), 7.24 (s, 1H), 7.42–7.51 (m, 2H), 7.67–7.76 (m, 3H), 8.08 (s, 1H), 8.19 (s, 1H), 8.23 (dd, *J *= 7.9, 1.6 Hz, 1H); ^13^C NMR (100 MHz, CDCl_3_) *δ* = 176.7 (CO), 156.5 (C), 155.6 (CH), 148.1 (C), 142.7 (C), 134.3 (CH), 128.8 (CH, 2 carbons), 127.9 (C), 125.8 (CH), 125.7 (CH), 125.6 (CH, 2 carbons), 123.8 (C), 120.7 (CH), 119.3 (C), 118.4 (CH), 45.2 (CH_2_), 37.8 (CH_2_), 24.4 (CH_2_), 13.8 (CH_3_); HRMS(ESI): *m/z* = Calcd for C_21_H_19_O_2_N_3_ [M + H]^+^ 346.1550, found 346.1558.

#### 6.1.9. 3-((4-(4-pentylphenyl)-1*H*-1,2,3-triazol-1-yl)methyl)-4*H*-chromen-4-one (6e)

White solid; yield = 88%; mp = 147–148°C; IR (CHCl_3_, cm^−1^) *ν*_max _= 3370, 3022, 2926, 2403, 1648, 1524, 1466, 1421, 1353, 1216, 1029, 927, 763, 670; ^1^H NMR (200 MHz, CDCl_3_) *δ* = 0.89 (t, *J* = 6.7 Hz, 3H), 1.29–1.36 (m, 4H), 1.63–1.70 (m, 2H), 2.58–2.66 (m, 2H), 5.48 (s, 2H), 7.20 (s, 1H), 7.24 (s, 1H), 7.42–7.51 (m, 2H), 7.68–7.76 (m, 3H), 8.08 (s, 1H), 8.19 (s, 1H), 8.25 (dd, *J = *7.9, 1.6 Hz, 1H); ^13^C NMR (100 MHz, CDCl_3_) *δ* = 176.7 (CO), 156.5 (C), 155.6 (CH), 148.1 (C), 143.0 (C), 134.3 (CH), 128.8 (CH, 2 carbons), 127.8 (C), 125.8 (CH), 125.7 (CH), 125.6 (CH, 2 carbons), 123.8 (C), 120.7 (CH), 119.3 (C), 118.4 (CH), 45.2 (CH_2_), 35.6 (CH_2_), 31.4 (CH_2_), 31.0 (CH_2_), 22.5 (CH_2_), 14.0 (CH_3_); HRMS(ESI): *m/z* = Calcd for C_23_H_23_O_2_N_3_ [M + H]^+^ 374.1863, found 374.1868.

#### 6.1.10. 3-((4-(4-(*tert*-butyl)phenyl)-1*H*-1,2,3-triazol-1-yl)methyl)-4*H*-chromen-4-one (6f)

White solid; yield = 96%; mp = 218–219°C; IR (CHCl_3_, cm^−1^) *ν*_max _= 3390, 3021, 2963, 2404, 1648, 1464, 1218, 1032, 927, 769, 673; ^1^H NMR (200 MHz, CDCl_3_) *δ* = 1.34 (s, 9H), 5.48 (s, 2H), 7.41–7.51 (m, 4H), 7.68–7.78 (m, 3H), 8.10 (s, 1H), 8.20 (s, 1H), 8.24 (dd, *J *= 7.9, 1.6 Hz, 1H); ^13^C NMR (100 MHz, CDCl_3_) *δ* = 176.6 (CO), 156.5 (C), 155.6 (CH), 151.1 (C), 147.9 (C), 134.3 (CH), 127.7 (C), 125.8 (CH), 125.7 (CH), 125.6 (CH, 2 carbons), 125.4 (CH, 2 carbons), 123.8 (C), 120.7 (CH), 119.3 (C), 118.3 (CH), 45.2 (CH_2_), 34.6 (C), 31.2 (CH_3_, 3 carbons); HRMS(ESI): *m/z* = Calcd for C_22_H_21_O_2_N_3_ [M + H]^+^ 360.1707, found 360.1712.

#### 6.1.11. 3-((4-(4-methoxyphenyl)-1*H*-1,2,3-triazol-1-yl)methyl)-4*H*-chromen-4-one (6g)

White solid; yield = 60%; mp = 170–171°C; IR (CHCl_3_, cm^−1^) *ν*_max _= 3687, 3022, 2403, 2356, 1648, 1511, 1467, 1424, 1351, 1217, 1030, 927, 770, 672; ^1^H NMR (200 MHz, CDCl_3_) *δ* = 3.84 (s, 3H), 5.47 (s, 2H), 6.92 (s, 1H), 6.97 (s, 1H), 7.42–7.51 (m, 2H), 7.68–7.77 (m, 3H), 8.04 (s, 1H), 8.20 (s, 1H), 8.22–8.27 (m, 1H); ^13^C NMR (100 MHz, CDCl_3_) *δ* = 176.8 (CO), 159.5 (C), 156.5 (C), 155.7 (CH), 147.9 (C), 134.4 (CH), 127.0 (CH, 3 carbons), 125.8 (CH), 123.8 (C), 123.2 (C), 120.2 (CH), 119.3 (C), 118.3 (CH), 114.1 (CH, 2 carbons), 55.3 (CH_3_), 45.2 (CH_2_); HRMS(ESI): *m/z* = Calcd for C_19_H_15_O_3_N_3_ [M + H]^+^ 334.1186, found 334.1192.

#### 6.1.12. 3-((4-(4-(pentyloxy)phenyl)-1*H*-1,2,3-triazol-1-yl)methyl)-4*H*-chromen-4-one (6h)

White solid; yield = 85%; mp = 154–155°C; IR (CHCl_3_, cm^−1^) *ν*_max _= 3686, 3189, 3021, 2953, 2403, 1647, 1466, 1418, 1352, 1310, 1217, 1039, 926, 768, 671; ^1^H NMR (200 MHz, CDCl_3_) *δ* = 0.94 (t, *J* = 6.9 Hz, 3H), 1.38–1.50 (m, 4H), 1.77–1.86 (m, 2H), 3.98 (t, *J *= 6.6 Hz, 2H), 5.47 (s, 2H), 6.91 (s, 1H), 6.95 (s, 1H), 7.42–7.51 (m, 2H), 7.68–7.76 (m, 3H), 8.03 (s, 1H), 8.19 (s, 1H), 8.24 (dd, *J *= 7.9, 1.6 Hz, 1H); ^13^C NMR (100 MHz, CDCl_3_) *δ* = 176.7 (CO), 159.1 (C), 156.5 (C), 155.6 (CH), 147.9 (C), 134.3 (CH), 127.0 (CH, 2 carbons), 125.8 (CH), 125.7 (CH), 123.8 (C), 123.0 (C), 120.1 (CH), 119.4 (C), 118.4 (CH), 114.7 (CH, 2 carbons), 68.0 (CH_2_), 45.2 (CH_2_), 28.9 (CH_2_), 28.1 (CH_2_), 22.5 (CH_2_), 14.0 (CH_3_); HRMS(ESI): *m/z* = Calcd for C_23_H_23_O_3_N_3_ [M + H]^+^ 390.1812, found 390.1821.

#### 6.1.13. 3-((4-(*m*-tolyl)-1*H*-1,2,3-triazol-1-yl)methyl)-4*H*-chromen-4-one (6i)

Yellow solid; yield = 85%; mp = 124–125°C; IR (CHCl_3_, cm^−1^) *ν*_max _= 3685, 3190, 3021, 2403, 1646, 1523, 1468, 1418, 1352, 1217, 1043, 926, 767, 671; ^1^H NMR (200 MHz, CDCl_3_) *δ* = 2.37 (s, 3H), 5.45 (s, 2H), 7.09–7.13 (m, 1H), 7.23–7.31 (m, 1H), 7.39–7.49 (m, 2H), 7.57–7.74 (m, 3H), 8.09 (s, 1H), 8.18–8.24 (m, 2H);.^13^C NMR (100 MHz, CDCl_3_) *δ* = 176.7 (CO), 156.4 (C), 155.6 (CH), 148.0 (C), 138.4 (C), 134.3 (CH), 130.3 (C), 128.8 (CH), 128.6 (CH), 126.3 (CH), 125.7 (CH, 2 carbons), 123.7 (C), 122.8 (CH), 120.9 (CH), 119.2 (C), 118.3 (CH), 45.2 (CH_2_), 21.3 (CH_3_); HRMS(ESI): *m/z* = Calcd for C_19_H_15_O_2_N_3_ [M + H]^+^ 318.1237, found 318.1245.

#### 6.1.14. 3-((4-(4-methoxy-2-methylphenyl)-1*H*-1,2,3-triazol-1-yl)methyl)-4*H*-chromen-4-one (6j)

Yellow solid; yield = 60%; mp = 172–173°C; IR (CHCl_3_, cm^−1^) *ν*_max _= 3686, 3392, 3022, 2403, 1648, 1473, 1425, 1217, 1033, 927, 769, 672; ^1^H NMR (200 MHz, CDCl_3_) *δ* = 2.45 (s, 3H), 3.82 (s, 3H), 5.49 (s, 2H), 6.79–6.83 (m, 2H), 7.42–7.52 (m, 3H), 7.66–7.77 (m, 2H), 7.99 (s, 1H), 8.21–8.25 (m, 2H); ^13^C NMR (100 MHz, CDCl_3_) *δ* = 176.7 (CO), 159.3 (C), 156.5 (C), 155.6 (CH), 147.1 (C), 137.1 (C), 134.3 (CH), 130.1 (CH), 125.8 (CH), 125.7 (CH), 123.8 (C), 122.6 (CH), 119.4 (C, 2 carbons), 118.3 (CH), 116.1 (CH), 111.3 (CH), 55.2 (OCH_3_), 45.1 (CH_2_), 21.5 (CH_3_); HRMS(ESI): *m/z* = Calcd for C_20_H_17_O_3_N_3_ [M + H]^+^ 348.1343, found 348.1353.

#### 6.1.15. 3-((4-(naphthalen-1-yl)-1*H*-1,2,3-triazol-1-yl)methyl)-4*H*-chromen-4-one (6k)

Brick red solid; yield = 93%, mp = 154–155°C; IR (CHCl_3_, cm^−1^) *ν*_max _= 3687, 3189, 3022, 2403, 2355, 1643, 1523, 1472, 1424, 1216, 1038, 928, 770, 672; ^1^H NMR (200 MHz, CDCl_3_) *δ* = 5.56 (s, 2H), 7.42–7.56 (m, 6H), 7.68–7.77 (m, 2H), 7.86–7.91 (m, 2H), 8.22–8.26 (m, 2H), 8.28 (s, 1H), 8.36–8.41 (m, 1H); ^13^C NMR (100 MHz, CDCl_3_) *δ* = 176.7 (CO), 156.5 (C), 155.7 (CH), 147.0 (C), 134.3 (CH), 133.8 (C), 130.9 (C), 128.8 (CH), 128.3 (CH), 127.9 (C), 127.2 (CH), 126.6 (CH), 125.9 (CH), 125.8 (CH), 125.7 (CH), 125.4 (CH), 125.3 (CH), 123.9 (CH), 123.8 (C), 119.2 (C), 118.3 (CH), 45.3 (CH_2_); HRMS(ESI): *m/z* = Calcd for C_22_H_15_O_2_N_3_ [M + H]^+^ 354.1237, found 354.1246.

#### 6.1.16. 3-((4-(pyridin-2-yl)-1*H*-1,2,3-triazol-1-yl)methyl)-4*H*-chromen-4-one (6l)

Green solid; yield = 60%; mp = 175–176°C; IR (CHCl_3_, cm^−1^) *ν*_max _= 3686, 3189, 3022, 2403, 2355, 1648, 1523, 1469, 1420, 1352, 1217, 1040, 927, 770, 672; ^1^H NMR (400 MHz, CDCl_3_) *δ* = 5.52 (s, 2H), 7.32 (s, 1H), 7.43–7.50 (m, 2H), 7.69–7.73 (m, 1H), 7.84–7.93 (m, 1H), 8.16 (s, 1H), 8.23 (dd, *J *= 8.0, 1.6 Hz, 2H), 8.60 (s, 1H), 8.71 (s, 1H); ^13^C NMR (100 MHz, CDCl_3_) *δ* = 176.4 (CO), 156.5 (C), 155.4 (CH), 150.1 (C), 149.2 (C), 148.4 (C), 136.9 (CH), 134.3 (CH), 125.9 (CH), 125.8 (CH), 123.8 (CH), 123.3 (CH), 122.8 (CH), 120.3 (C), 119.1 (CH), 118.2 (CH), 45.4 (CH_2_); HRMS(ESI): *m/z* = Calcd for C_17_H_12_O_2_N_4_ [M + H]^+^ 305.1033, found 305.1038.

#### 6.1.17. 3-((4-butyl-1*H*-1,2,3-triazol-1-yl)methyl)-4*H*-chromen-4-one (6 m)

White solid; yield = 93%; mp = 87–88°C; IR (CHCl_3_, cm^−1^) *ν*_max _= 3686, 2412, 3022, 2963, 2403, 1648, 1529, 1468, 1424, 1350, 1217, 1032, 927, 769, 672; ^1^H NMR (200 MHz, CDCl_3_) *δ* = 0.92 (t, *J *= 7.1 Hz, 3H), 1.26–1.45 (m, 2H), 1.57–1.74 (m, 2H), 2.75 (t, *J *= 7.2 Hz, 2H), 5.44 (s, 2H), 7.42–7.52 (m, 2H), 7.69–7.77 (m, 2H), 8.17–8.26 (m, 2H); ^13^C NMR (100 MHz, CDCl_3_) *δ* = 176.7 (CO), 156.5 (C), 155.5 (CH), 148.7 (C), 134.3 (CH), 125.8 (CH), 125.7 (CH), 123.8 (C), 121.9 (CH), 119.5 (C), 118.3 (CH), 44.9 (CH_2_), 31.5 (CH_2_), 25.3 (CH_2_), 22.3 (CH_2_), 13.8 (CH_3_); HRMS(ESI): *m/z* = Calcd for C_16_H_17_O_2_N_3_ [M + H]^+^ 284.1394, found 284.1396.

#### 6.1.18. 3-((4-hexyl-1*H*-1,2,3-triazol-1-yl)methyl)-4*H*-chromen-4-one (6n)

White solid; yield = 82%; mp = 84–85°C; IR (CHCl_3_, cm^−1^) *ν*_max _= 3414, 3022, 2404, 1647, 1433, 1218, 1030, 928, 769, 673; ^1^H NMR (400 MHz, CDCl_3_) *δ* = 0.86 (t, *J* = 6.9 Hz, 3H), 1.26–1.37 (m, 6H), 1.61–1.68 (m, 2H), 2.67–2.71 (m, 2H), 5.40 (s, 2H), 7.43–7.49 (m, 2H), 7.64 (s, 1H), 7.69–7.73 (m, 1H), 8.15 (s, 1H), 8.22 (dd, *J *= 8.0, 1.6 Hz, 1H); ^13^C NMR (50 MHz, CDCl_3_) *δ* = 176.7 (CO), 156.5 (C), 155.6 (CH), 148.4 (C), 134.3 (CH), 125.8 (CH), 125.7 (CH), 123.8 (C), 122.2 (CH), 119.3 (C), 118.3 (CH), 45.2 (CH_2_), 31.5 (CH_2_), 29.3 (CH_2_), 28.9 (CH_2_), 25.5 (CH_2_), 22.5 (CH_2_), 14.0 (CH_3_); HRMS(ESI): *m/z* = Calcd for C_18_H_21_O_2_N_3_ [M + H]^+^ 312.1707, found 312.1711.

#### 6.1.19. 3-((4-cyclopentyl-1*H*-1,2,3-triazol-1-yl)methyl)-4*H*-chromen-4-one (6o)

Pale yellow solid; yield = 88%; mp = 157–158°C; IR (CHCl_3_, cm^−1^) *ν*_max _= 3190, 3010, 1630, 1450, 1220, 1040, 770, 670; ^1^H NMR (200 MHz, CDCl_3_) *δ* = 1.64–1.81 (m, 7H), 2.03–2.13 (m, 2H), 3.09–3.22 (m, 1H), 5.39 (s, 2H), 7.41–7.50 (m, 2H), 7.58 (s, 1H), 7.67–7.76 (m, 1H), 8.14 (s, 1H), 8.23 (dd, *J *= 7.9, 1.7 Hz, 1H); ^13^C NMR (100 MHz, CDCl_3_) *δ* = 176.7 (CO), 156.5 (C), 155.6 (CH), 152.9 (C), 134.3 (CH), 125.8 (CH), 125.7 (CH), 123.8 (C), 120.9 (CH), 119.4 (C), 118.3 (CH), 44.9 (CH_2_), 36.7 (CH), 33.1 (CH_2_, 2 carbons), 25.1 (CH_2_, 2 carbons); HRMS(ESI): *m/z* = Calcd for C_17_H_17_O_2_N_3_ [M + H]^+^ 296.1394, found 296.1398.

#### 6.1.20. 3-((4-cyclohexyl-1*H*-1,2,3-triazol-1-yl)methyl)-4*H*-chromen-4-one (6p)

Yellow solid; yield = 80%; mp = 145–146°C; IR (CHCl_3_, cm^−1^) *ν*_max _= 3188, 3020, 1632, 1433, 1218, 1042, 768, 670; ^1^H NMR (200 MHz, CDCl_3_) *δ* = 1.28–1.49 (m, 5 H), 1.70–1.85 (m, 3H), 1.96–2.07 (m, 2H), 2.64–2.80 (m, 1H), 5.38 (s, 2H), 7.41–7.50 (m, 2H), 7.56 (s, 1H), 7.67–7.75 (m, 1H), 8.12 (s, 1H), 8.23 (dd, *J *= 7.9, 1.6 Hz, 1H); ^13^C NMR (100 MHz, CDCl_3_) *δ* = 176.7 (CO), 156.5 (C), 155.6 (CH), 153.9 (C), 134.2 (CH), 125.8 (CH), 125.7 (CH), 123.8 (C), 120.6 (CH), 119.4 (C), 118.3 (CH), 44.9 (CH_2_), 35.3 (CH), 32.9 (CH_2_, 2 carbons), 26.1 (CH_2_, 2 carbons), 25.9 (CH_2_); HRMS(ESI): *m/z* = Calcd for C_18_H_19_O_2_N_3_ [M + H]^+^ 310.1550, found 310.1557.

#### 6.1.21. 3-((4-(cyclohexylmethyl)-1*H*-1,2,3-triazol-1-yl)methyl)-4*H*-chromen-4-one (6q)

White solid; yield = 88%; mp = 133–134°C; IR (CHCl_3_, cm^−1^) *ν*_max _= 3686, 3189, 3021, 2928, 2853, 2403, 1647, 1524, 1465, 1417, 1350, 1216, 1043, 926, 768, 671; ^1^H NMR (200 MHz, CDCl_3_) *δ* = 0.89–1.03 (m, 2H), 1.14–1.28 (m, 3H), 1.60–1.72 (m, 6H), 2.56 (d, *J *= 6.7 Hz, 2H), 5.39 (s, 2H), 7.41–7.51 (m, 2H), 7.59 (s, 1H) 7.67–7.76 (m, 1H), 8.13 (s, 1H), 8.23 (dd, *J *= 8.0, 1.6 Hz, 1H); ^13^C NMR (100 MHz, CDCl_3_) *δ* = 176.7 (CO), 156.5 (C), 155.5 (CH), 147.2 (C), 134.3 (CH), 125.8 (CH), 125.7 (CH), 123.8 (C), 122.5 (CH), 119.5 (C), 118.3 (CH), 44.9 (CH_2_), 38.0 (CH), 33.4 (CH_2_), 33.0 (CH_2_, 2 carbons), 26.4 (CH_2_), 26.1 (CH_2_, 2 carbons); HRMS(ESI): *m/z* = Calcd for C_19_H_21_O_2_N_3_ [M + H]^+^ 324.1707, found 324.1710.

#### 6.1.22. 3-((4-(9-hydroxy-9*H*-fluoren-9-yl)-1*H*-1,2,3-triazol-1-yl)methyl)-4*H*-chromen-4-one (6r)

Pale yellow solid; yield = 58%; mp = 239–240°C; IR (CHCl_3_, cm^−1^) *ν*_max _= 3687, 3188, 3022, 2403, 1645, 1522, 1467, 1421, 1216, 1043, 926, 769, 671; ^1^H NMR (200 MHz, CDCl_3_) *δ* = 1.74 (bs, 1H), 5.35 (s, 2H), 7.30 (s, 1H), 7.33–7.53 (m, 6H), 7.61–7.74 (m, 5H), 8.13 (s, 1H), 8.14–8.19 (m, 1H); ^13^C NMR (100 MHz, CDCl_3_) *δ* = 176.5 (CO), 156.5 (C), 155.7 (CH), 147.6 (C, 2 carbons), 139.6 (C, 2 carbons), 134.3 (CH), 129.7 (C), 129.5 (CH, 2 carbons), 128.5 (C), 128.3 (CH, 2 carbons), 125.9 (CH), 125.7 (CH), 124.9 (CH), 123.7 (C), 120.3 (CH, 2 carbons), 120.2 (CH, 2 carbons), 119.0 (C), 118.3 (CH), 45.0 (CH_2_); HRMS(ESI): *m/z* = Calcd for C_25_H_17_O_3_N_3_ [M + H]^+^ 408.1343, found 408.1349.

#### 6.1.23. 3-((4-(15-hydroxypentadecyl)-1*H*-1,2,3-triazol-1-yl)methyl)-4*H*-chromen-4-one (6s)

Pale yellow solid; yield = 84%; mp = 123–124°C; IR (CHCl_3_, cm^−1^) *ν*_max _= 3688, 3391, 3023, 2930, 2403, 2354, 1648, 1524, 1427, 1216, 1026, 928, 768, 671; ^1^H NMR (200 MHz, CDCl_3_) *δ* = 1.25–1.37 (m, 22H), 1.57–1.72 (m, 4H), 2.68 (t, *J *= 6.57 Hz, 2H), 3.65 (t, *J *= 6.6 Hz, 2H), 5.39 (s, 2H), 7.41–7.51 (m, 2H), 7.61 (s, 1H), 7.68–7.76 (m, 1H), 8.13 (s, 1H), 8.23 (dd, *J *= 7.9, 1.6 Hz, 1H); ^13^C NMR (100 MHz, CDCl_3_) *δ* = 176.7 (CO), 156.5 (C), 155.5 (CH), 148.7 (C), 134.3 (CH), 125.8 (CH), 125.7 (CH), 123.8 (C), 121.9 (CH), 119.5 (C), 118.4 (CH), 63.0 (CH_2_), 44.9 (CH_2_), 32.8 (CH_2_), 29.6 (CH_2_, 7 carbons), 29.5 (CH_2_), 29.4 (CH_2_), 29.3 (CH_2_), 29.2 (CH_2_), 25.7 (CH_2_), 25.6 (CH_2_); HRMS(ESI): *m/z* = Calcd for C_27_H_39_O_3_N_3_ [M + H]^+^ 454.3064, found 454.3074.

#### 6.1.24. 2-((1*H*-1,2,3-triazol-1-yl)methyl)-4*H*-chromen-4-one (6t)

Pale yellow solid; yield = 38%; mp = 117–118°C; IR (CHCl_3_, cm^−1^) *ν*_max _= 3687, 3412, 3022, 2403, 2356, 1649, 1523, 1470, 1421, 1216, 1069, 1025, 927, 770, 672; ^1^H NMR (200 MHz, CDCl_3_) *δ* = 5.47 (s, 2H), 7.42–7.51 (m, 2H), 7.69–7.76 (m, 2H), 7.92 (s, 1H), 8.16 (s, 1H), 8.23 (dd, *J *= 7.9, 1.6 Hz, 1H); ^13^C NMR (100 MHz, CDCl_3_) *δ* = 176.7 (CO), 156.5 (C), 155.5 (CH), 134.3 (CH), 133.9 (CH), 125.8 (CH, 2 carbons), 124.8 (CH), 123.8 (C), 119.3 (C), 118.4 (CH), 45.0 (CH_2_); HRMS(ESI): *m/z* = Calcd for C_12_H_9_O_2_N_3_ [M + H]^+^ 228.0768, found 228.0771.

## Supplementary Material

Identification of potent chromone embedded [1,2,3]-triazoles as novel anti-tubercular agents

## Data accessibility

The datasets supporting this article have been uploaded as part of the electronic supplementary material.
