# Supplementary material for: Identification of potent chromone embedded [1,2,3]-triazoles as novel anti-tubercular agents
Source: R Soc Open Sci. 2018 Apr 4;5(4):171750. doi: 10.1098/rsos.171750 (PMC5936909; doi:10.1098/rsos.171750)
Supplement: Identification of potent chromone embedded [1,2,3]-triazoles as novel anti-tubercular agents [file rsos171750supp1.pdf]

Supporting Information

Identification of potent chromone embedded [1,2,3]-triazoles as novel antitubercular agents

Viswanadh Nalla,[1,2] Aslam Shaikh,[1,2] Sanket Bapat,[4] Renu Vyas,[4] M. Karthikeyan,[1,2] P. Yogeeswari,[3] D. Sriram,[3] M. Muthukrishnan*[1,2]

[1]*Division of Organic Chemistry, National Chemical Laboratory, Pune 411 008, India*

[2]*Academy of Scientific and Innovative Research (AcSIR), New Delhi 110 025, India*

[3]*Tuberculosis Drug Discovery Laboratory, Pharmacy Group, Birla Institute of Technology & Science-Pilani, Hyderabad Campus, Hyderabad 500 0078, India*

[4]*MIT School of Bioengineering Sciences and Research, MIT Art, Design and Technology University, Pune 412 201, INDIA*

**Corresponding author. Tel.: +91 20 25902284; fax: +91 2025902629;*

E-mail: address: m.muthukrishnan@ncl.res.in (M. Muthukrishnan)

S.No.	Table of contents	Pages
1	^{1}H, ^{13}C & HRMS of compound *2*	S3-S5
2	^{1}H, ^{13}C & HRMS of compound *3*	S6-S8
3	^{1}H, ^{13}C & HRMS of compound *4*	S9-S11
4	^{1}H, ^{13}C & HRMS of compound *6a*	S12-S14
5	^{1}H, ^{13}C & HRMS of compound *6b*	S15-S17
6	^{1}H, ^{13}C & HRMS of compound *6c*	S18-S20
7	^{1}H, ^{13}C & HRMS of compound *6d*	S21-S23
8	^{1}H, ^{13}C & HRMS of compound *6e*	S24-S26
9	^{1}H, ^{13}C & HRMS of compound *6f*	S27-S29
10	^{1}H, ^{13}C & HRMS of compound *6g*	S30-S32
11	^{1}H, ^{13}C & HRMS of compound *6h*	S33-S35
12	^{1}H, ^{13}C & HRMS of compound *6i*	S36-S38
13	^{1}H, ^{13}C & HRMS of compound *6j*	S39-S41

**14**	**^{1}H, ^{13}C & HRMS of compound *6k***	**S42-S44**
**15**	**^{1}H, ^{13}C & HRMS of compound *6l***	**S45-S47**
**16**	**^{1}H, ^{13}C & HRMS of compound *6m***	**S48-S50**
**17**	**^{1}H, ^{13}C & HRMS of compound *6n***	**S51-S53**
**18**	**^{1}H, ^{13}C & HRMS of compound *6o***	**S54-S56**
**19**	**^{1}H, ^{13}C & HRMS of compound *6p***	**S57-S59**
**20**	**^{1}H, ^{13}C & HRMS of compound *6q***	**S60-S62**
**21**	**^{1}H, ^{13}C & HRMS of compound *6r***	**S63-S65**
**22**	**^{1}H, ^{13}C & HRMS of compound *6s***	**S66-S68**
**23**	**^{1}H, ^{13}C & HRMS of compound *6t***	**S69-S71**
**24**	***In-vitro* MTB MABA and cytotoxicity assay**	**S72**
**25**	**Correlation of bioactivity with respect to GLIDE Score Binding Energy**	**S73**

^{1}H NMR (400 MHz, $CDCl_3$) of compound ***2***:

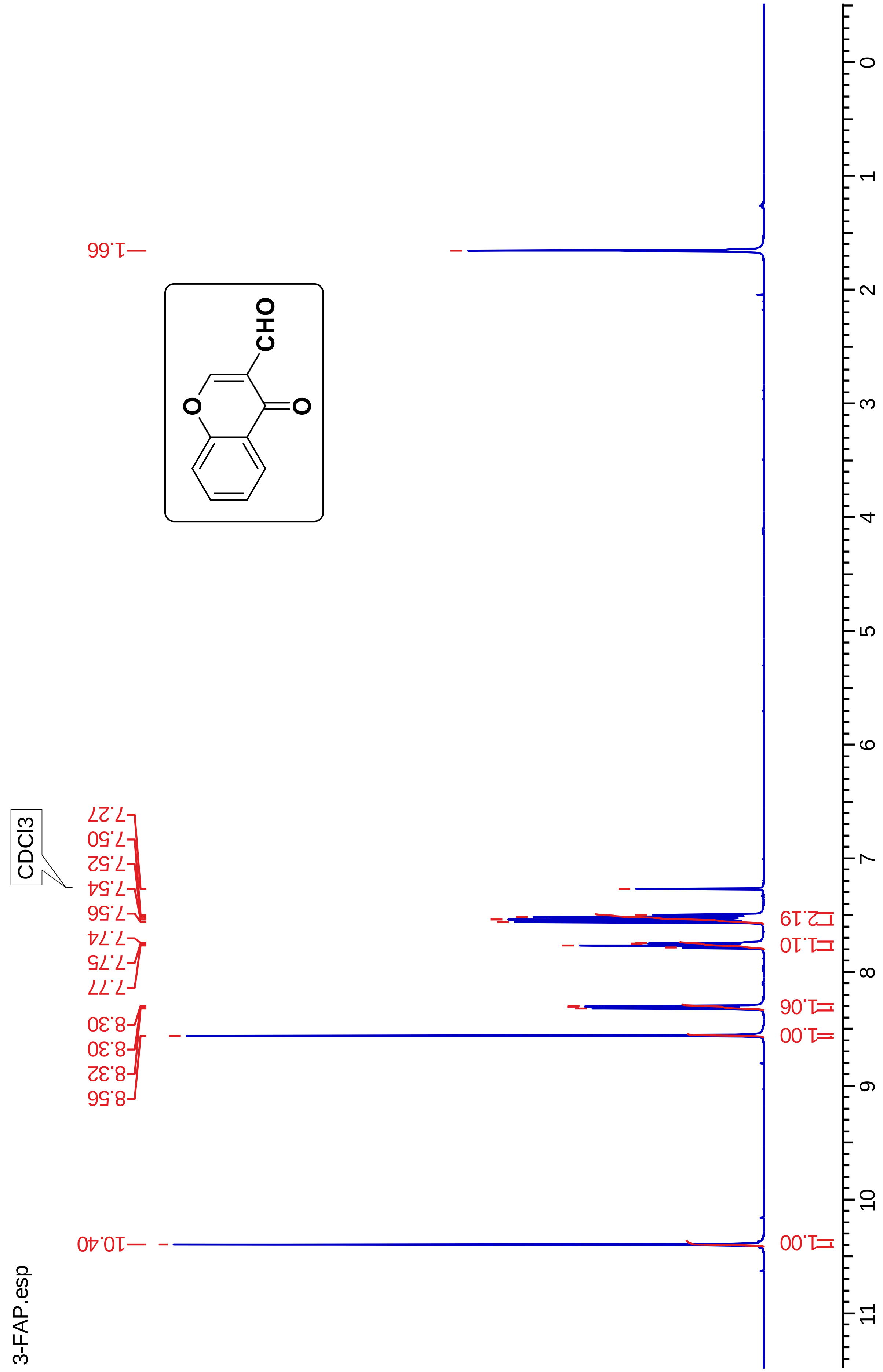

^{13}C NMR (100 MHz, $CDCl_3$) of compound ***2***:

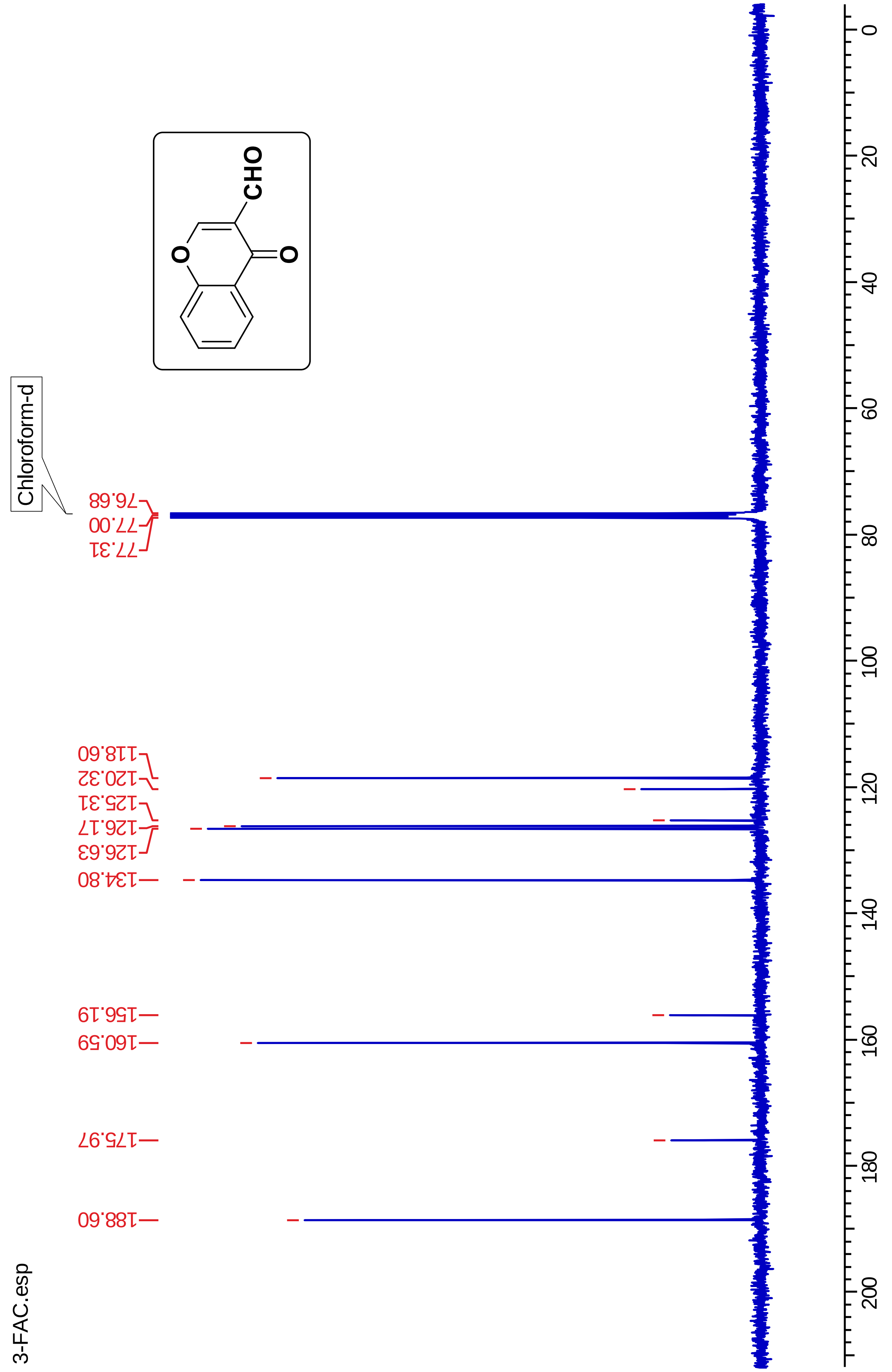

HRMS of compound ***2***:

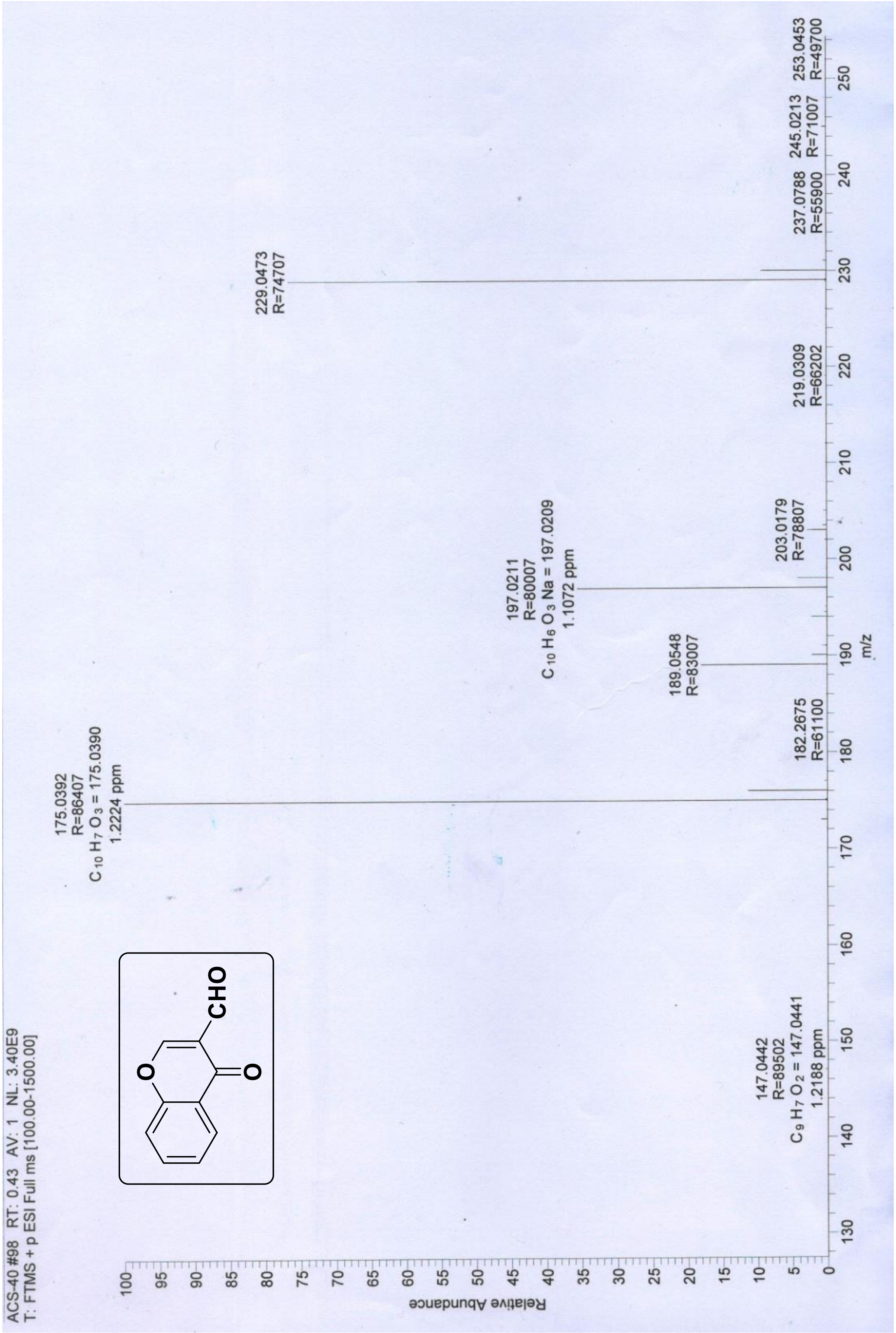

^{1}H NMR (400 MHz, $CDCl_3$) of compound **3**:

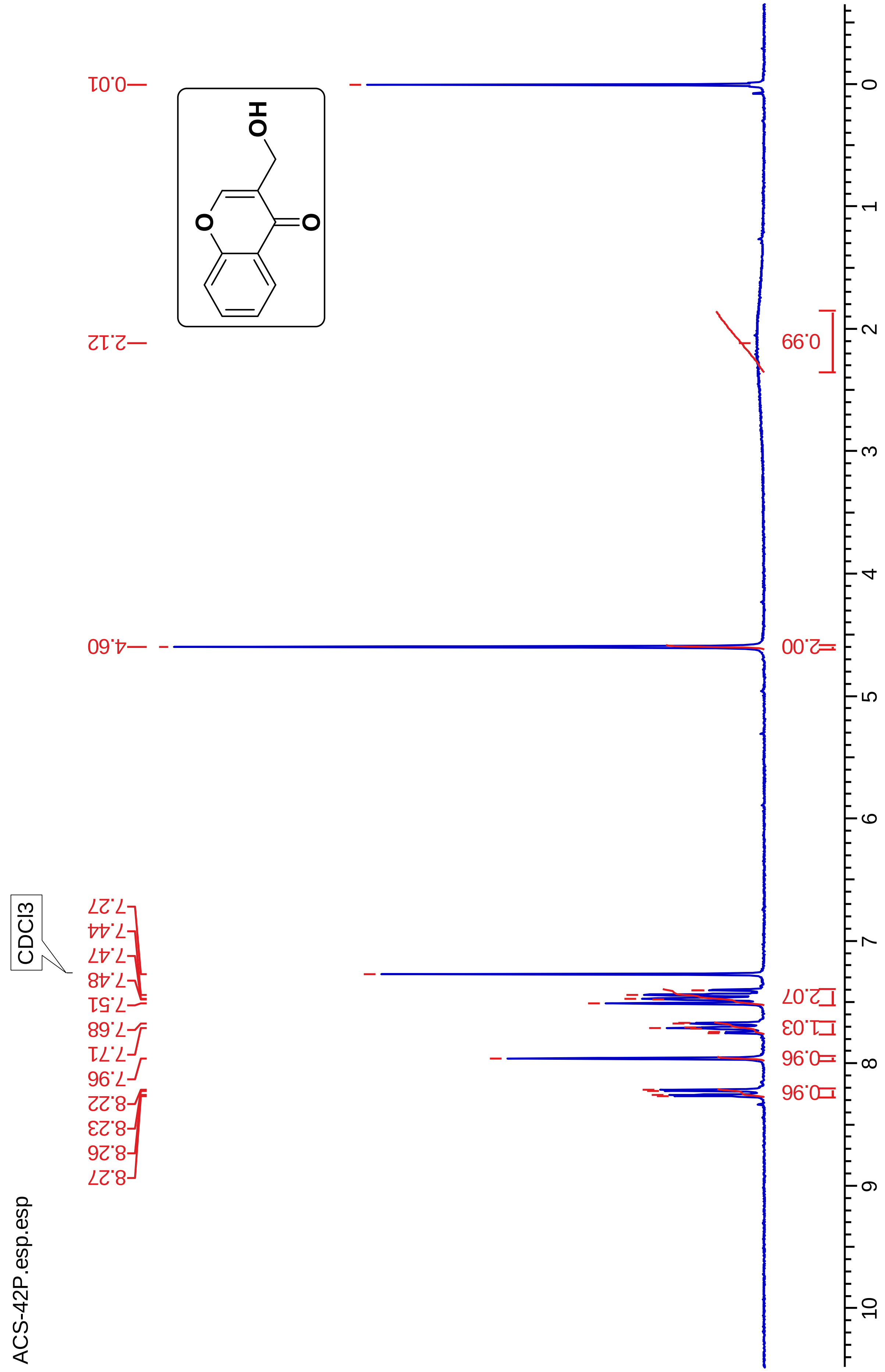

^{13}C NMR (100 MHz, $CDCl_3$) of compound ***3***:

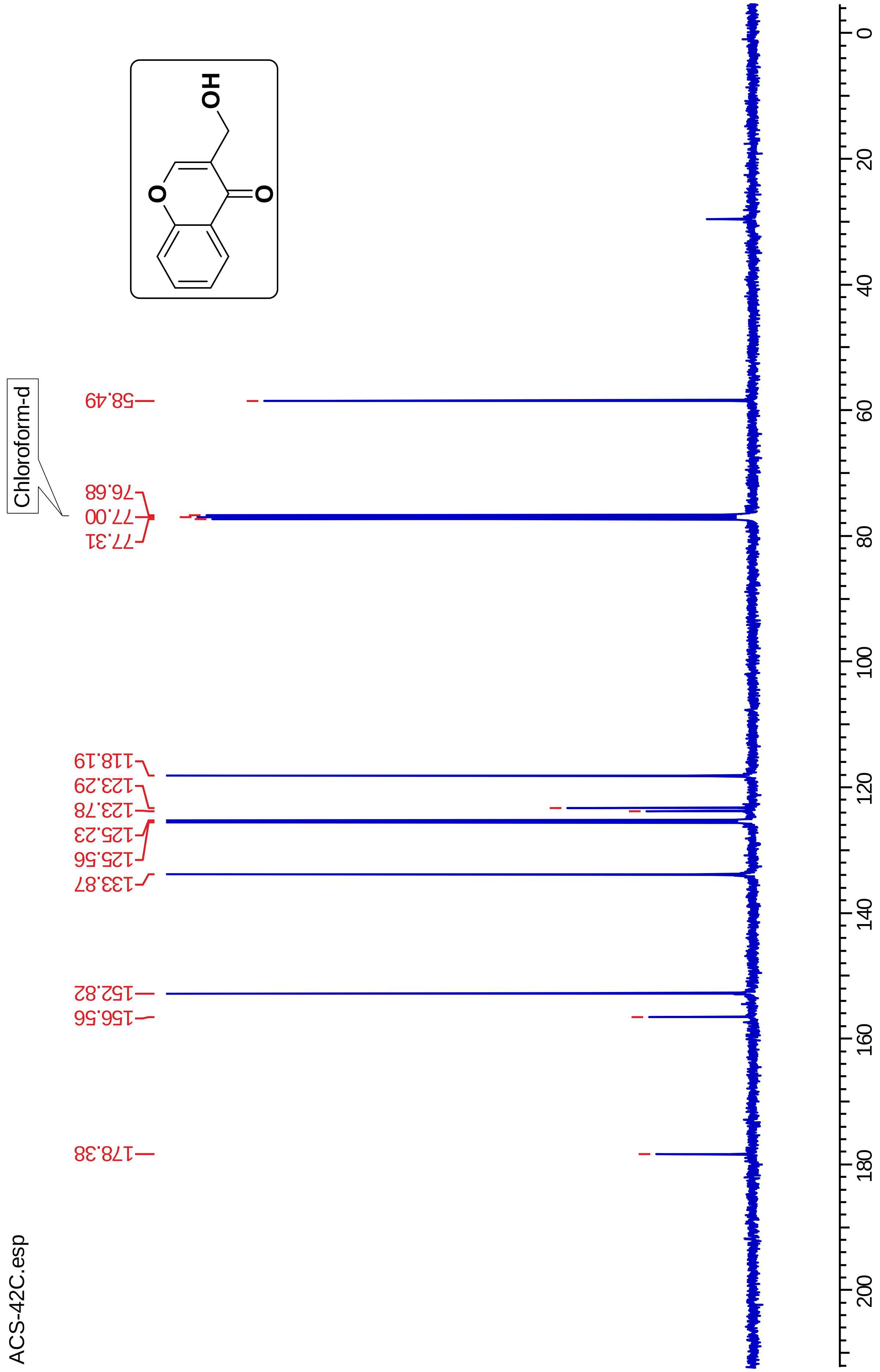

HRMS of compound ***3***:

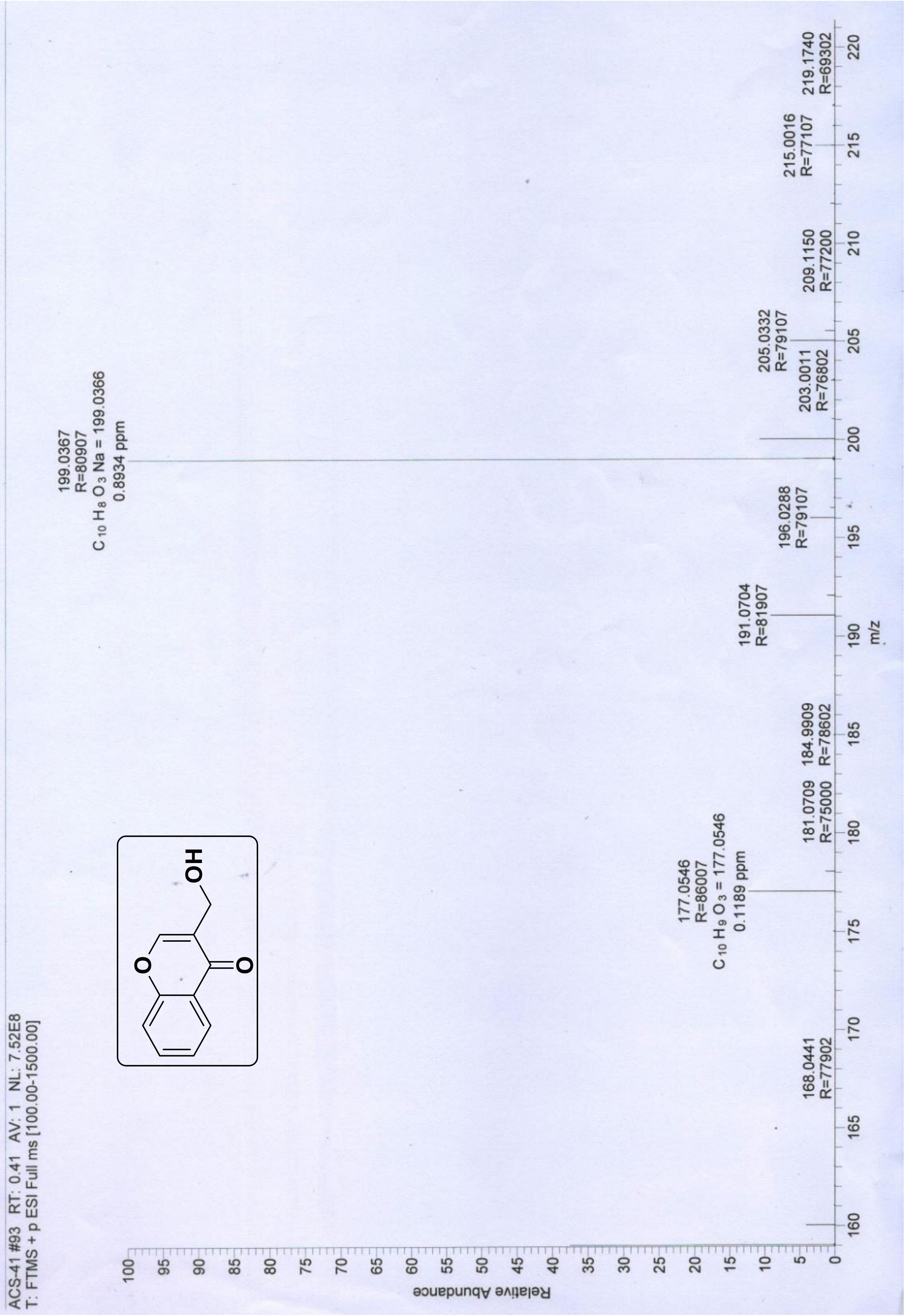

^{1}H NMR (400 MHz, $CDCl_3$) of compound ***4***:

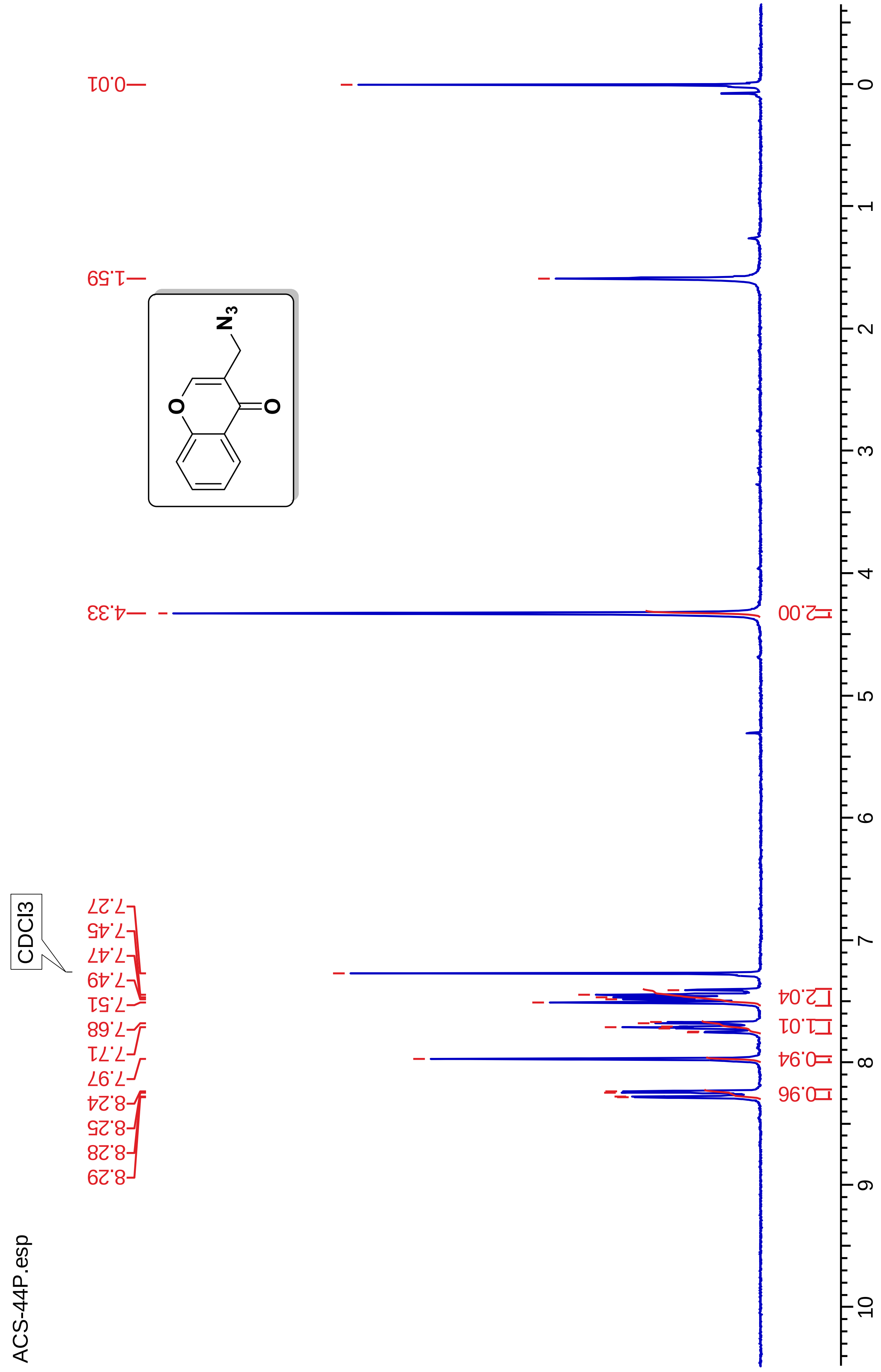

^{13}C NMR (100 MHz, $CDCl_3$) of compound ***4***:

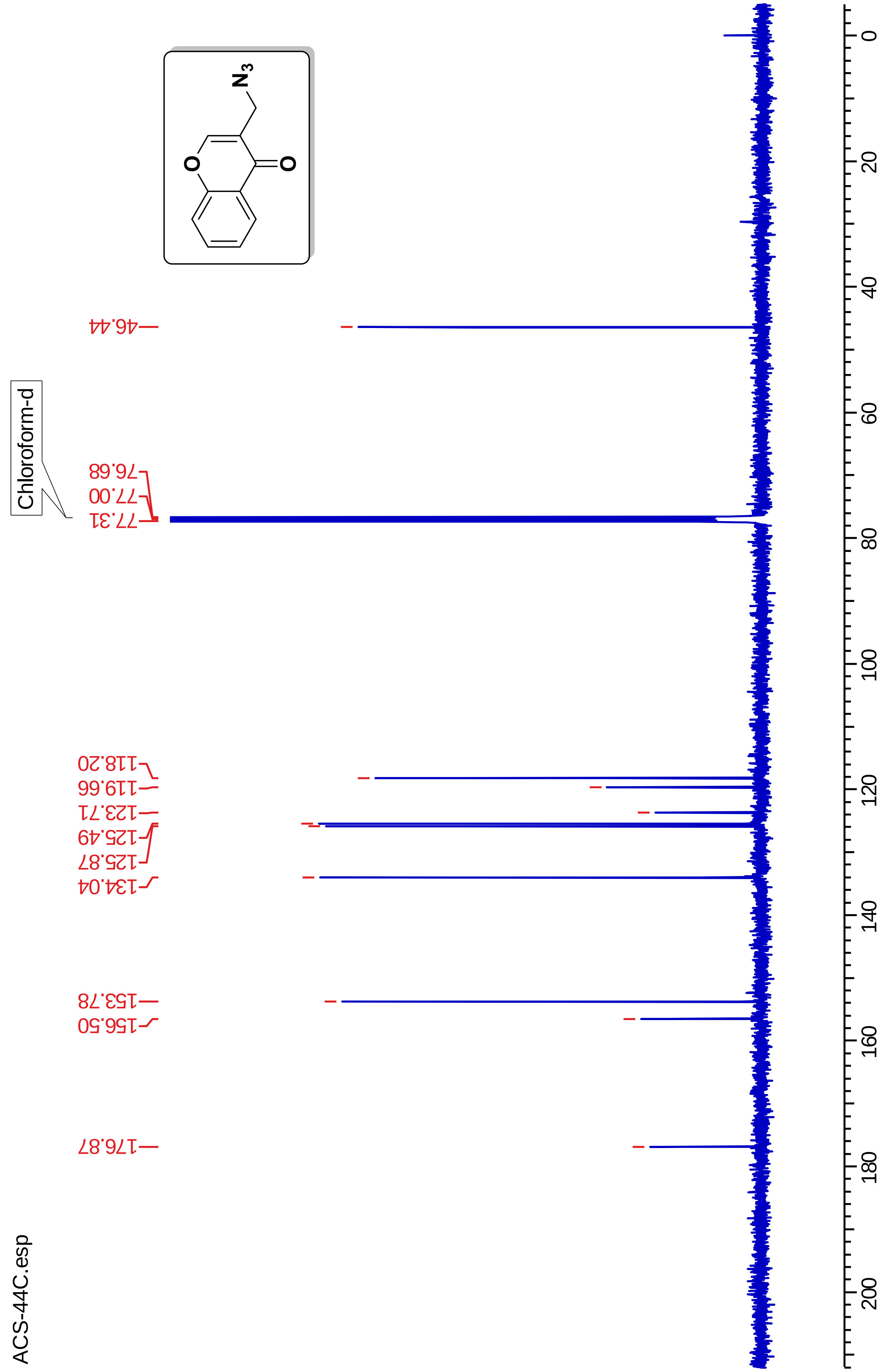

HRMS of compound ***4***:

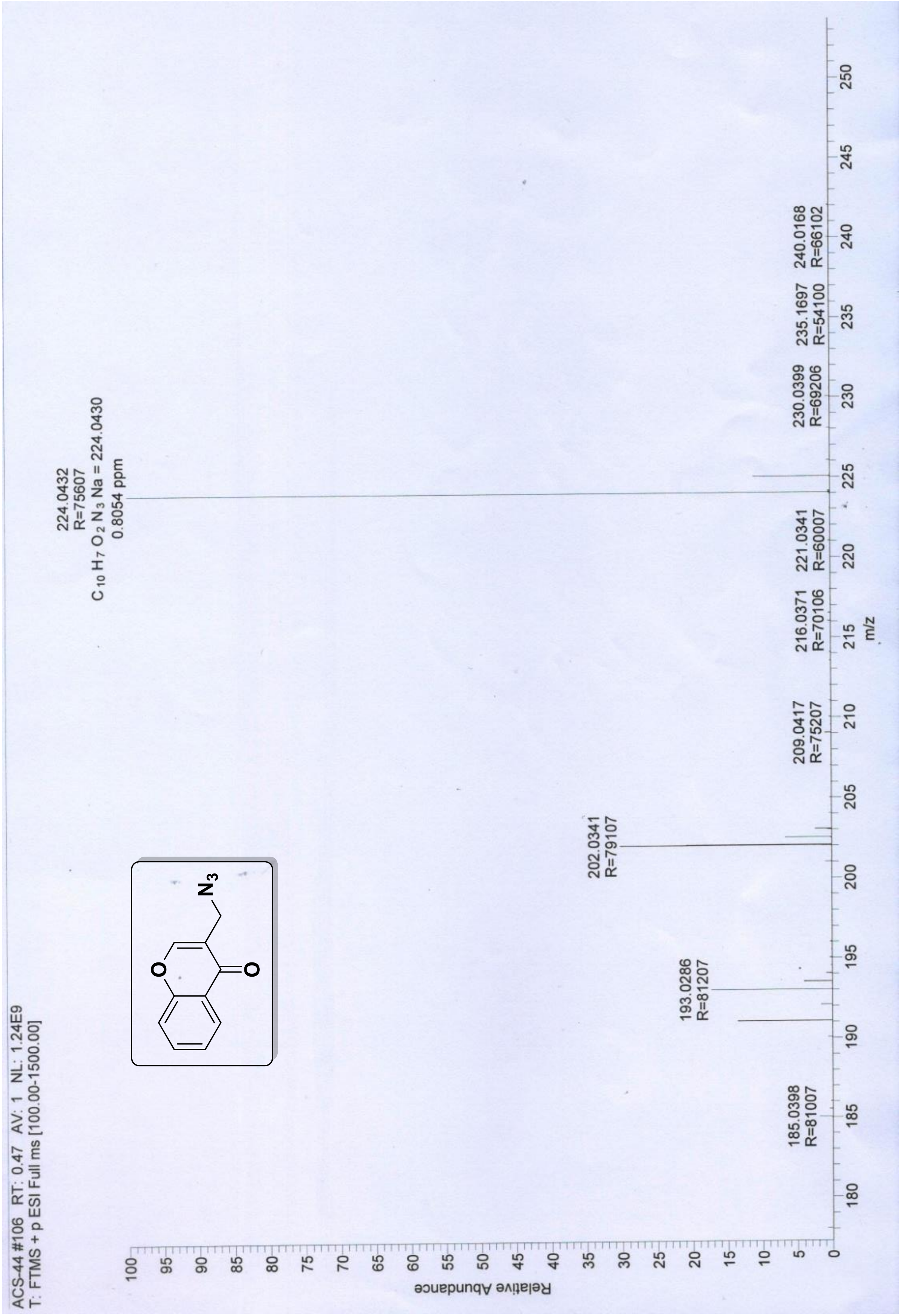

^{1}H NMR (400 MHz, $CDCl_3$) of compound ***6a***:

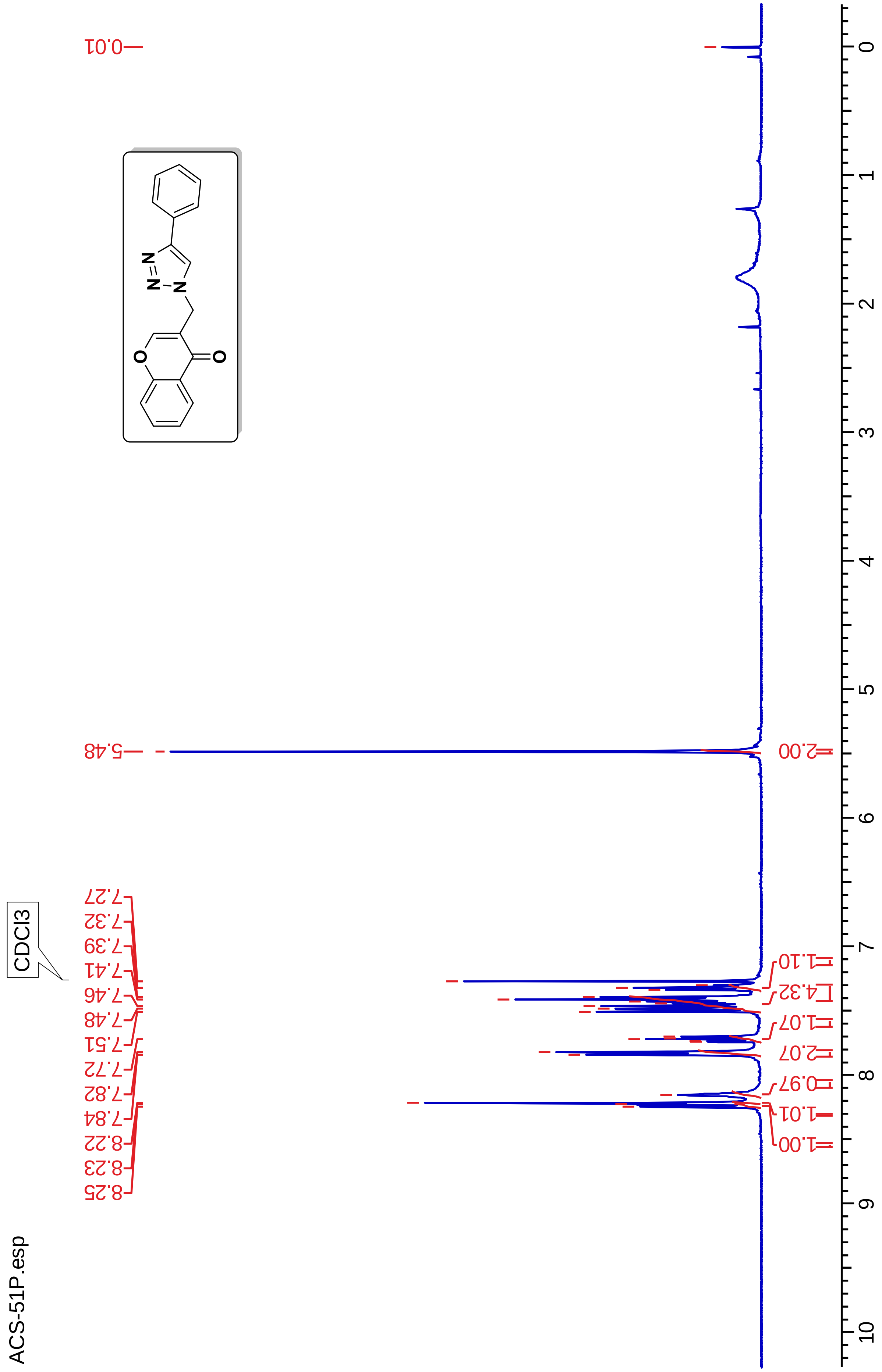

^{13}C NMR (100 MHz, $CDCl_3$) of compound ***6a***:

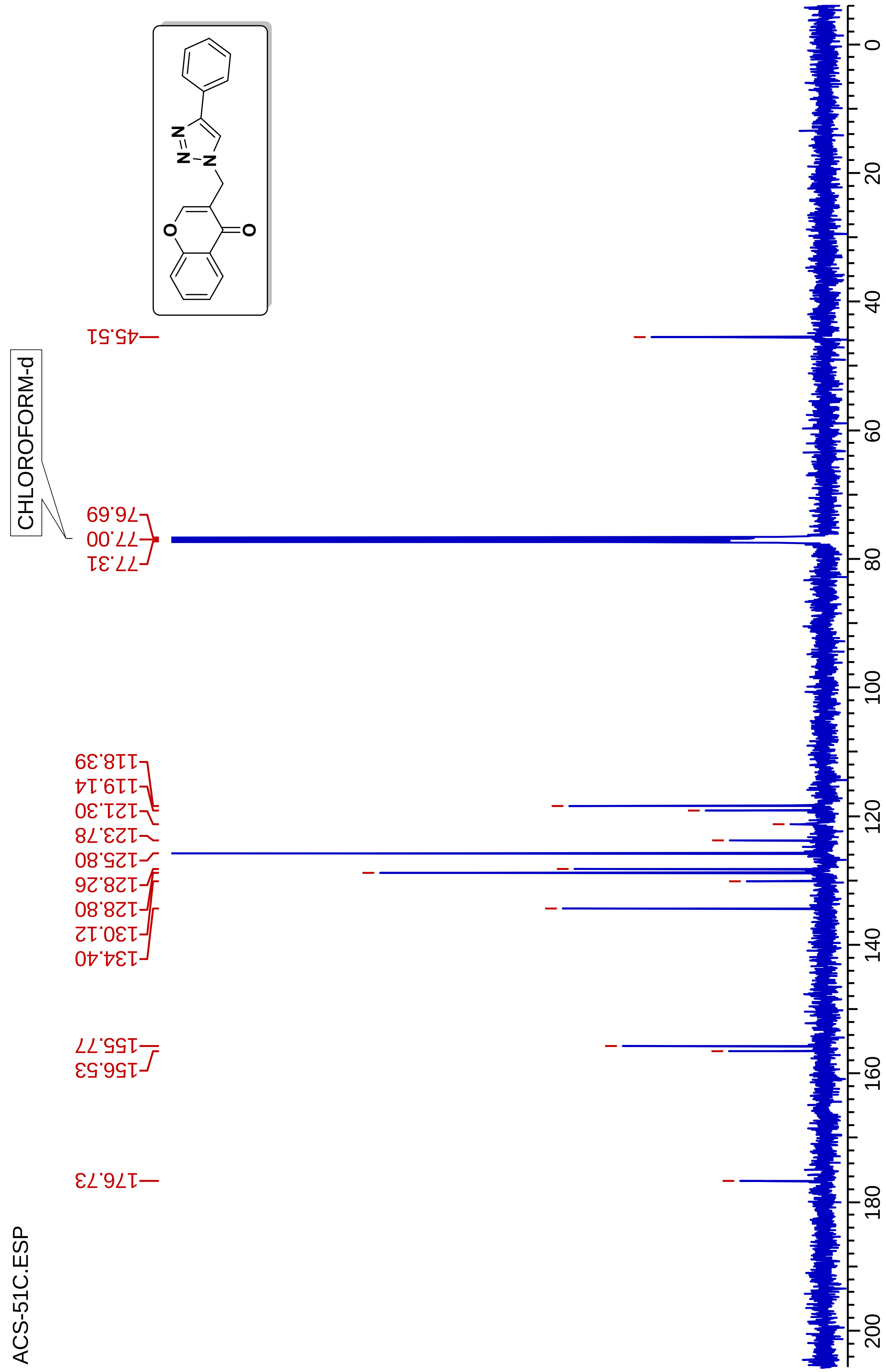

HRMS of compound ***6a***:

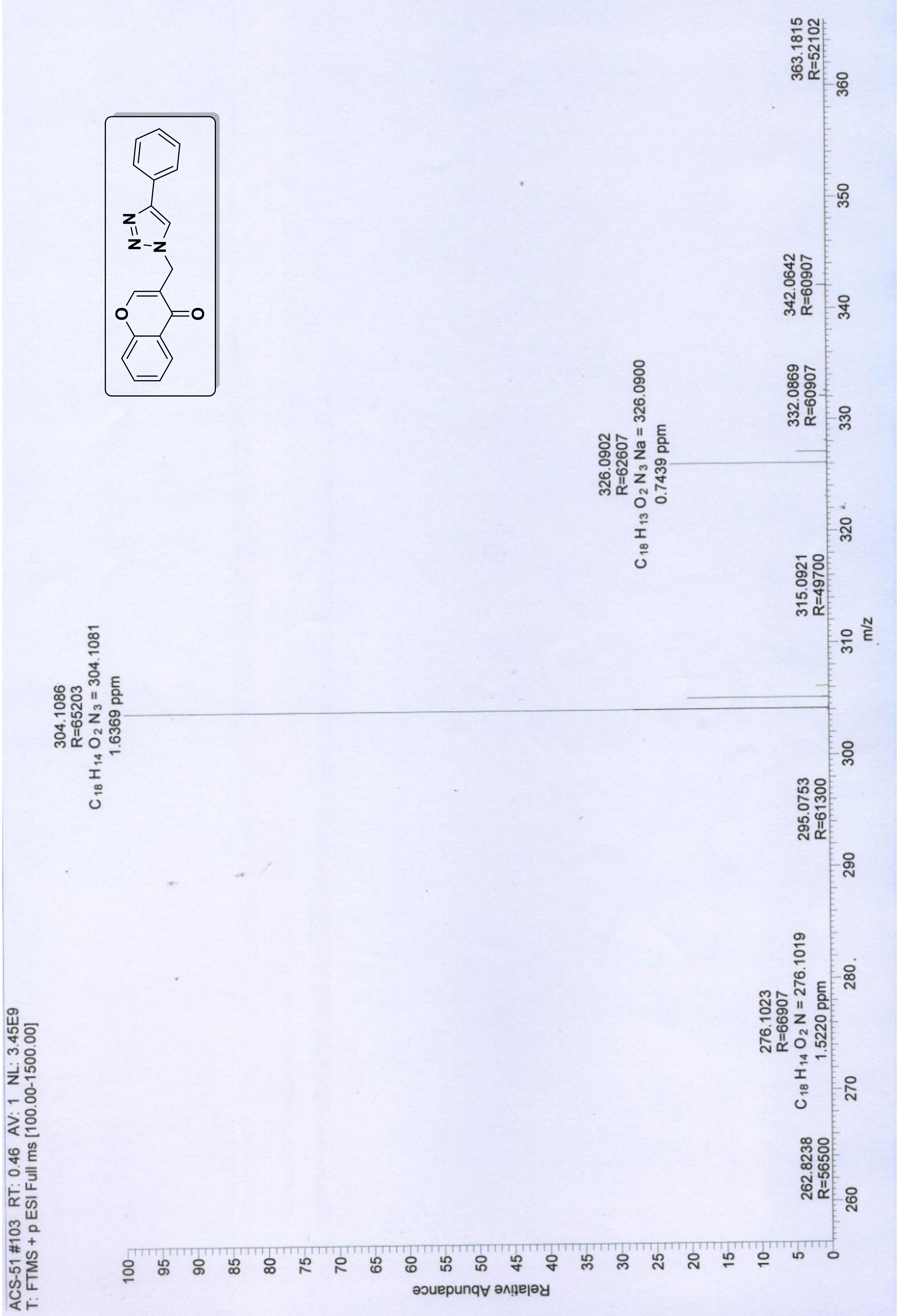

^{1}H NMR (400 MHz, $CDCl_3$) of compound ***6b***:

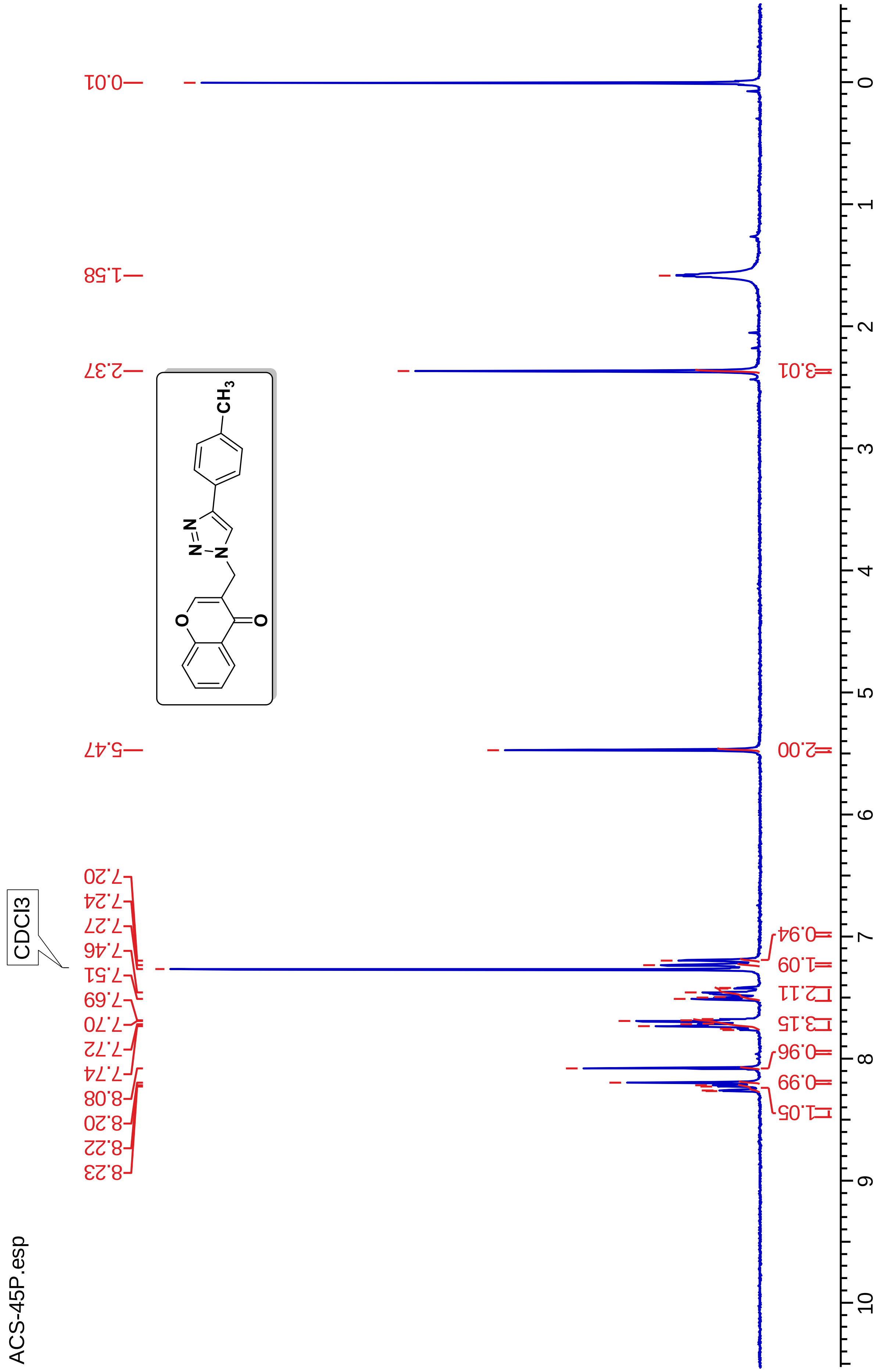

^{13}C NMR (100 MHz, $CDCl_3$) of compound ***6b***:

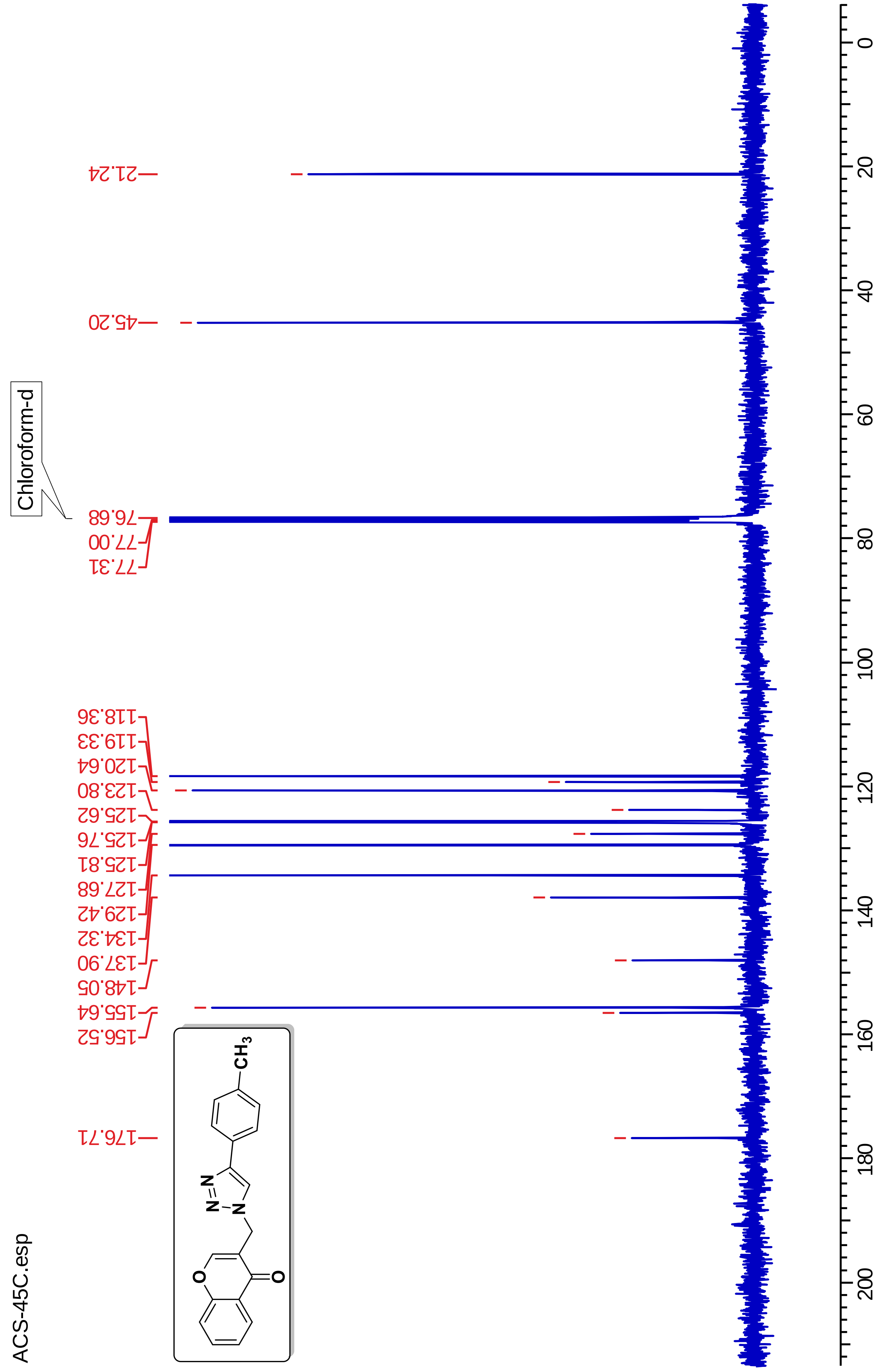

HRMS of compound ***6b***:

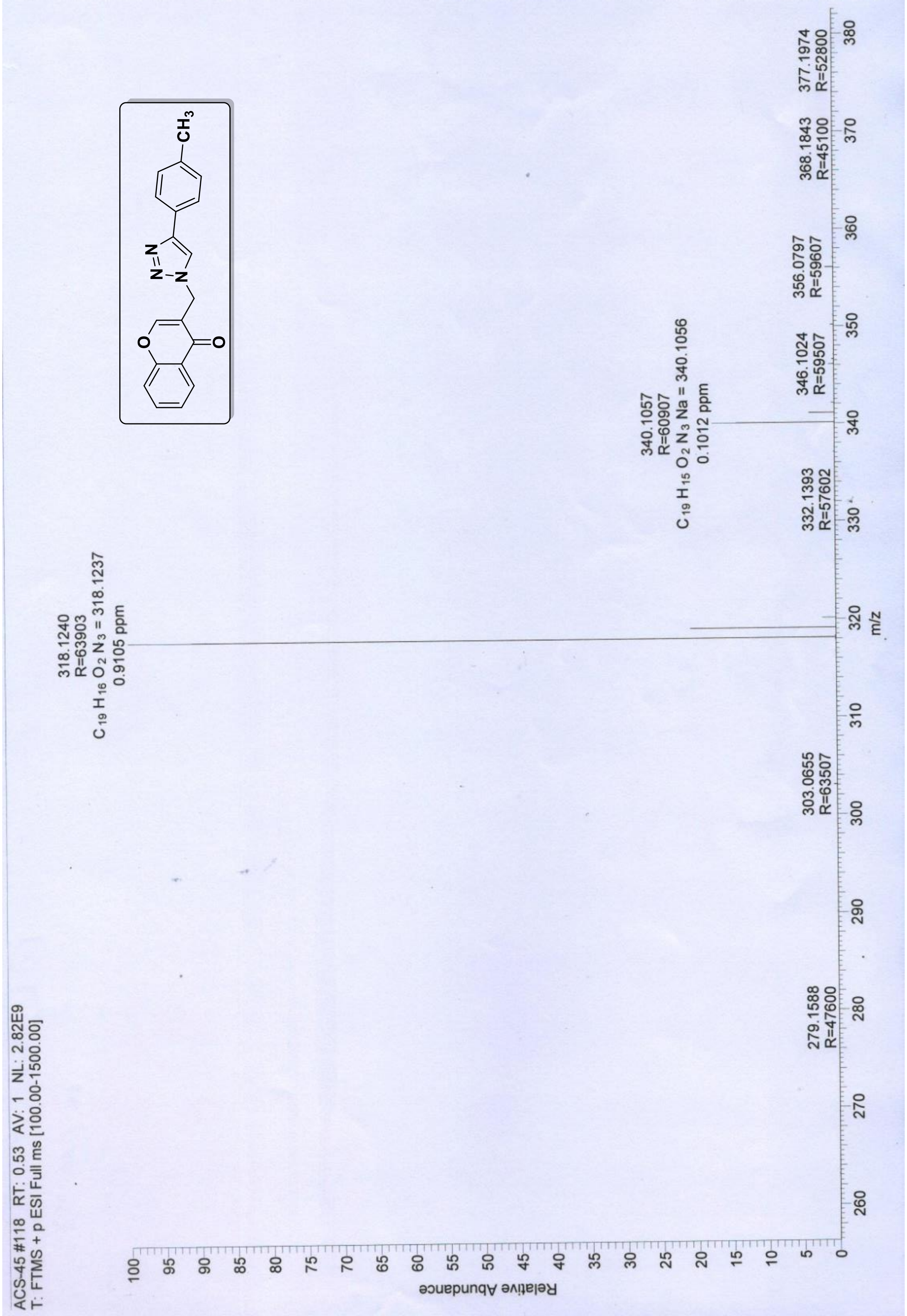

^{1}H NMR (400 MHz, $CDCl_3$) of compound ***6c***:

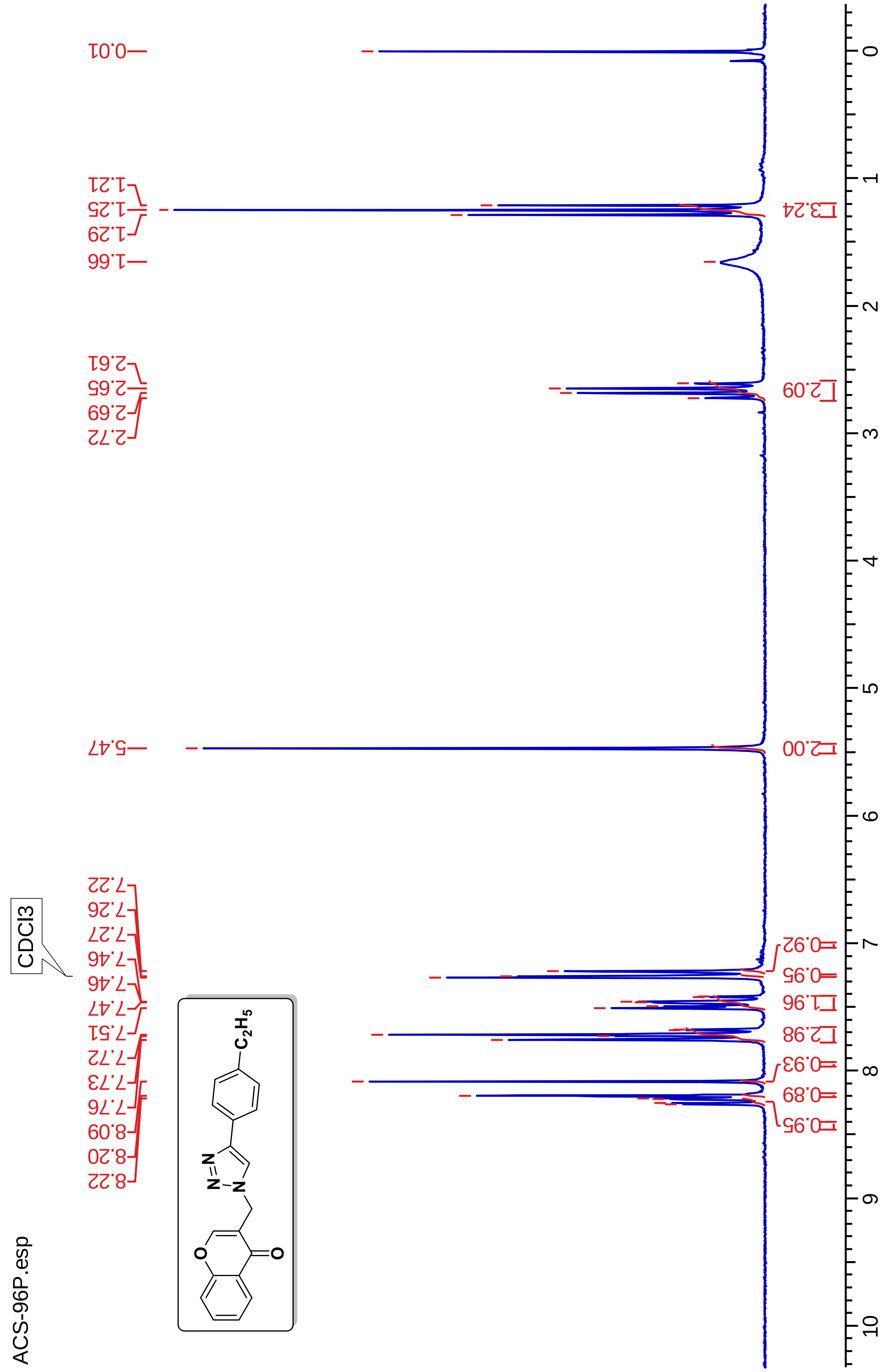

^{13}C NMR (100 MHz, $CDCl_3$) of compound ***6c***:

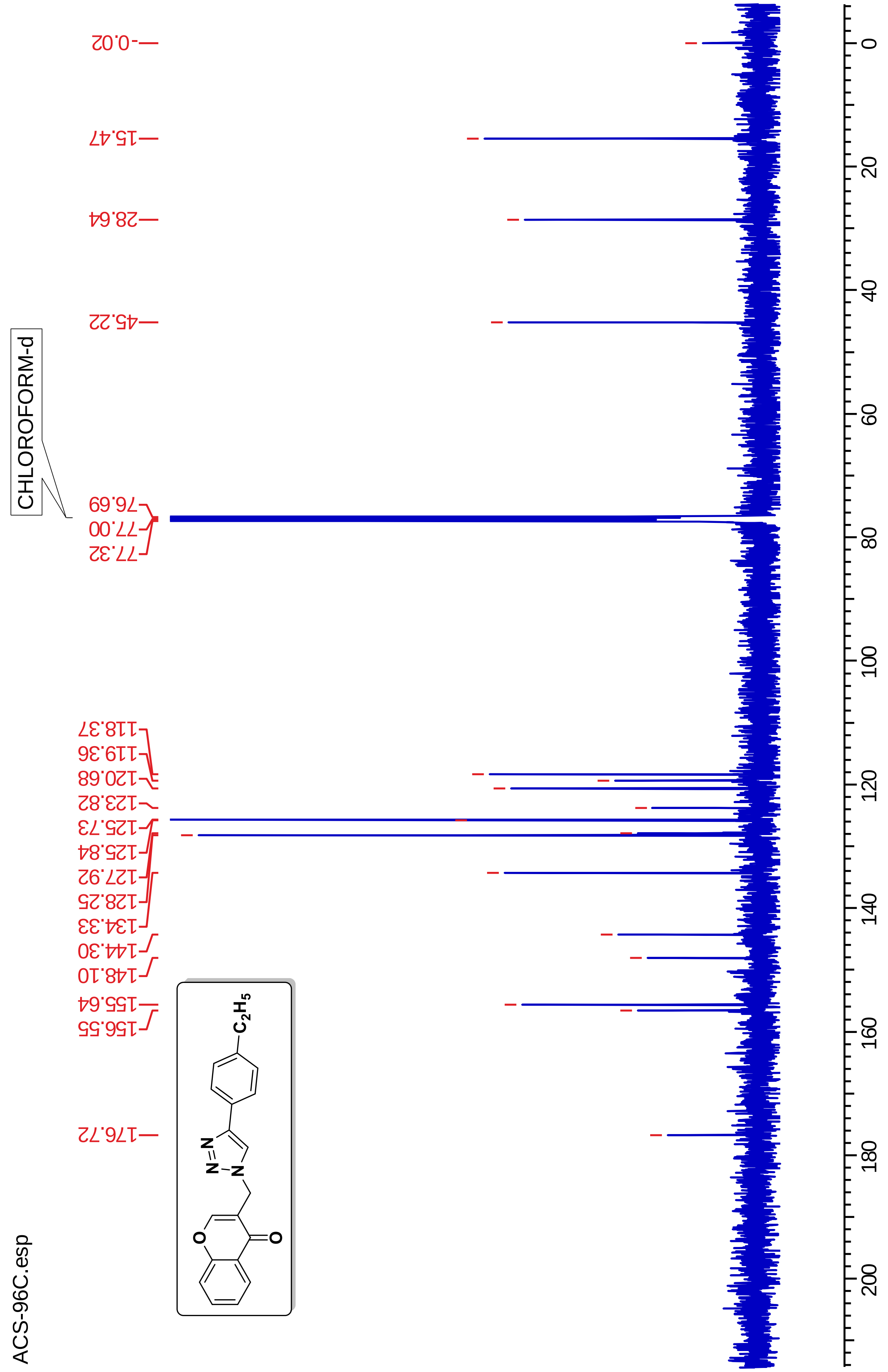

HRMS of compound ***6c***:

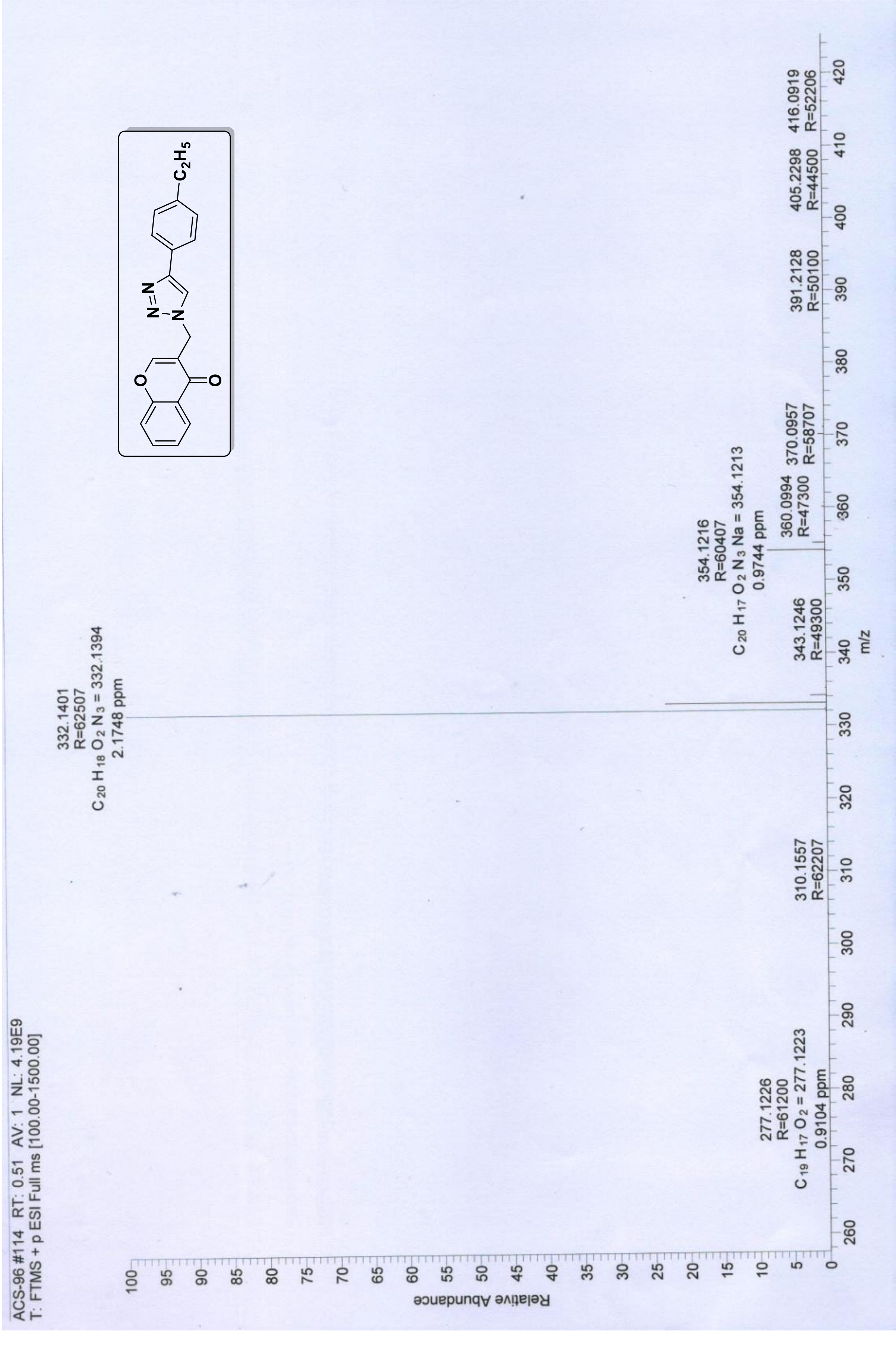

^{1}H NMR (400 MHz, $CDCl_3$) of compound ***6d***:

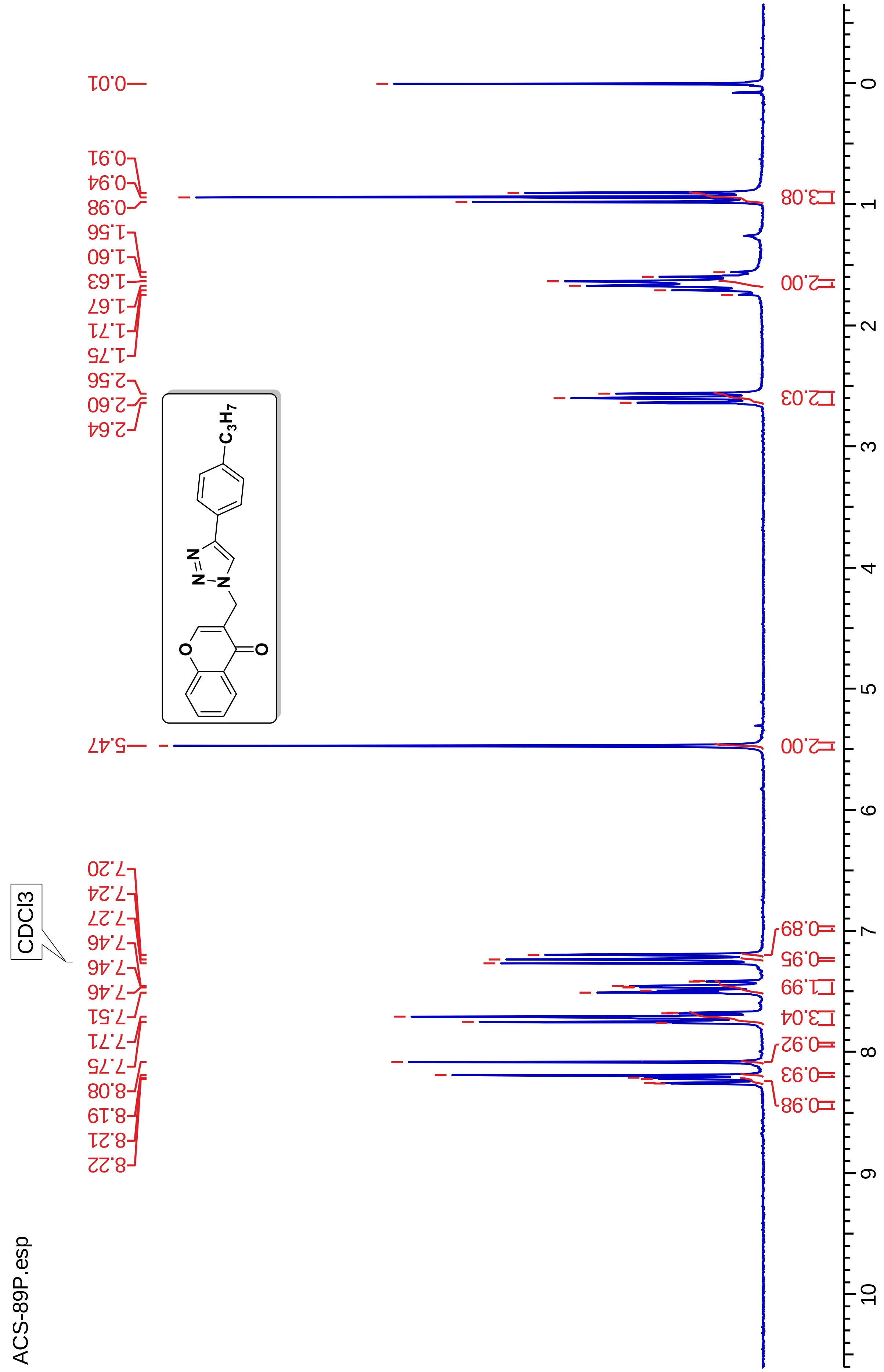

^{13}C NMR (100 MHz, $CDCl_3$) of compound ***6d***:

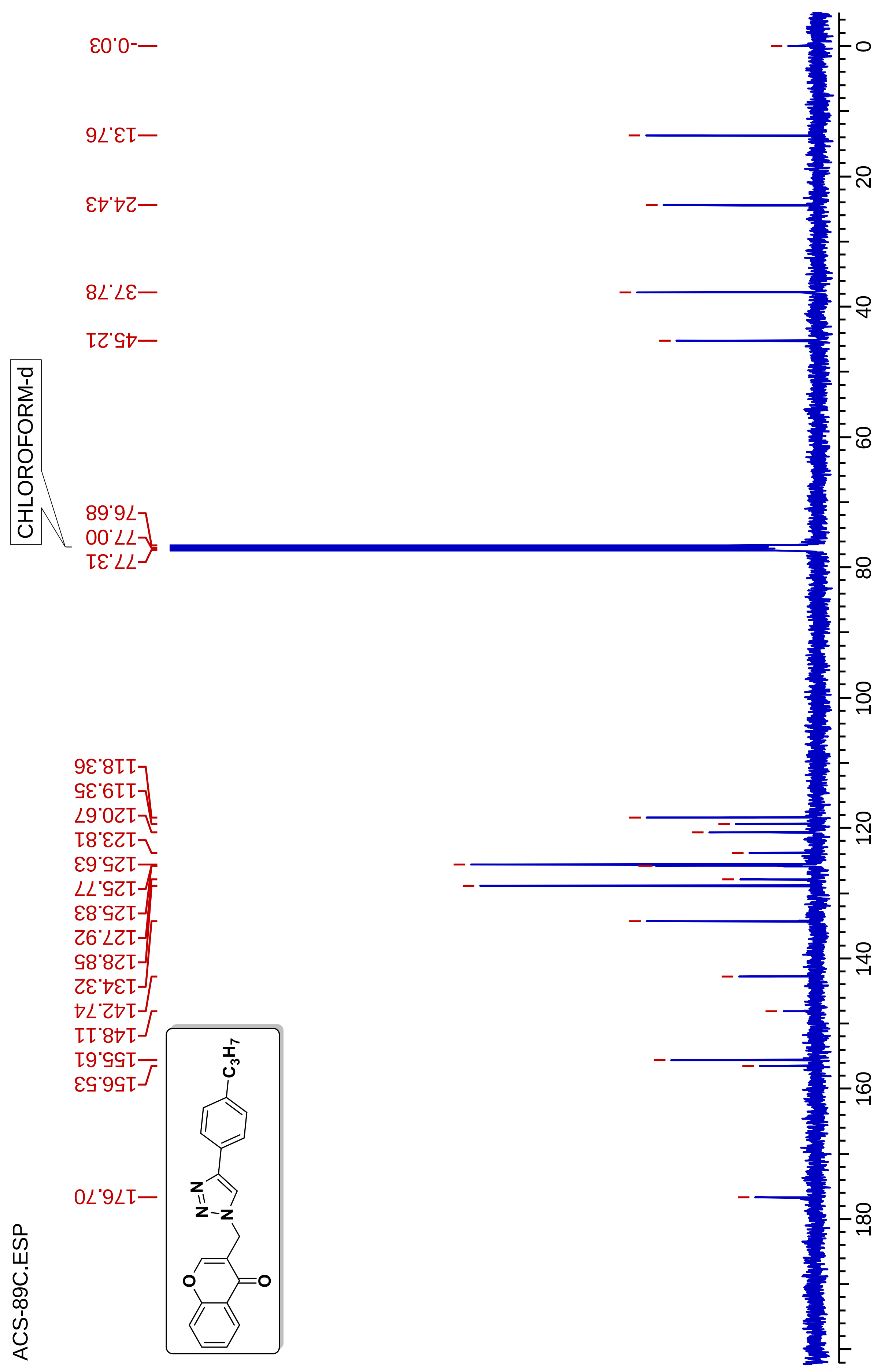

HRMS of compound ***6d***:

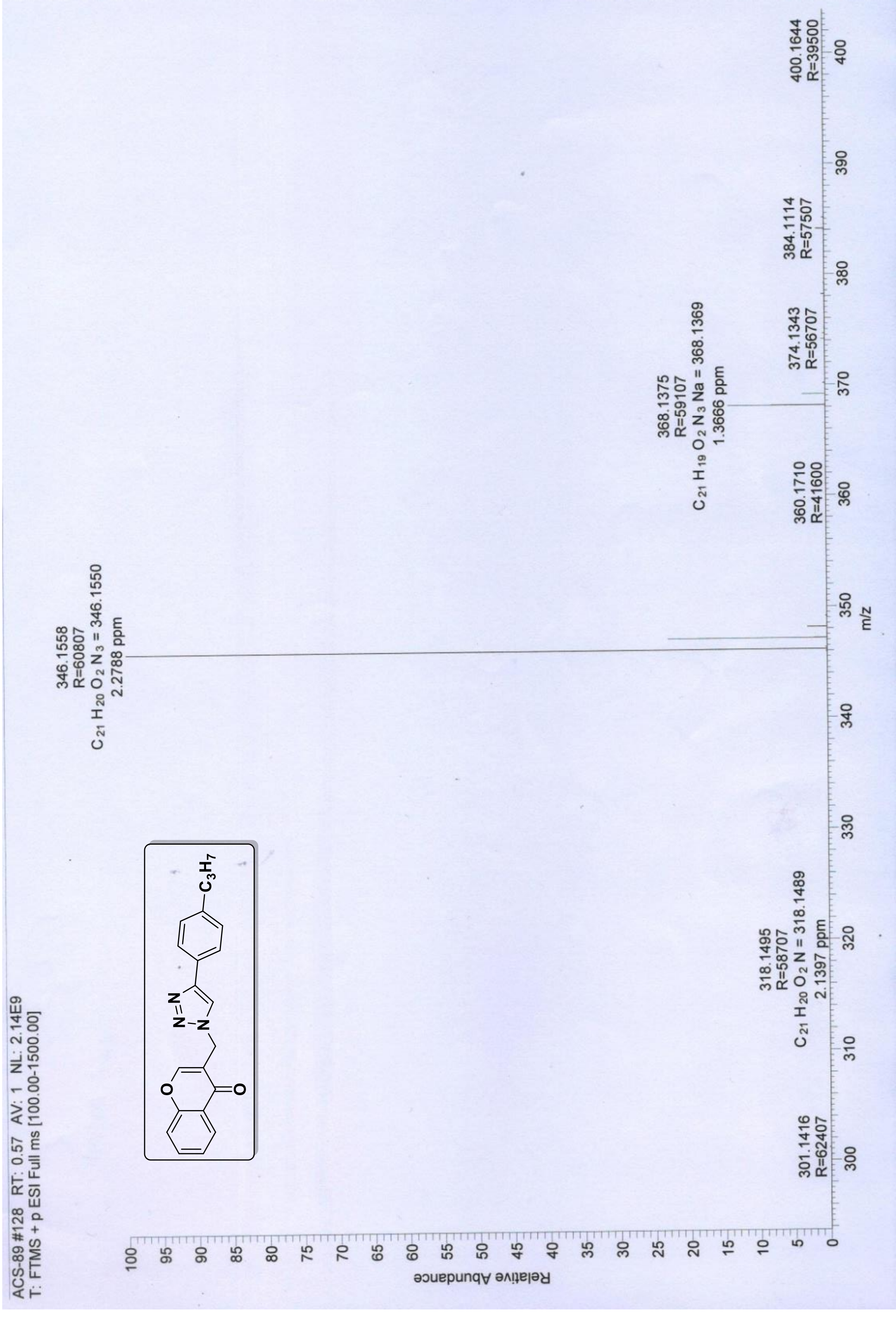

^{1}H NMR (400 MHz, $CDCl_3$) of compound ***6e***:

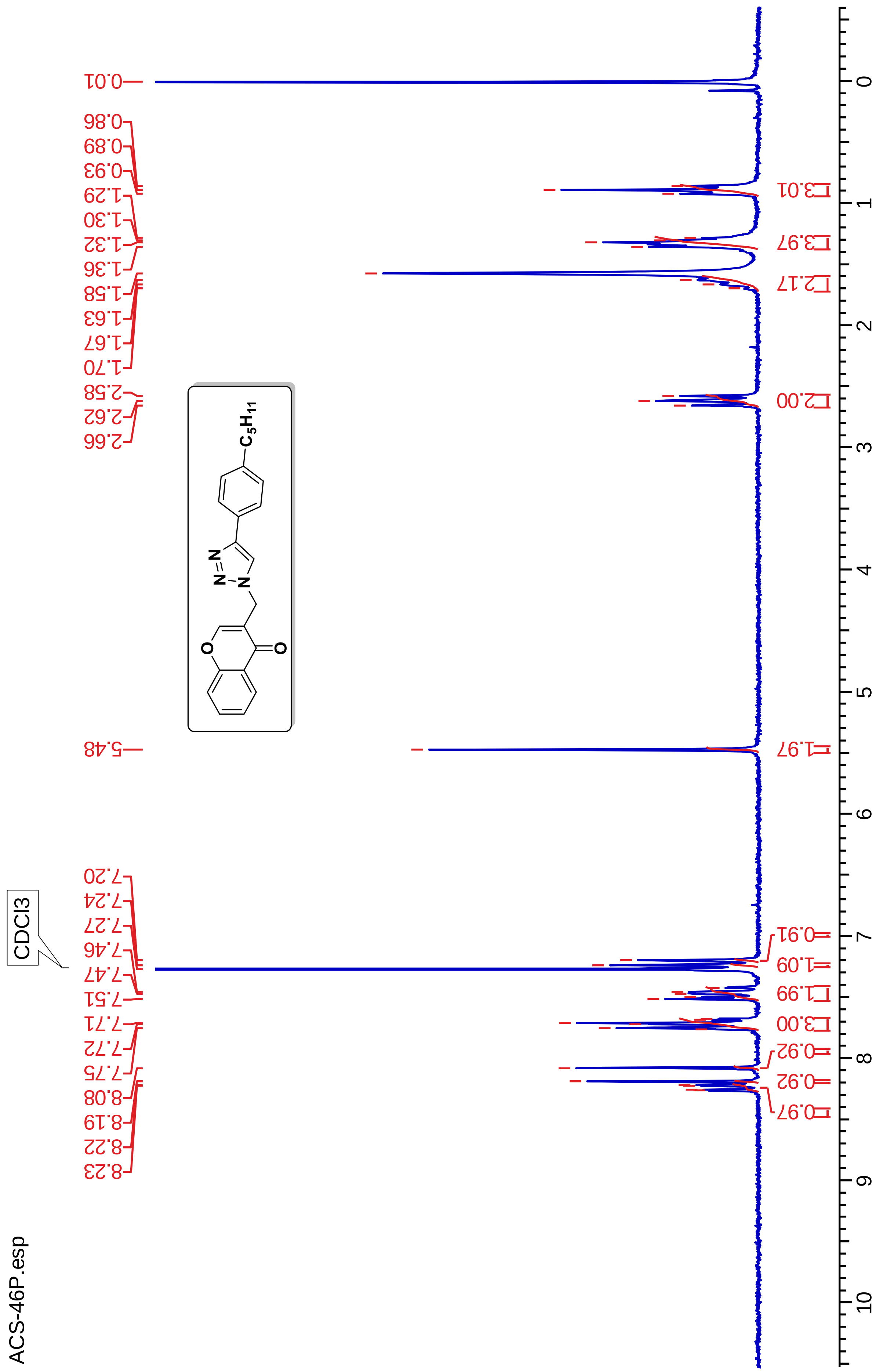

^{13}C NMR (100 MHz, $CDCl_3$) of compound ***6e***:

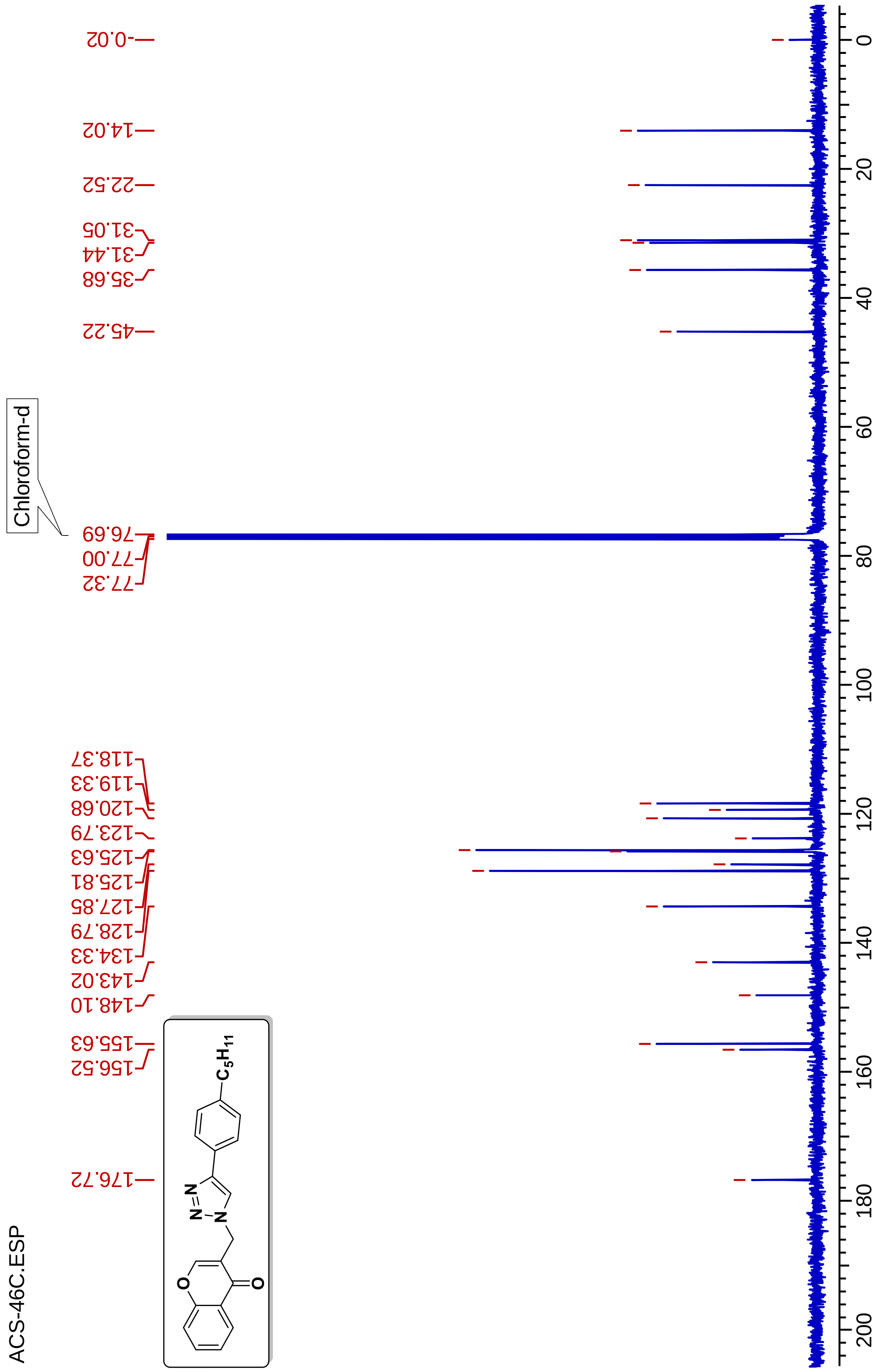

HRMS of compound ***6e***:

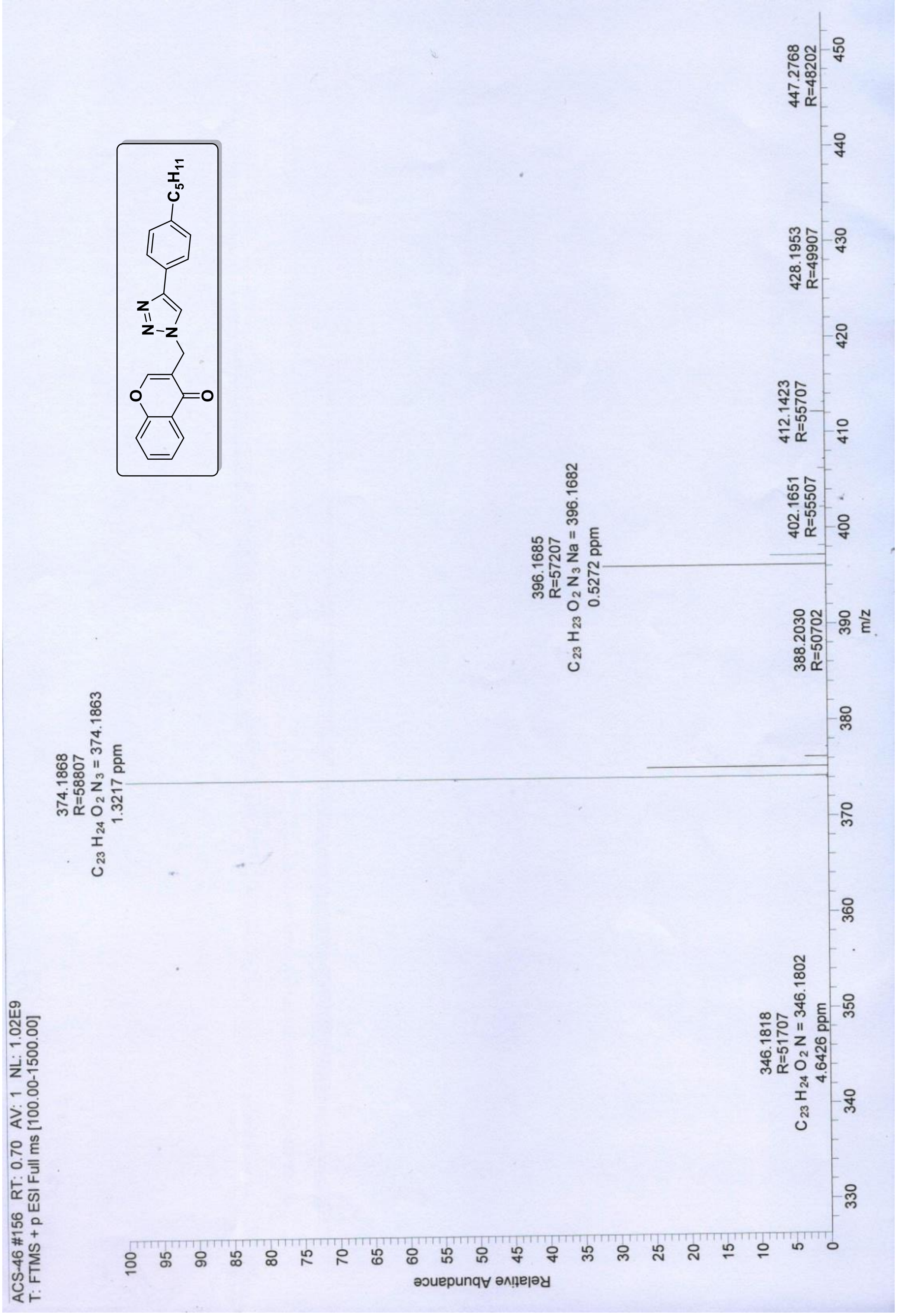

^{1}H NMR (400 MHz, $CDCl_3$) of compound ***6f***:

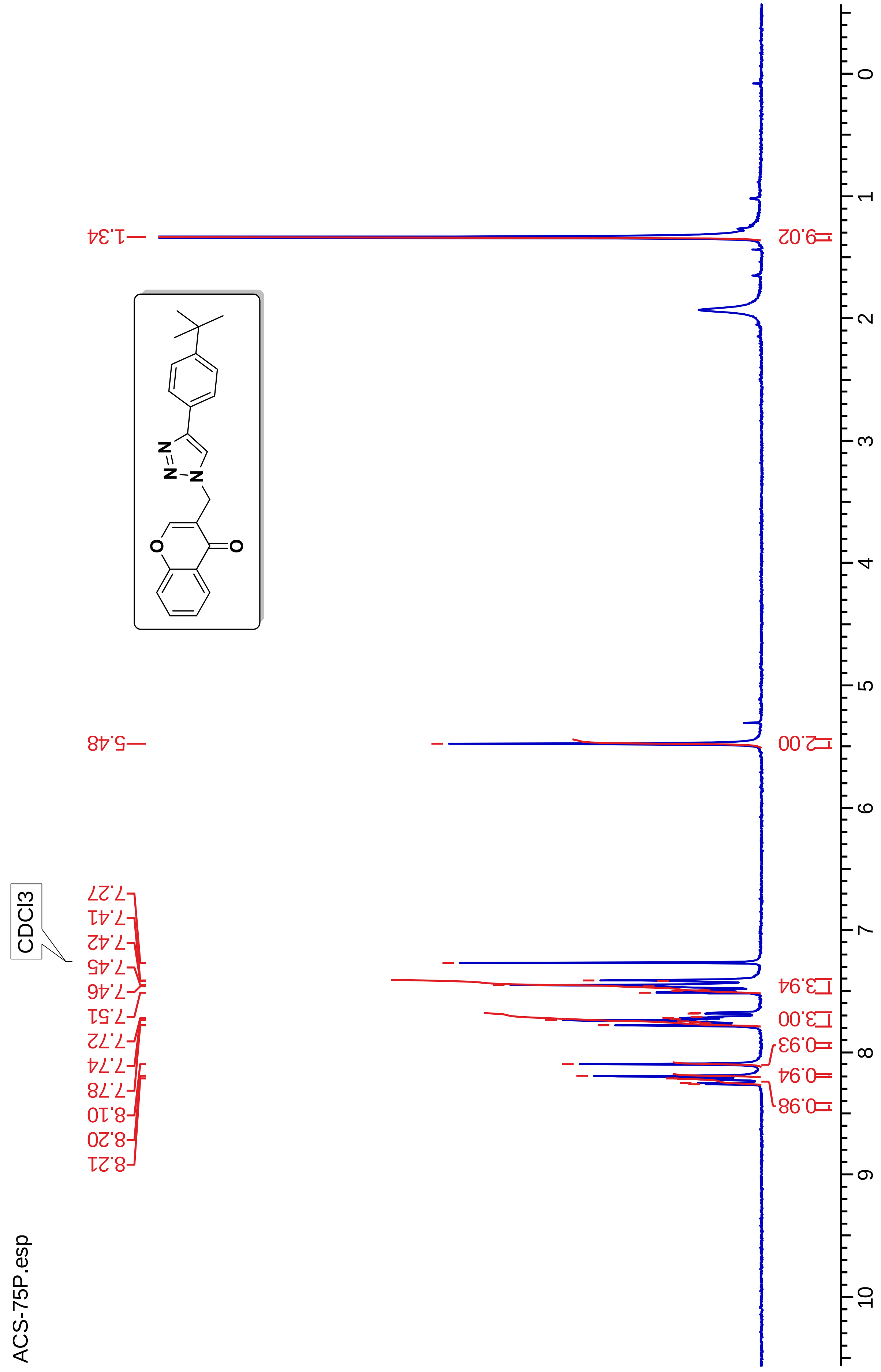

^{13}C NMR (100 MHz, $CDCl_3$) of compound ***6f***:

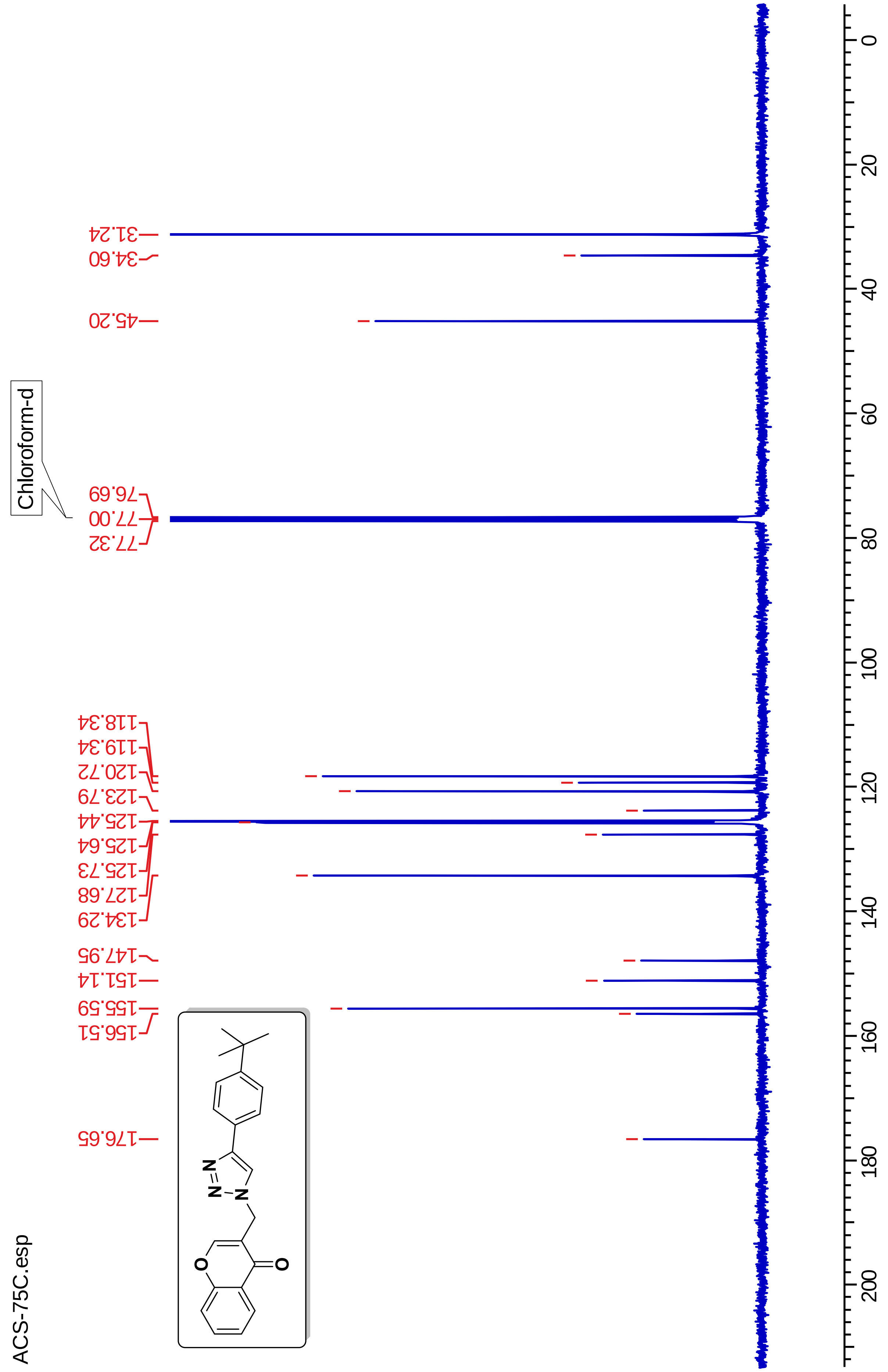

HRMS of compound ***6f***:

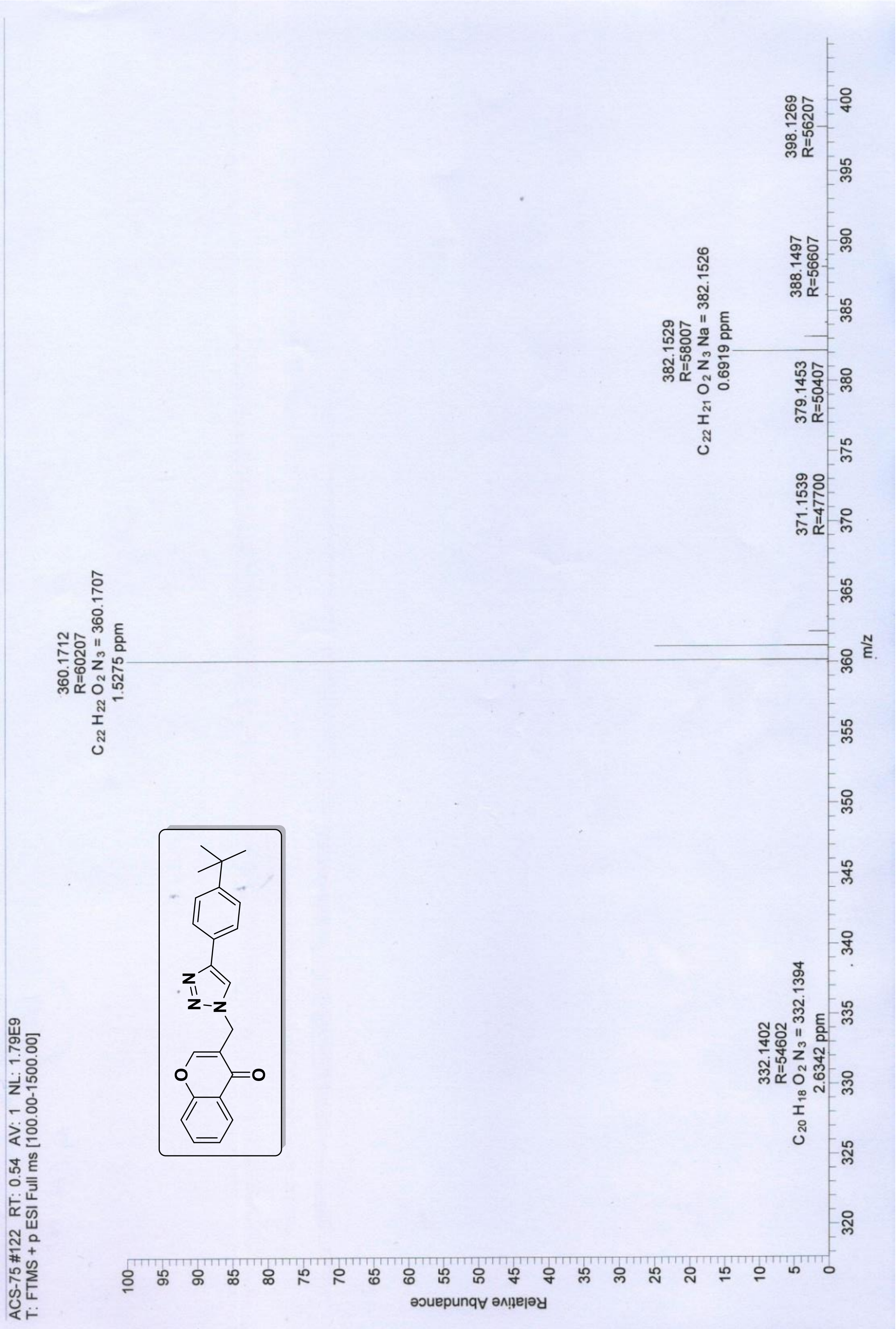

^{1}H NMR (400 MHz, $CDCl_3$) of compound ***6g***:

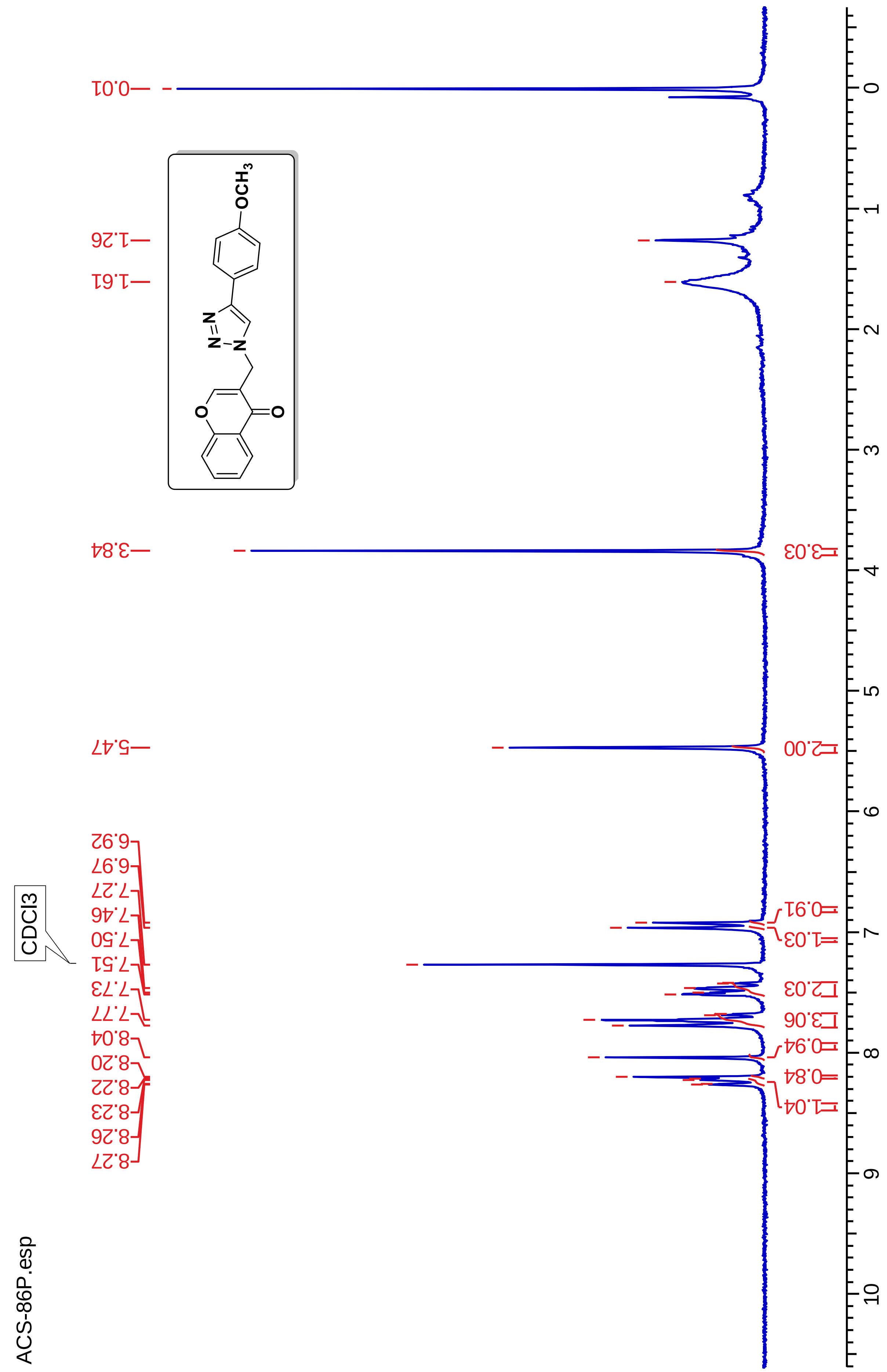

^{13}C NMR (100 MHz, CDCl$_3$) of compound ***6g***:

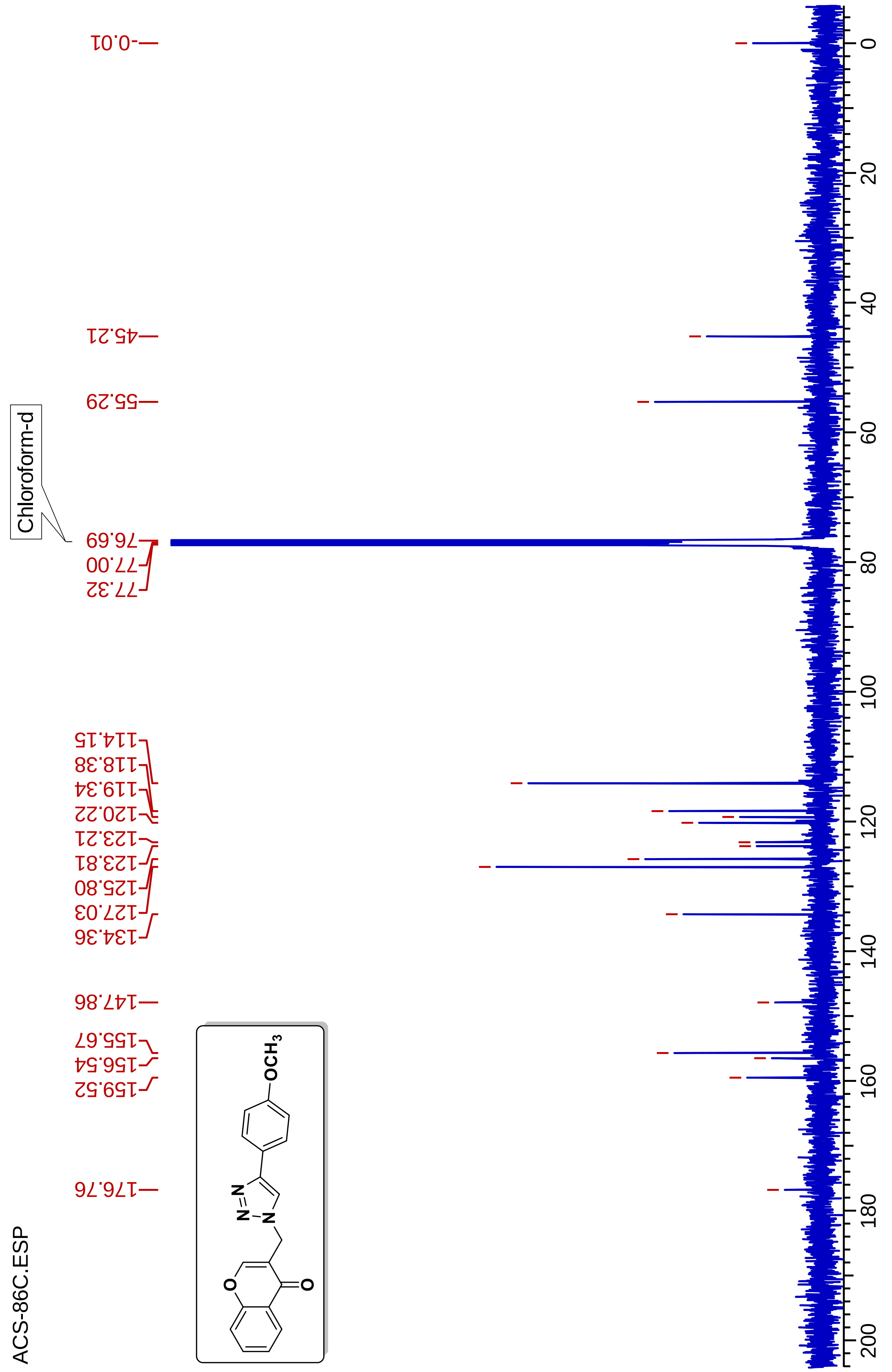

HRMS of compound ***6g***:

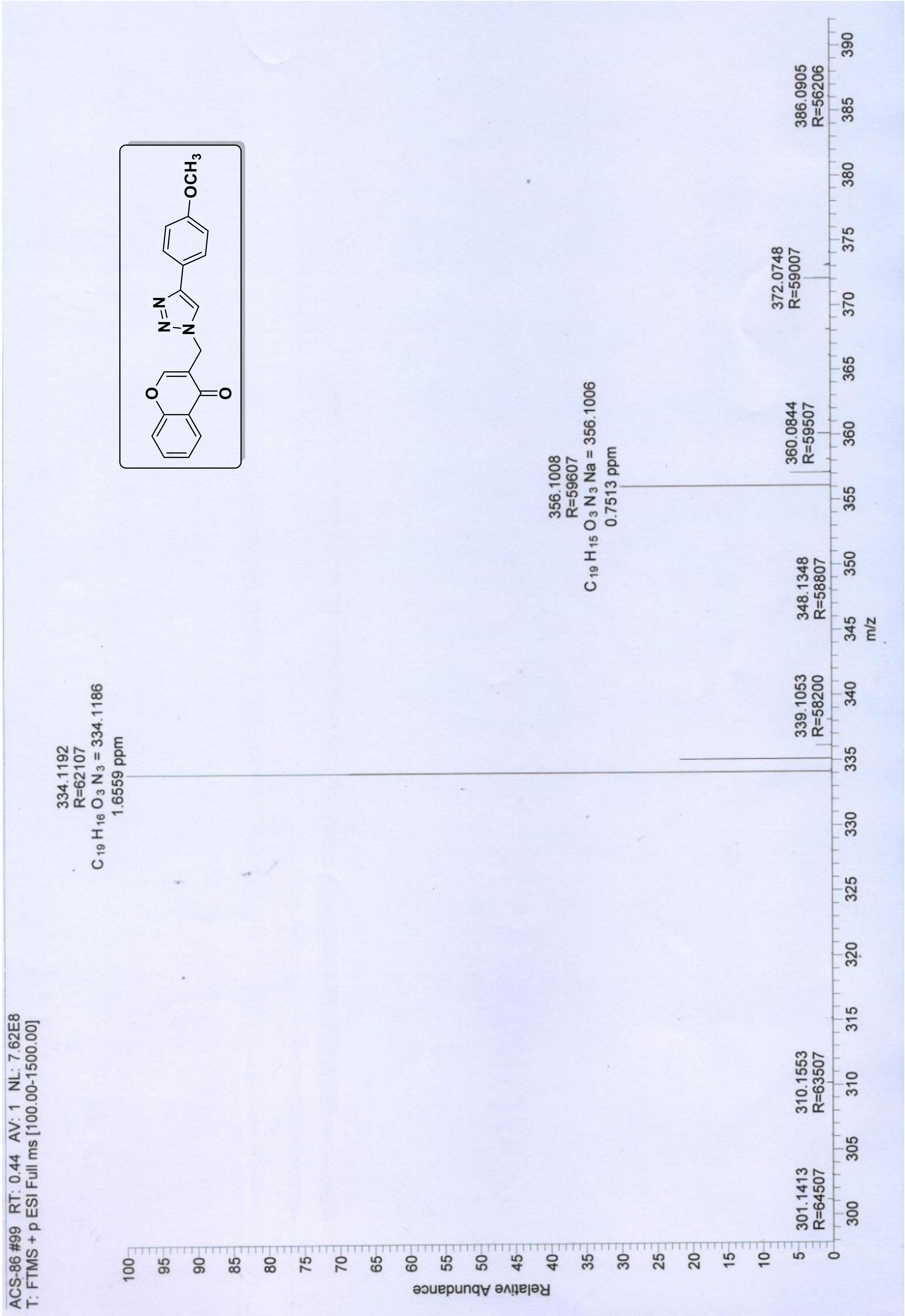

^{1}H NMR (400 MHz, $CDCl_3$) of compound ***6h***:

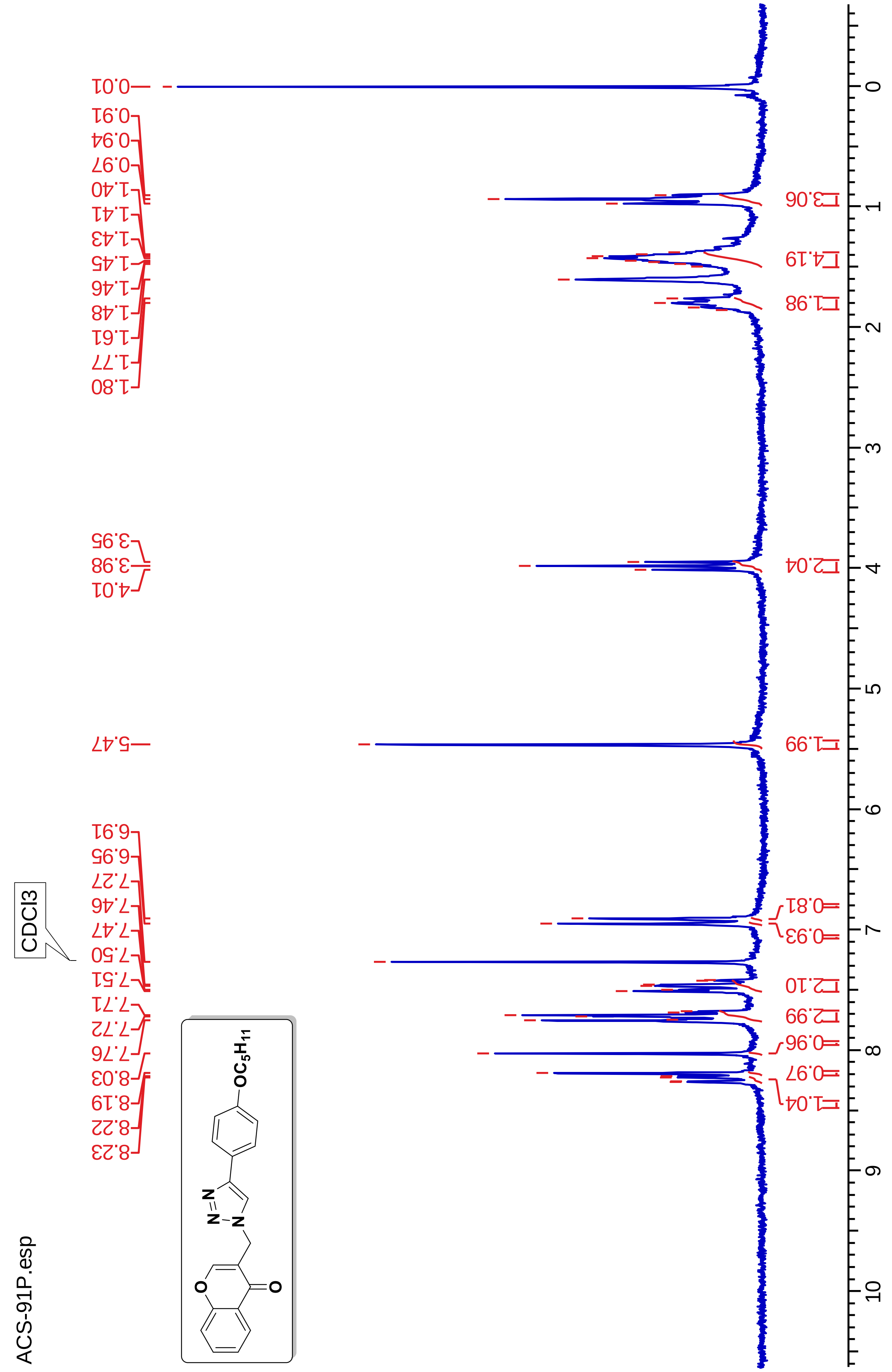

^{13}C NMR (100 MHz, $CDCl_3$) of compound ***6h***:

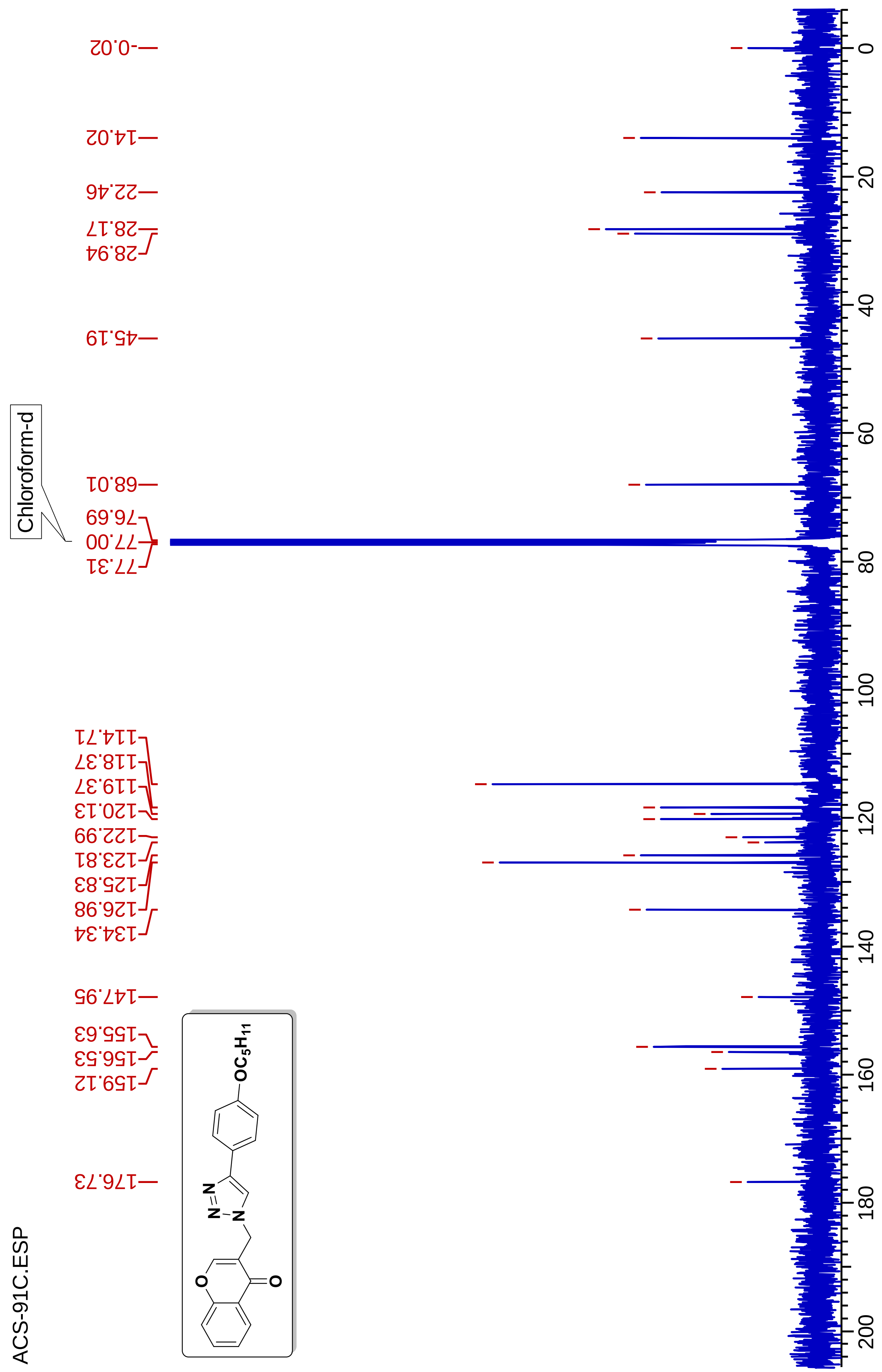

HRMS of compound ***6h***:

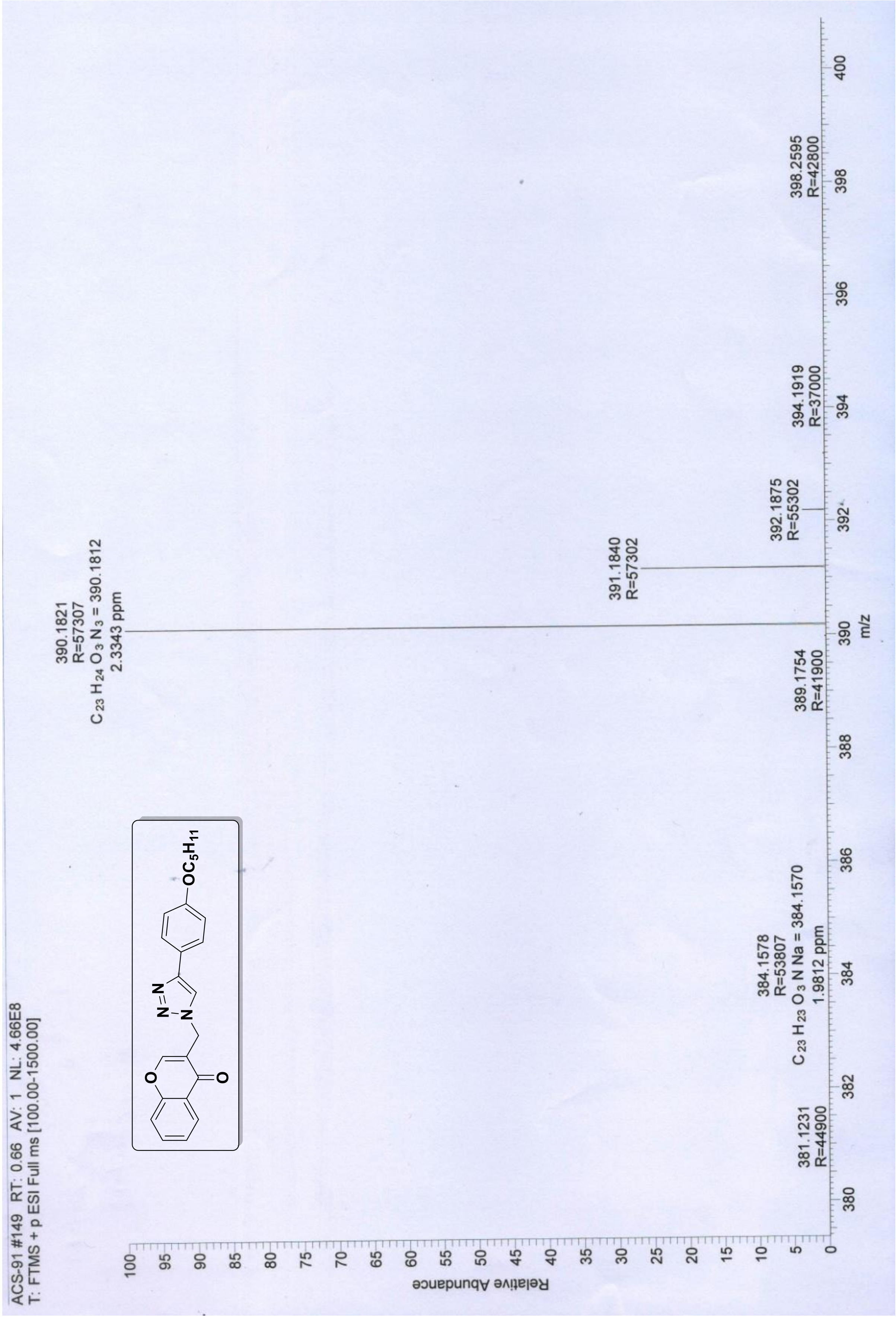

^{1}H NMR (400 MHz, $CDCl_3$) of compound ***6i***:

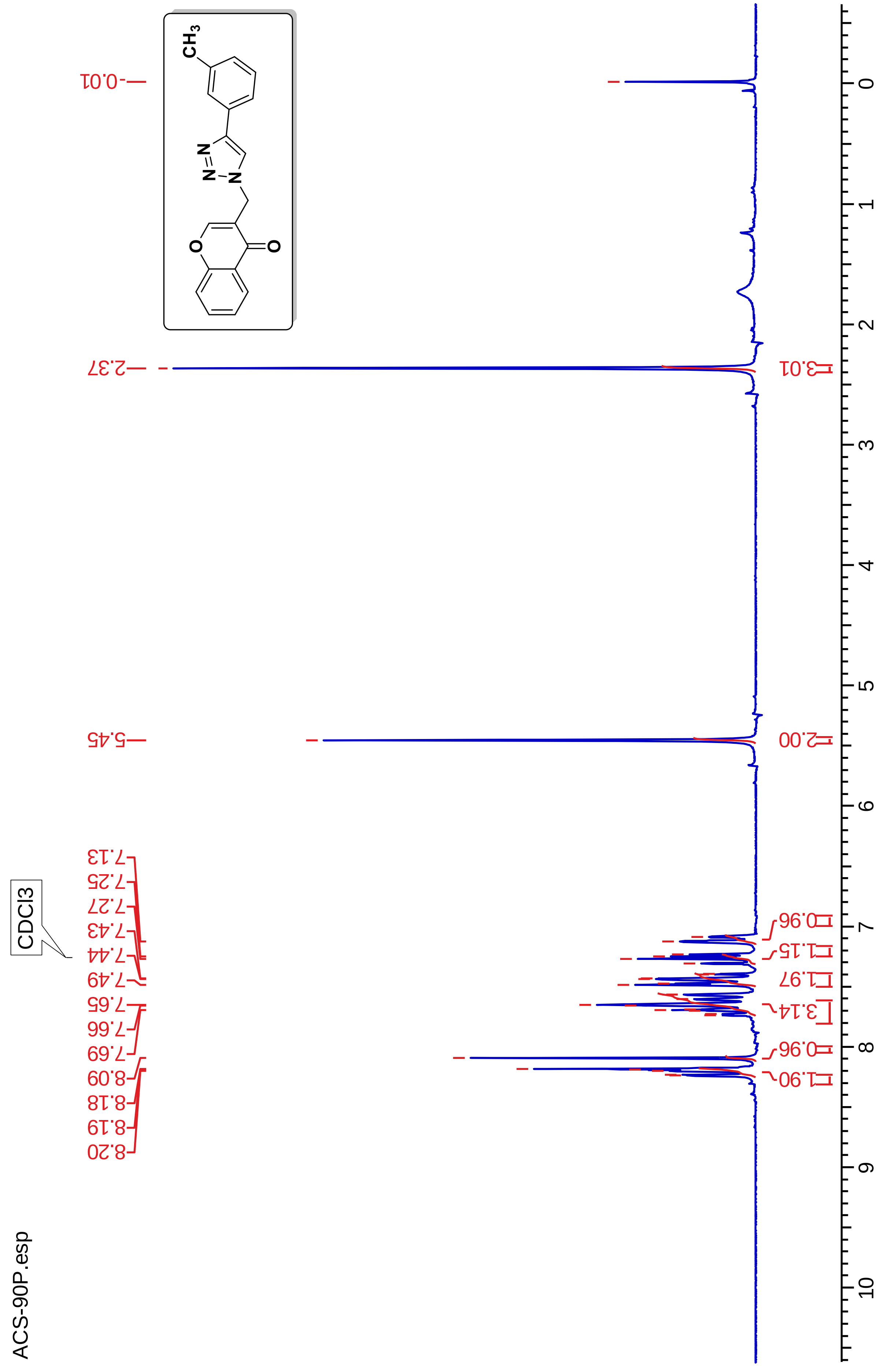

^{13}C NMR (100 MHz, $CDCl_3$) of compound ***6i***:

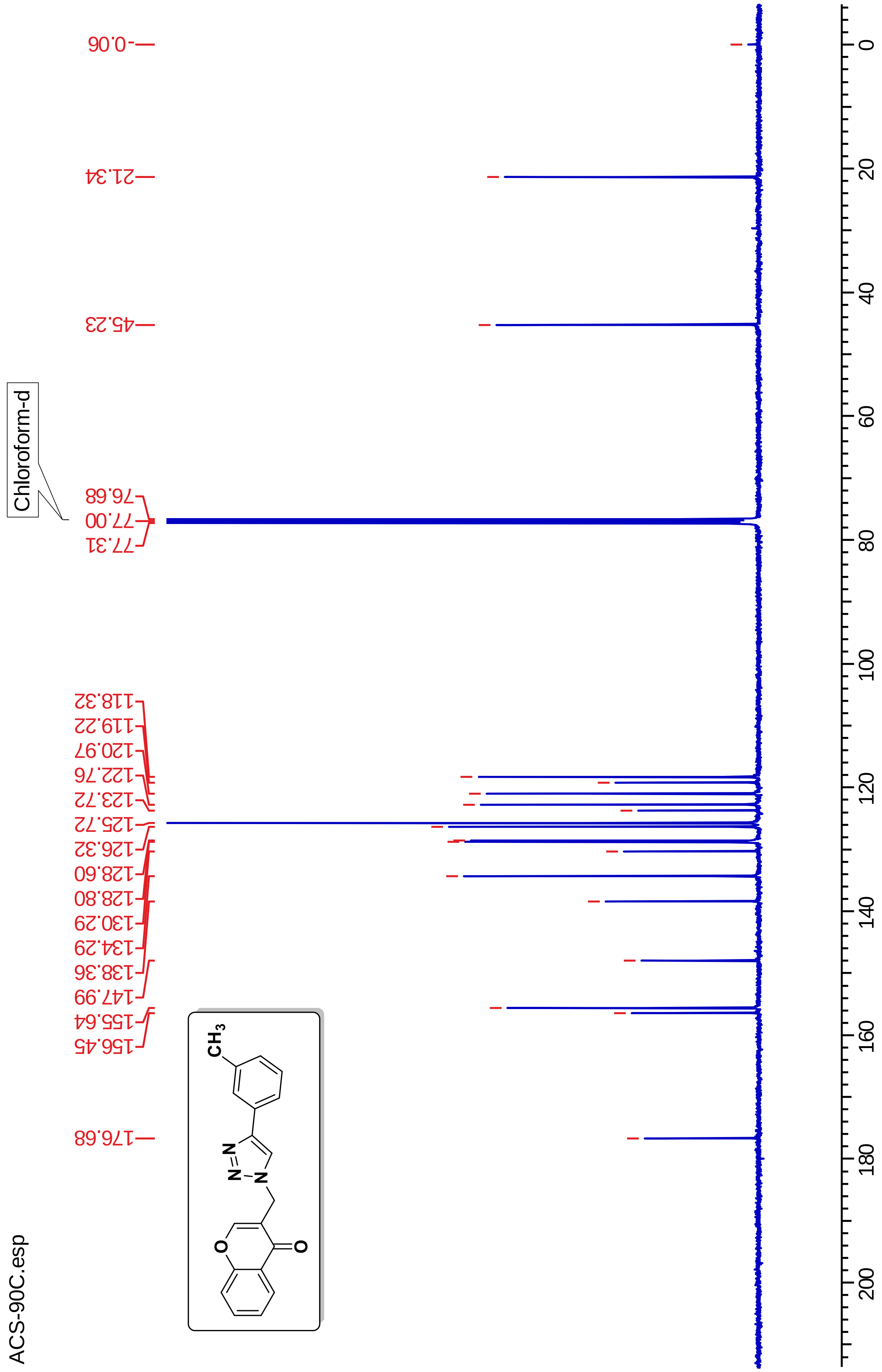

HRMS of compound ***6i***:

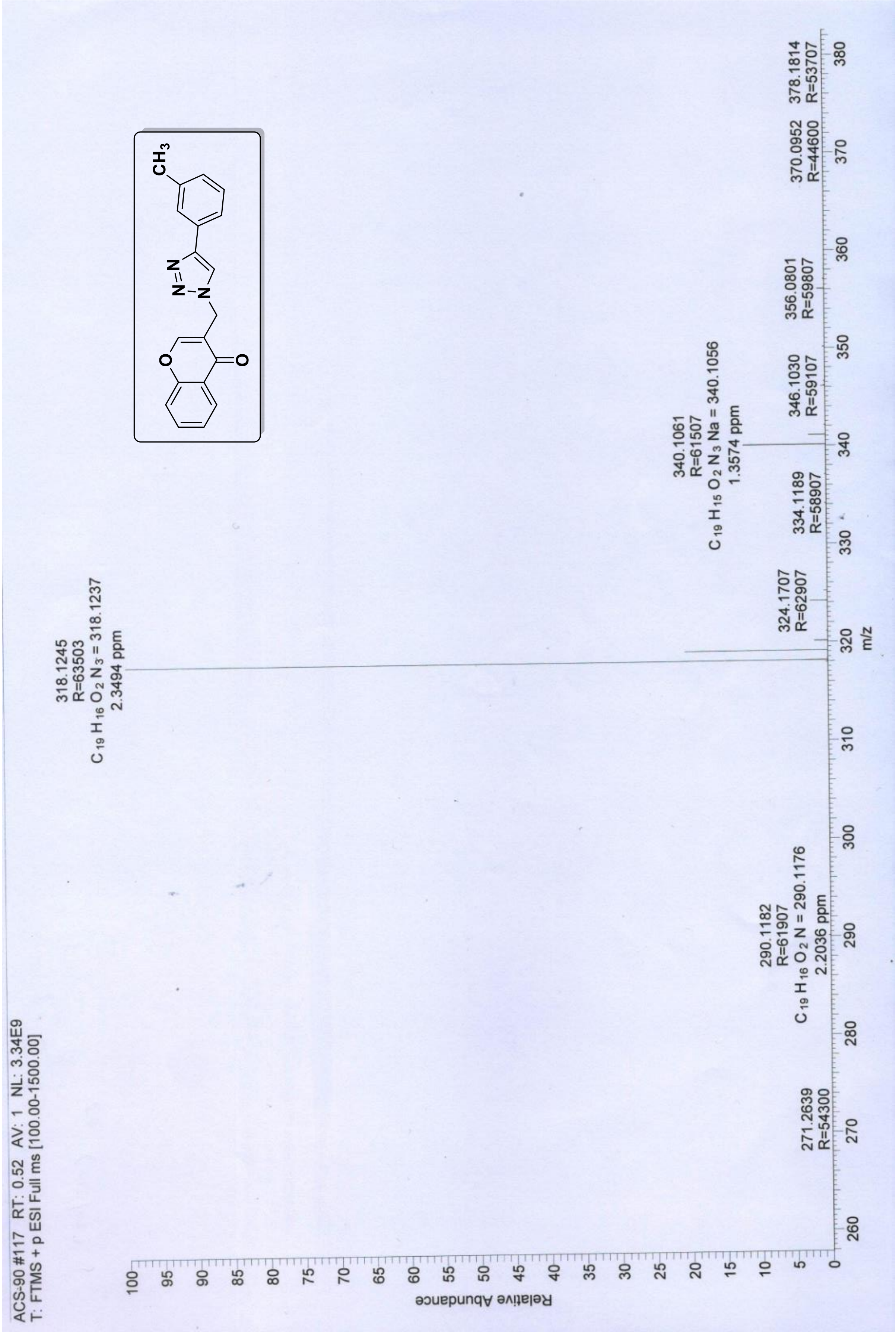

^{1}H NMR (400 MHz, $CDCl_3$) of compound ***6j***:

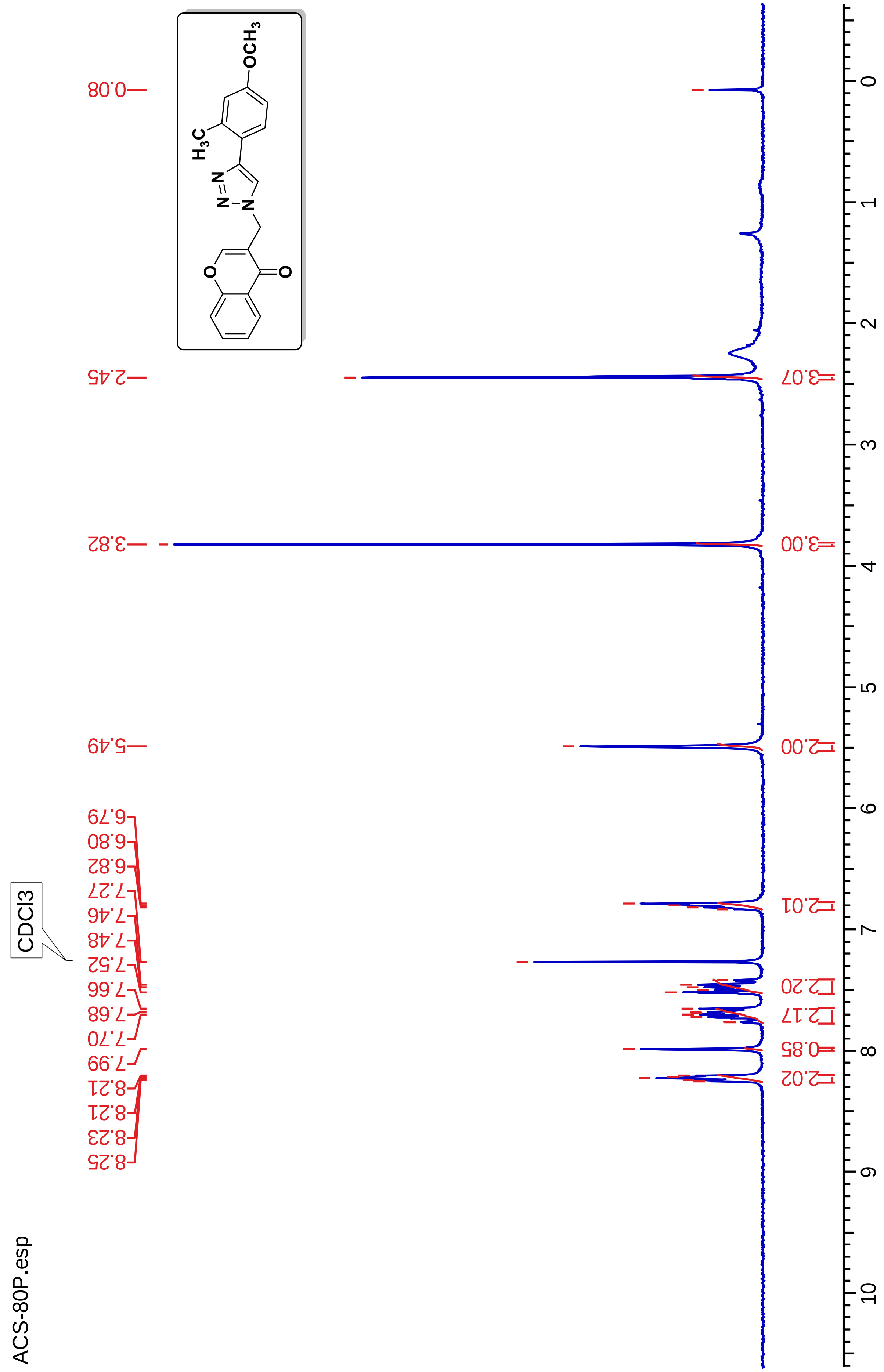

^{13}C NMR (100 MHz, $CDCl_3$) of compound ***6j***:

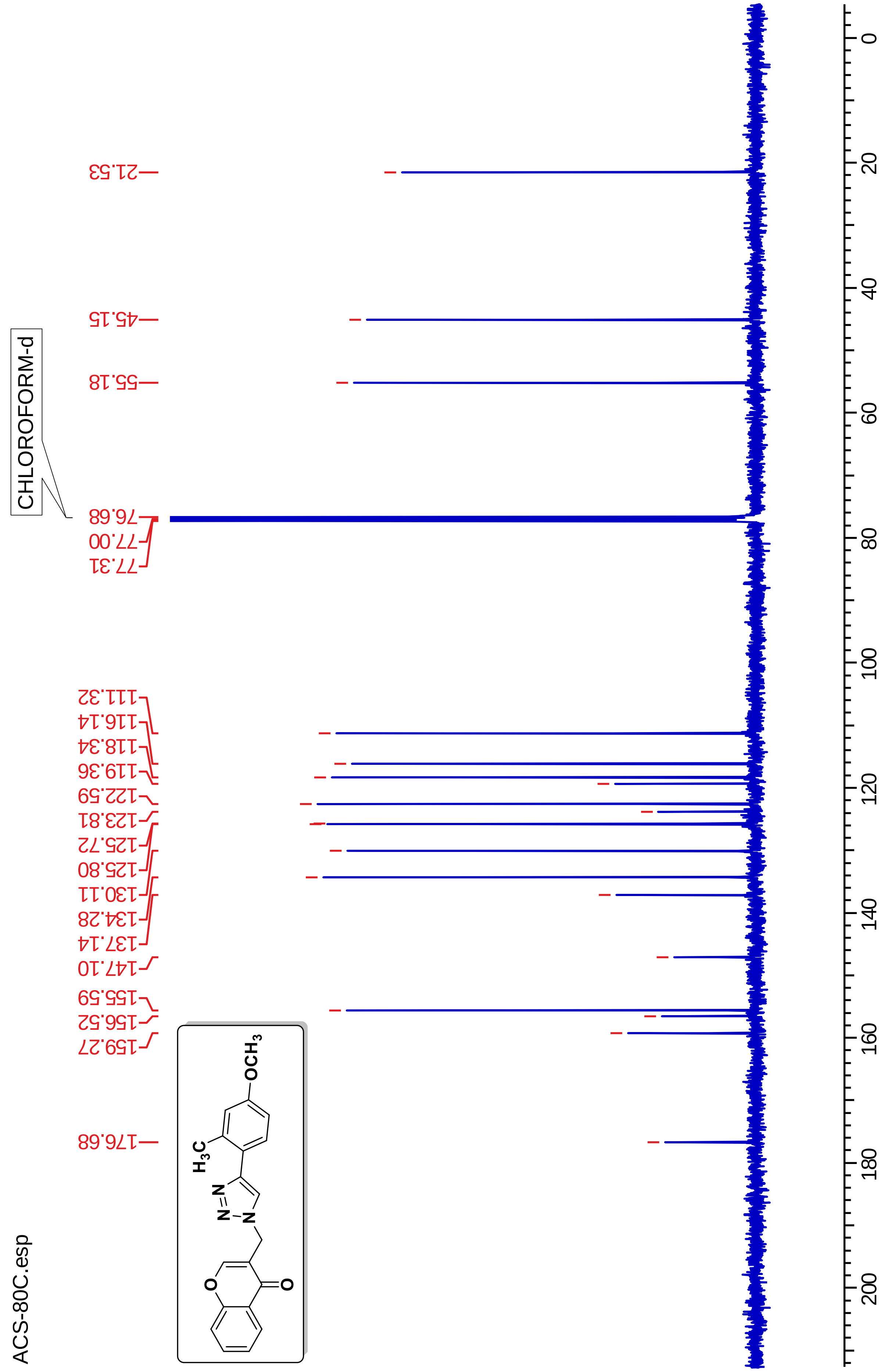

HRMS of compound ***6j***:

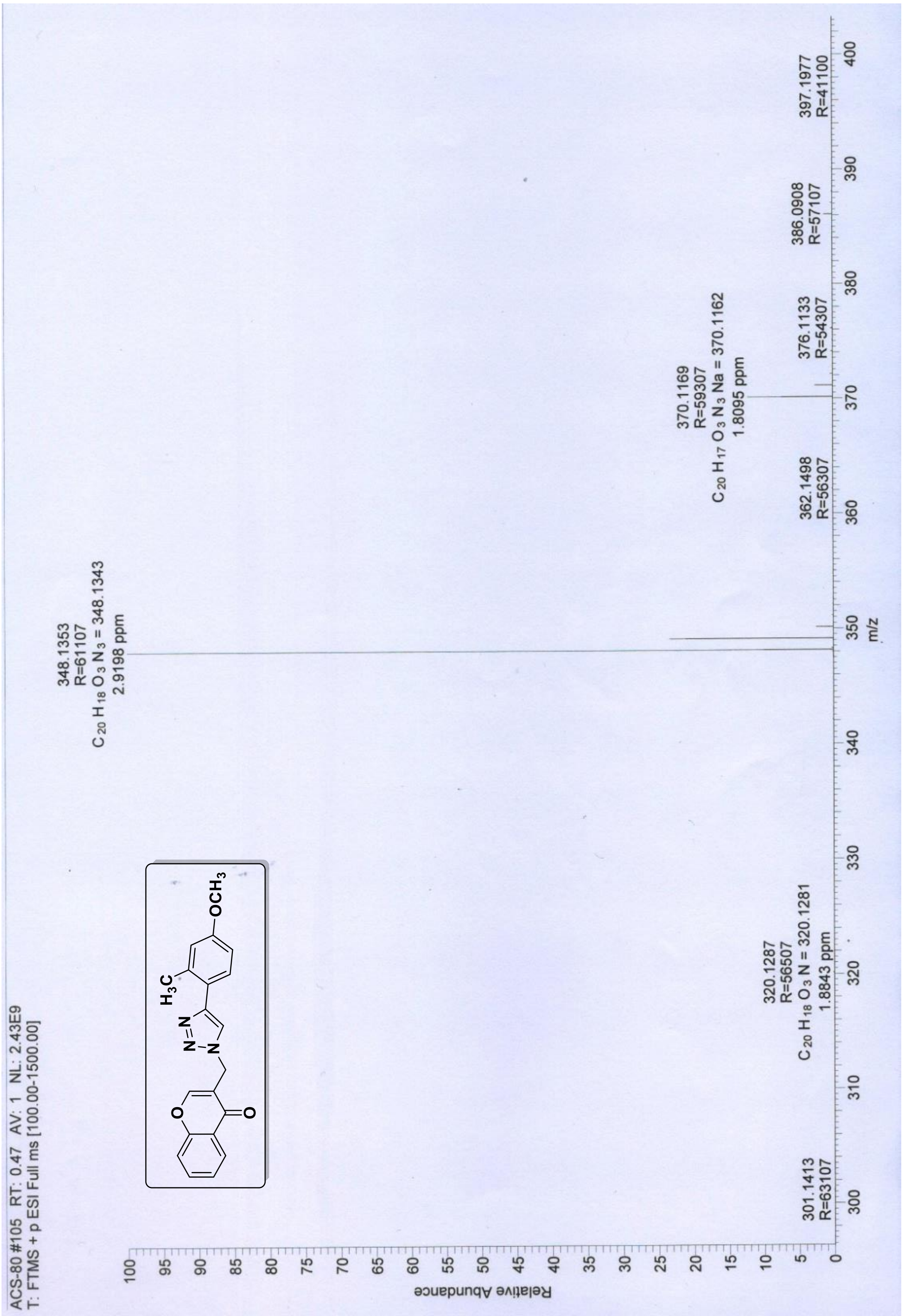

^{1}H NMR (400 MHz, $CDCl_3$) of compound ***6k***:

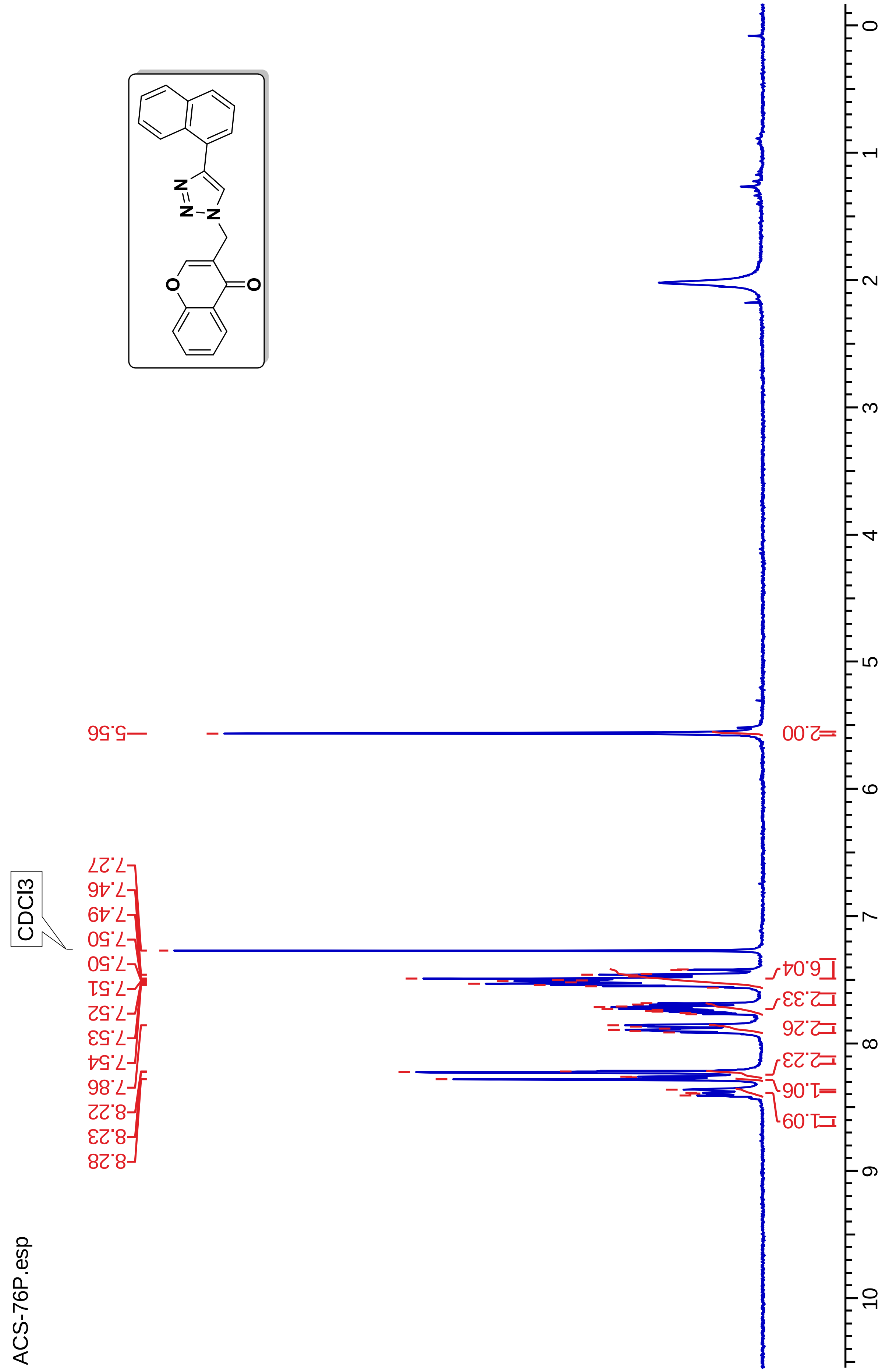

^{13}C NMR (100 MHz, $CDCl_3$) of compound ***6k***:

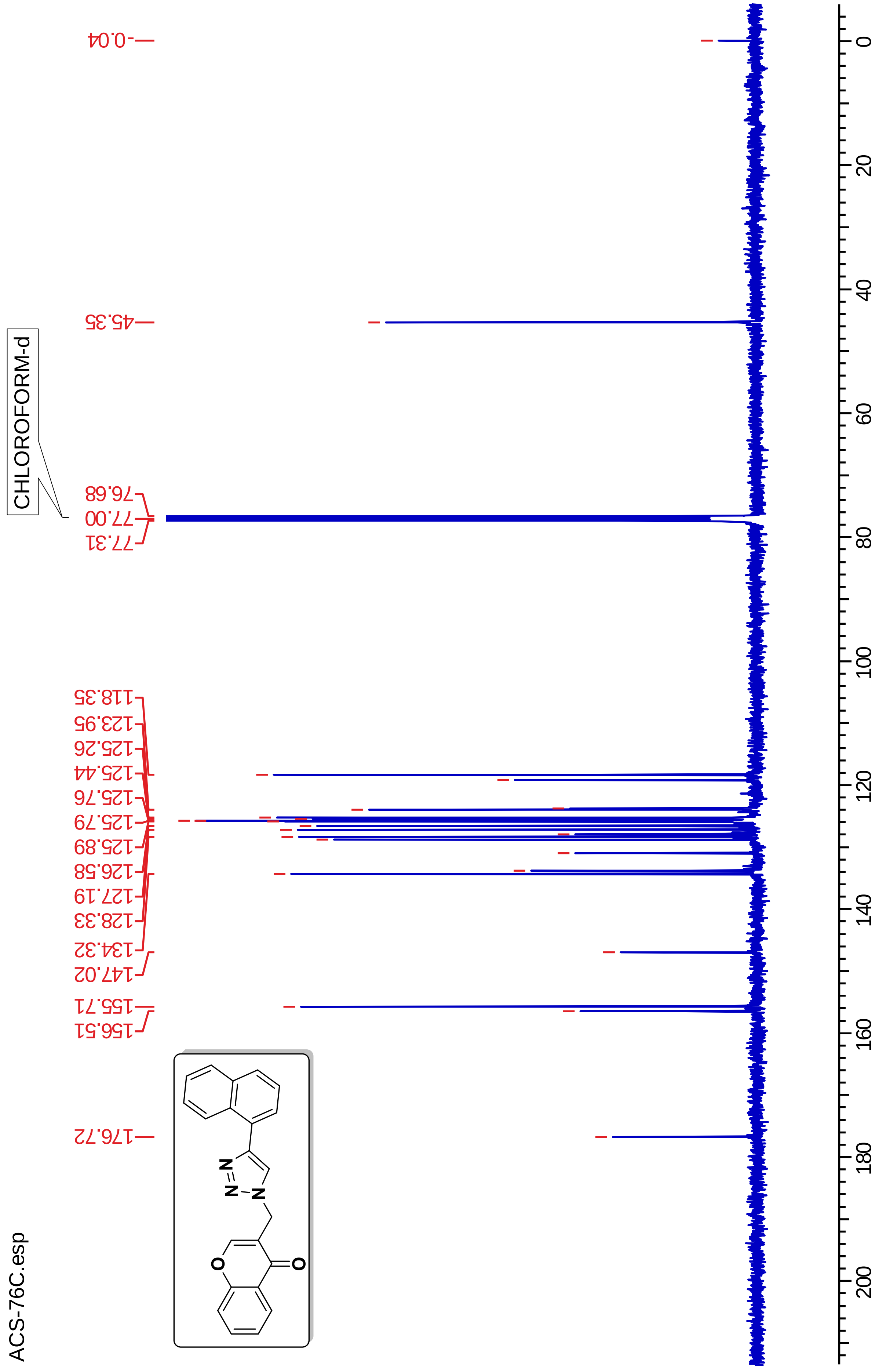

HRMS of compound ***6k***:

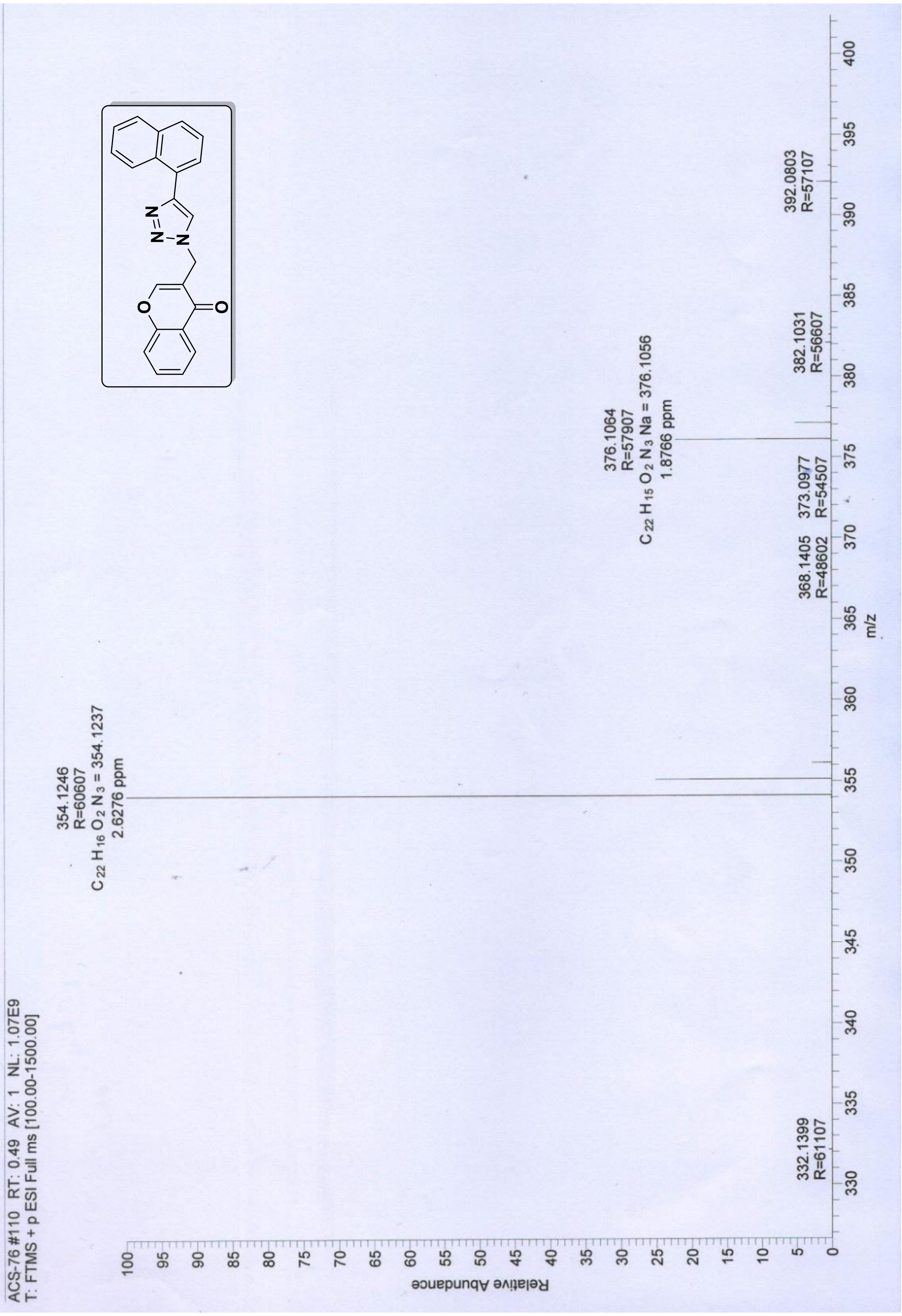

^{1}H NMR (400 MHz, $CDCl_3$) of compound ***6l***:

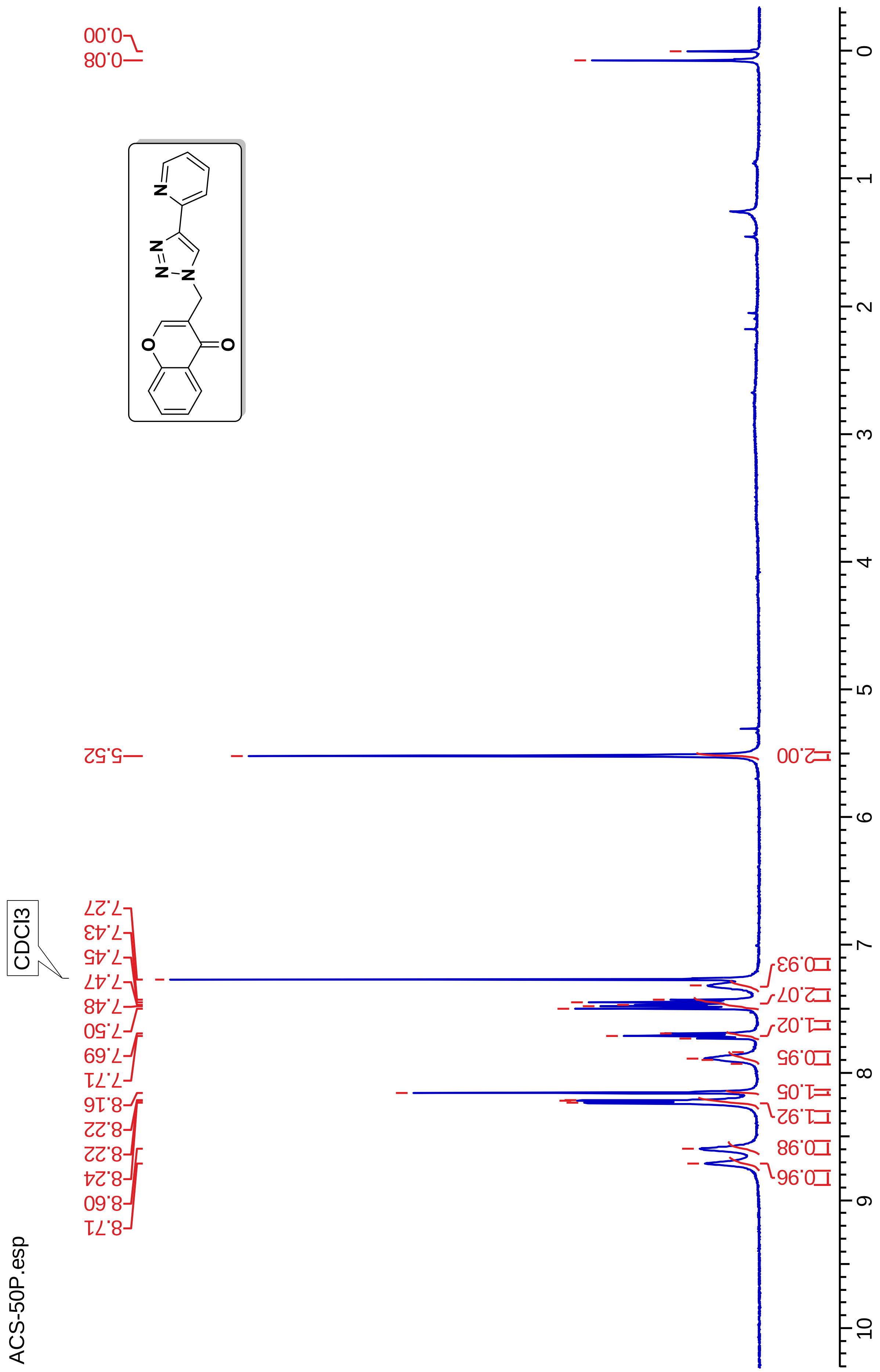

^{13}C NMR (100 MHz, $CDCl_3$) of compound ***6l***:

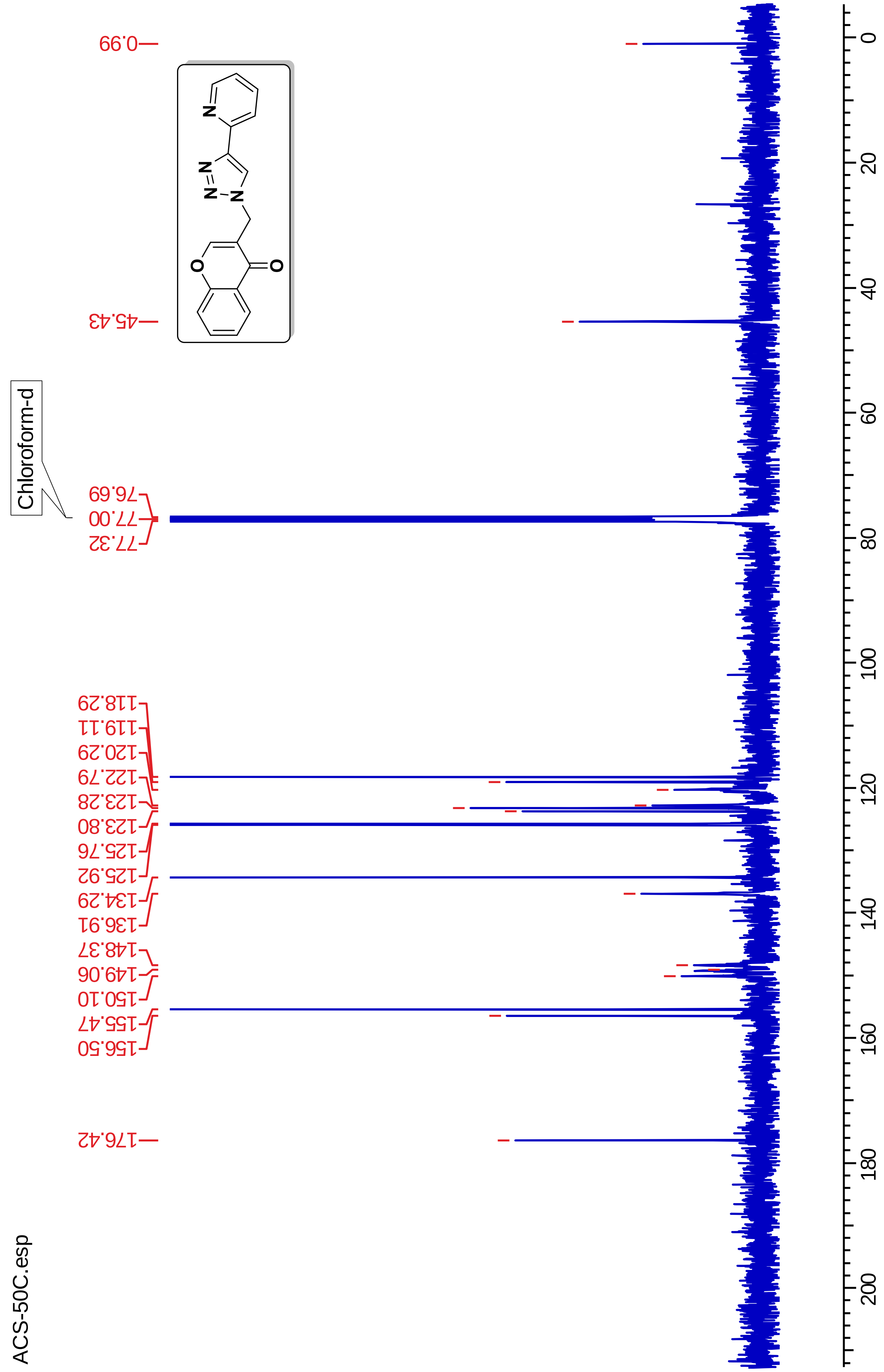

HRMS of compound ***6l***:

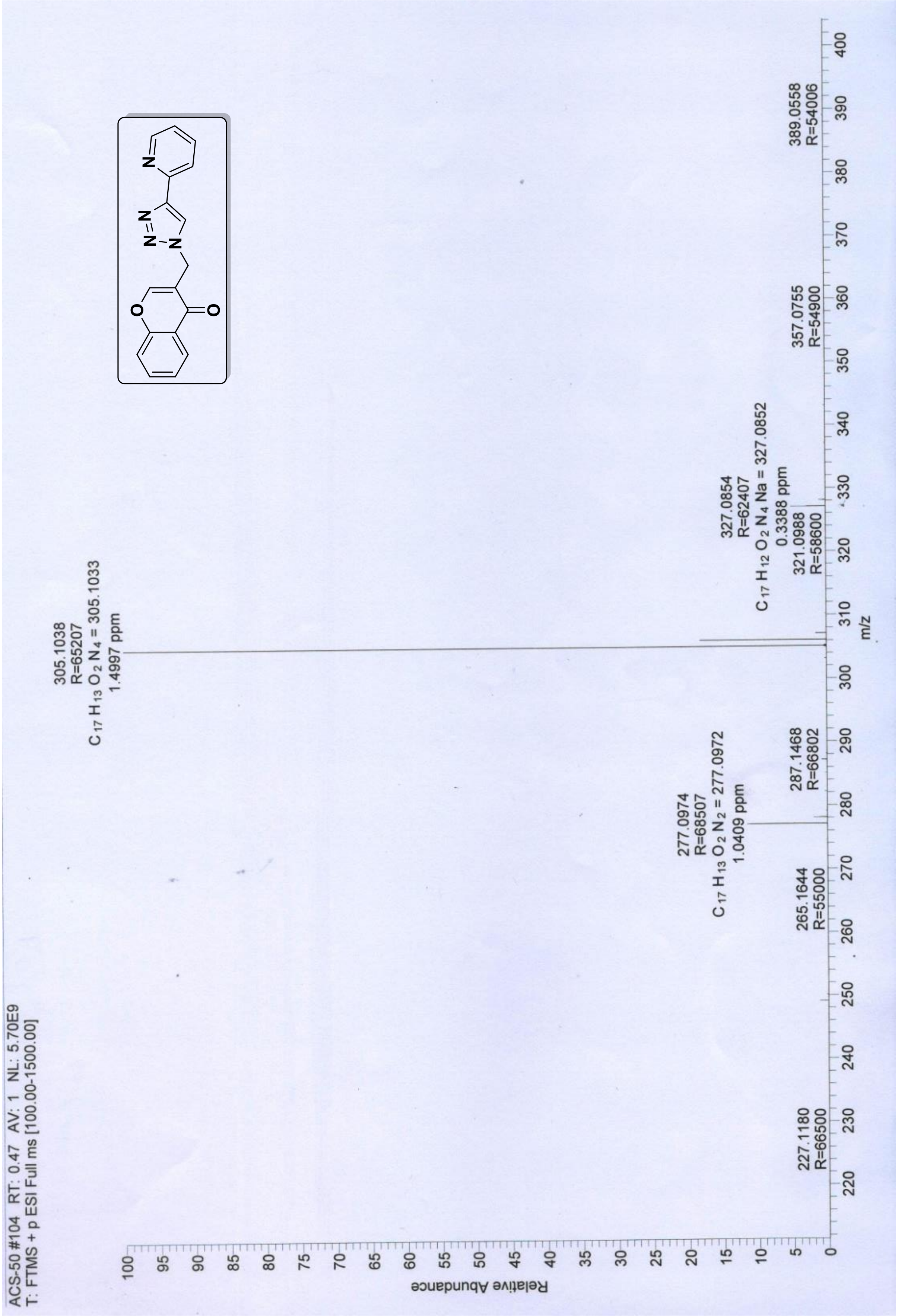

^{1}H NMR (400 MHz, $CDCl_3$) of compound ***6m***:

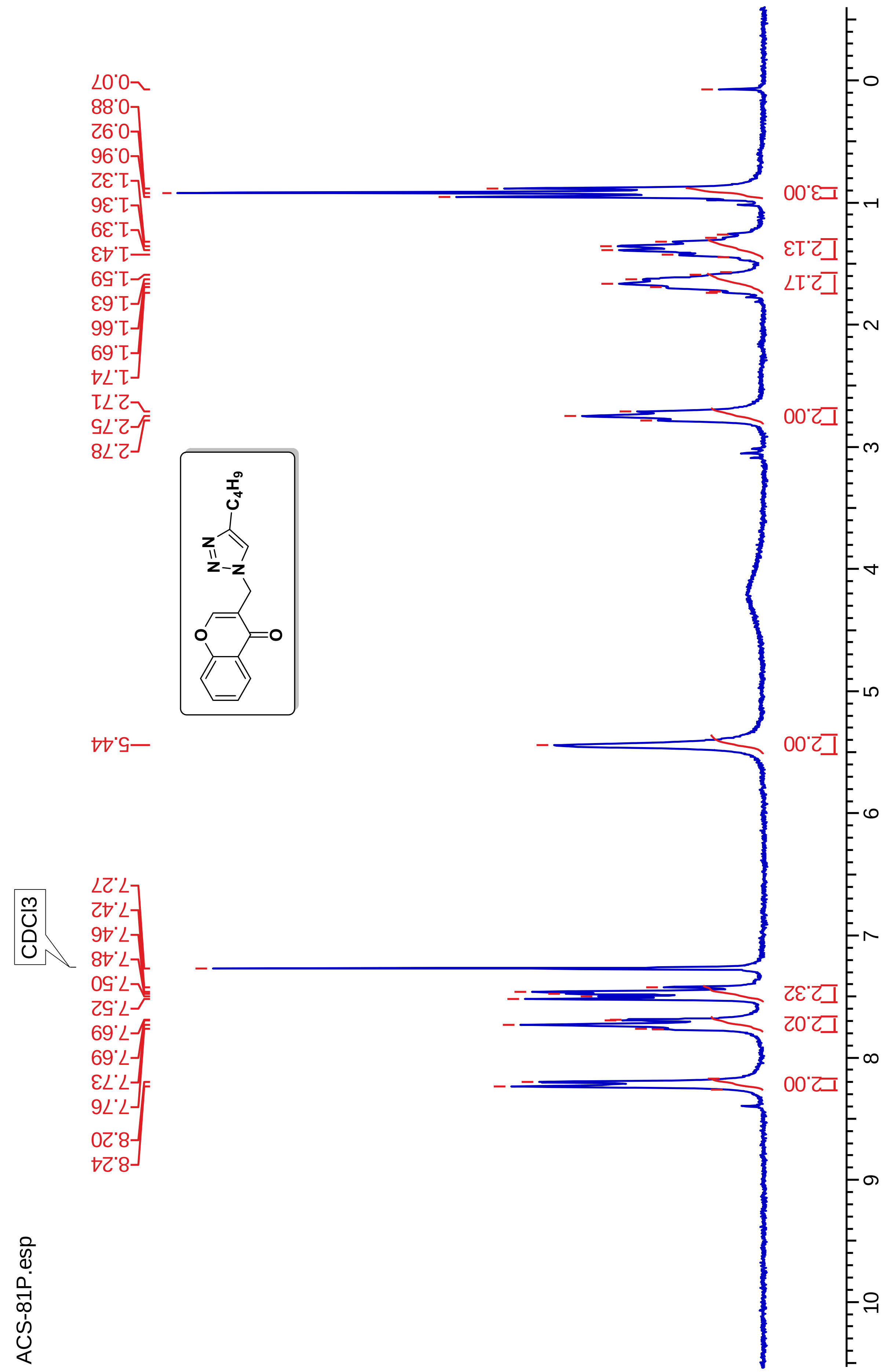

^{13}C NMR (100 MHz, $CDCl_3$) of compound ***6m***:

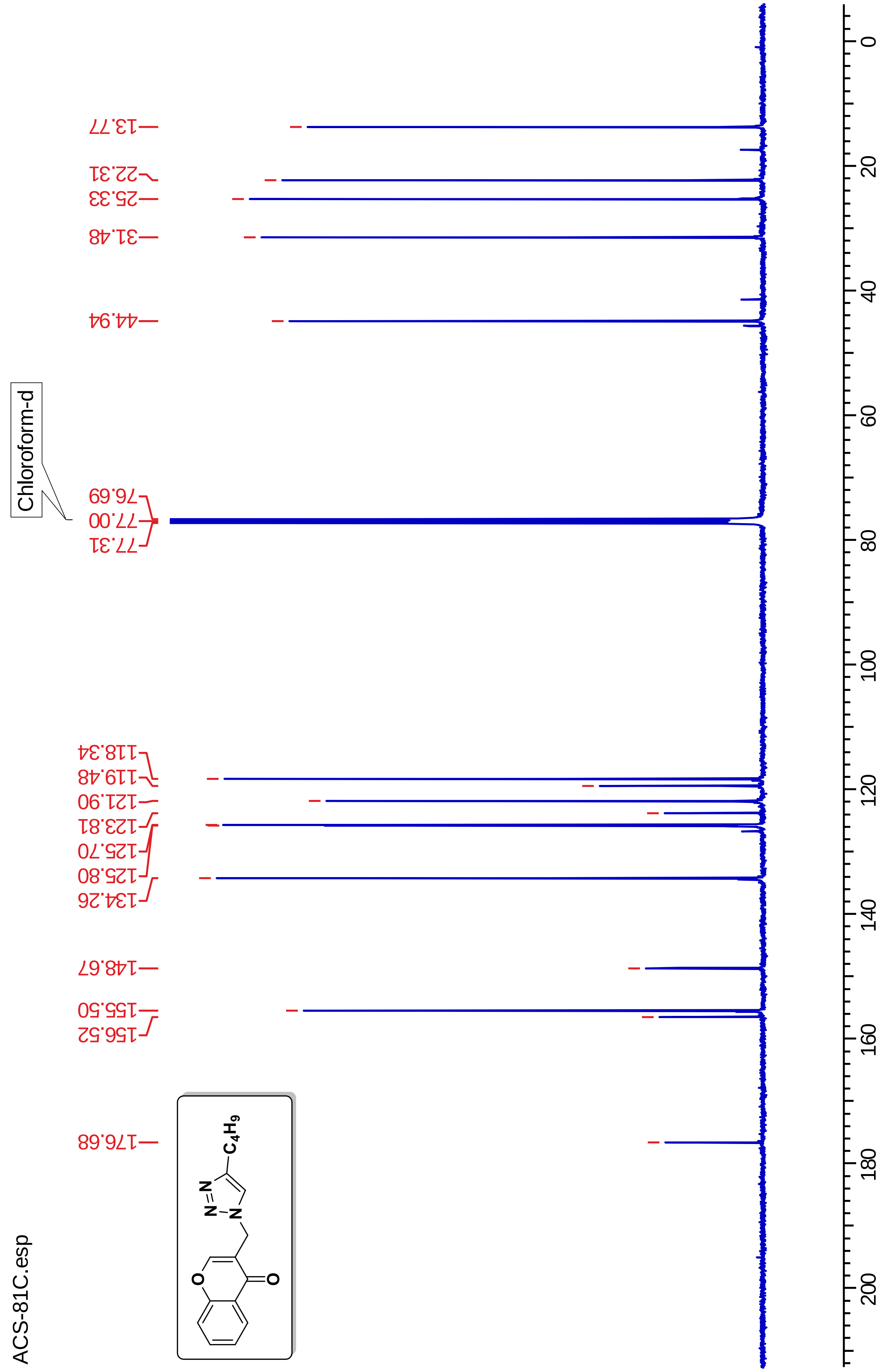

HRMS of compound ***6m***:

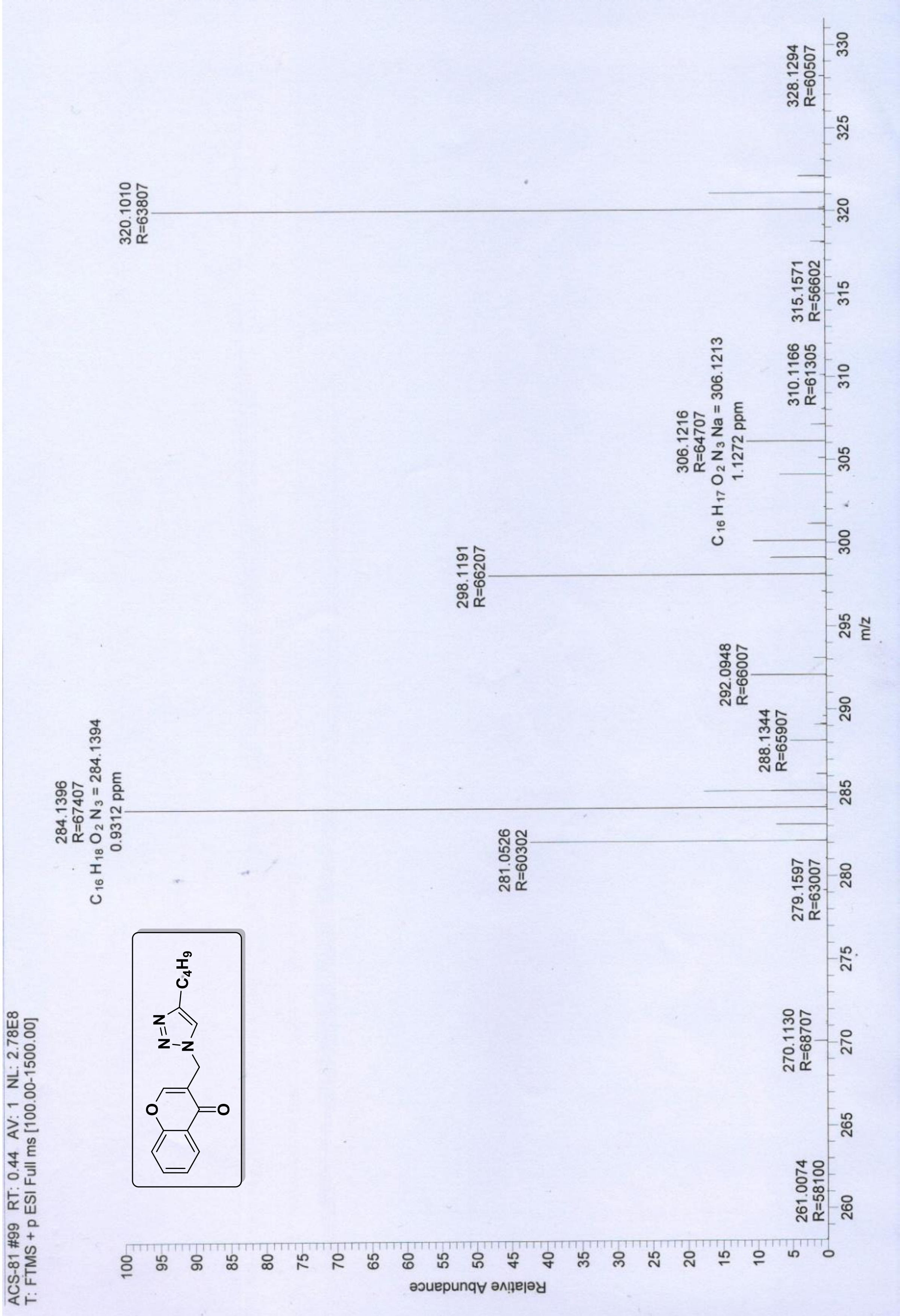

^{1}H NMR (400 MHz, $CDCl_3$) of compound ***6n***:

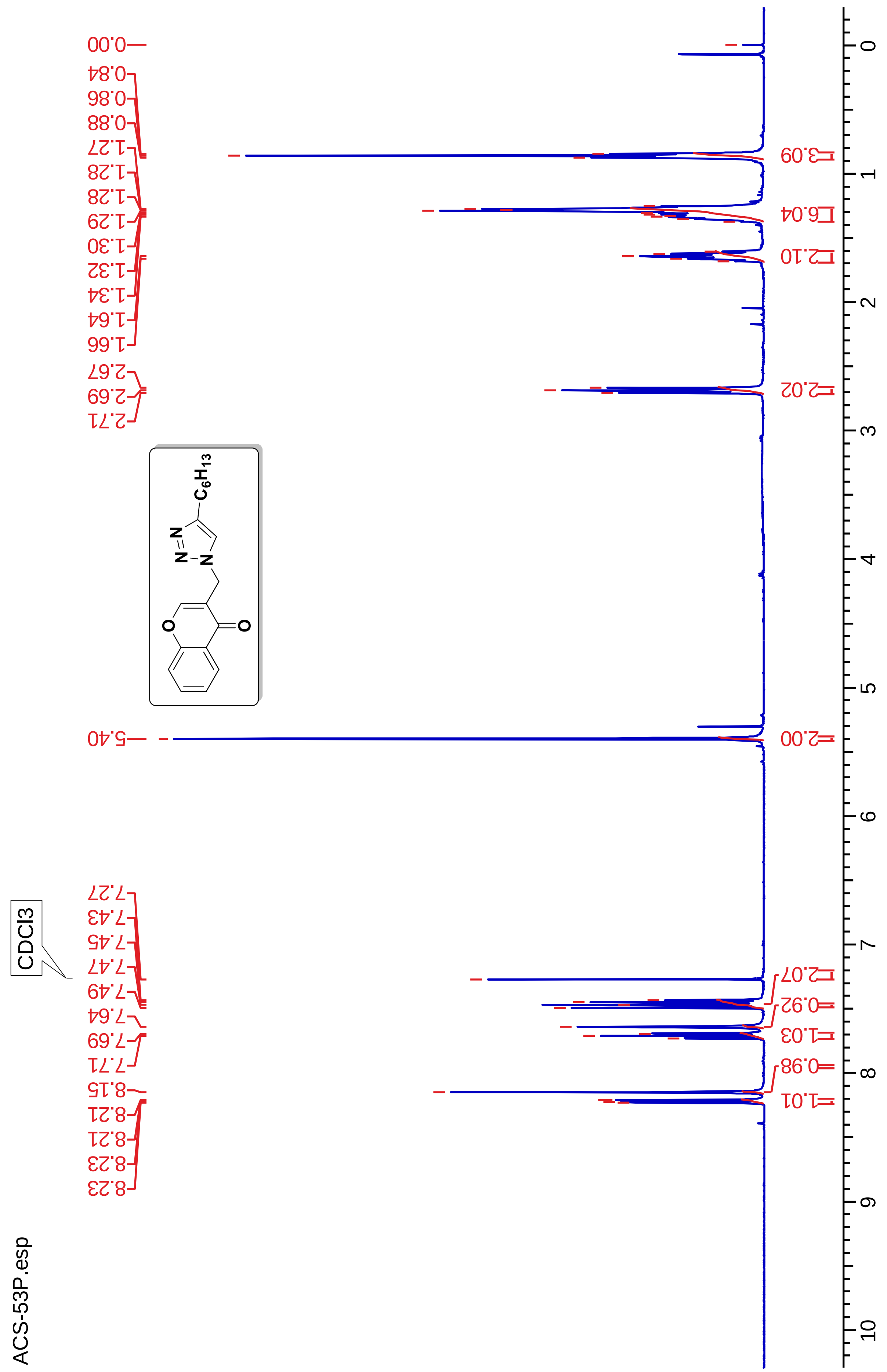

^{13}C NMR (100 MHz, $CDCl_3$) of compound ***6n***:

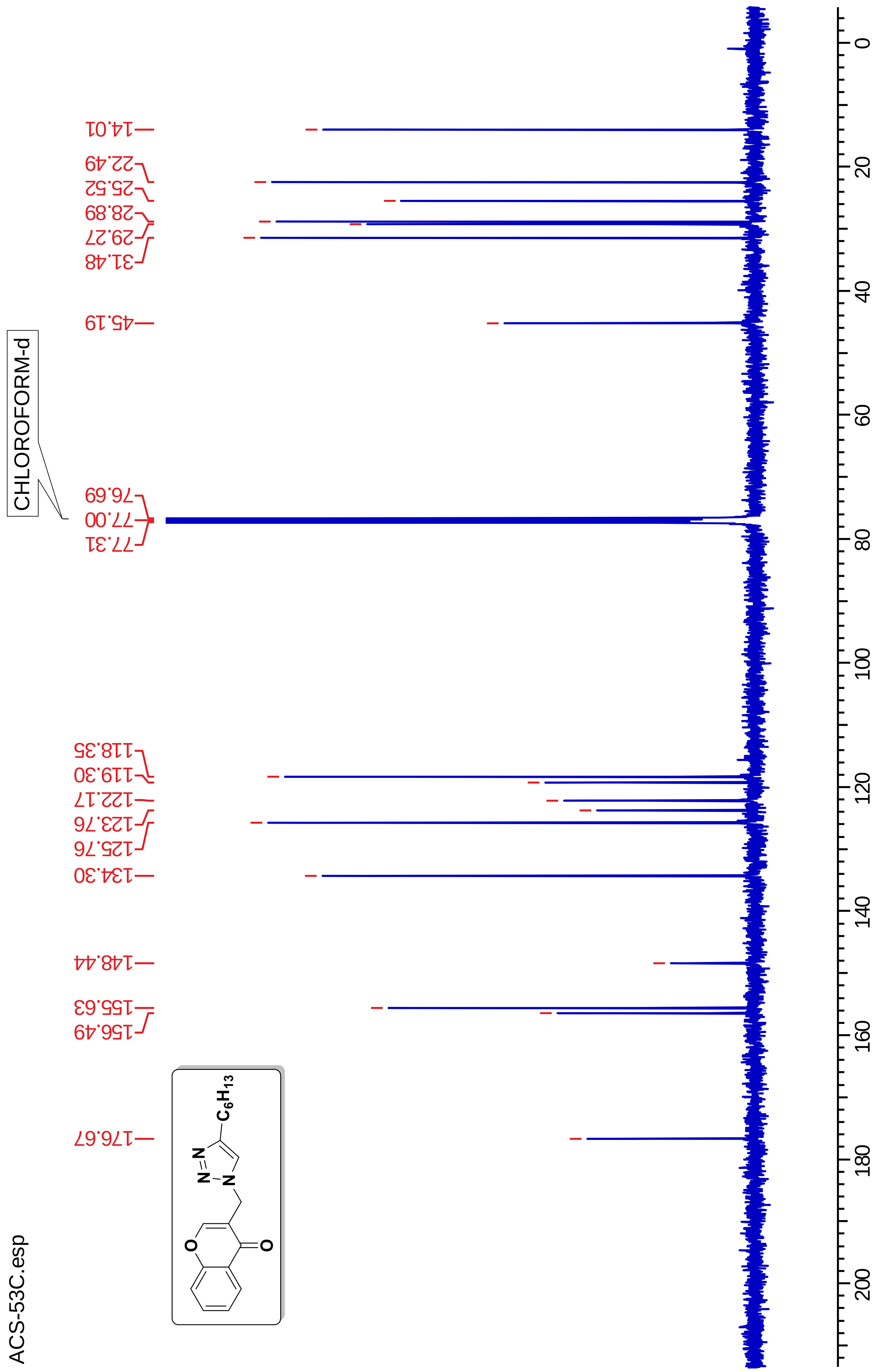

HRMS of compound ***6n***:

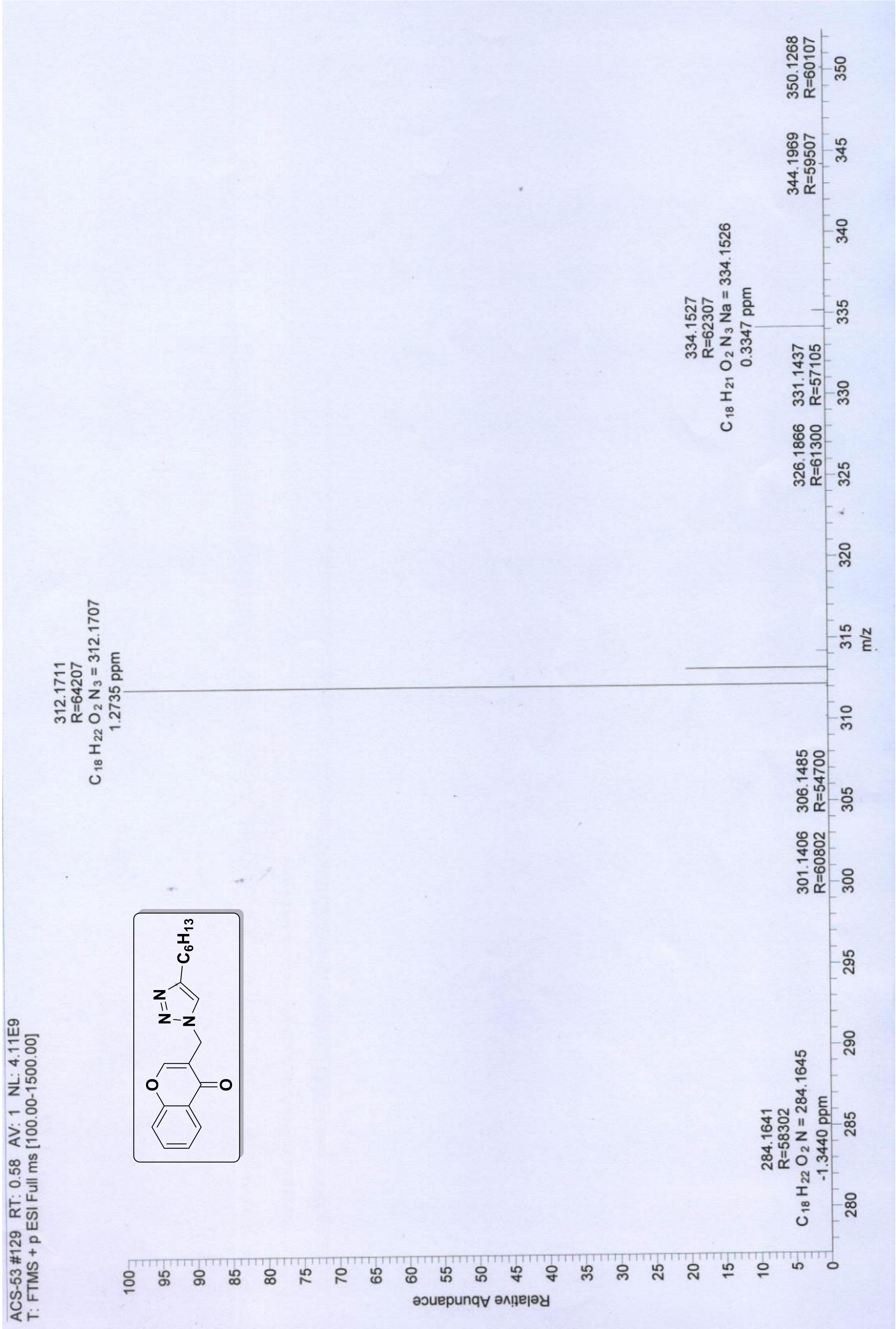

^{1}H NMR (400 MHz, $CDCl_3$) of compound ***6o***:

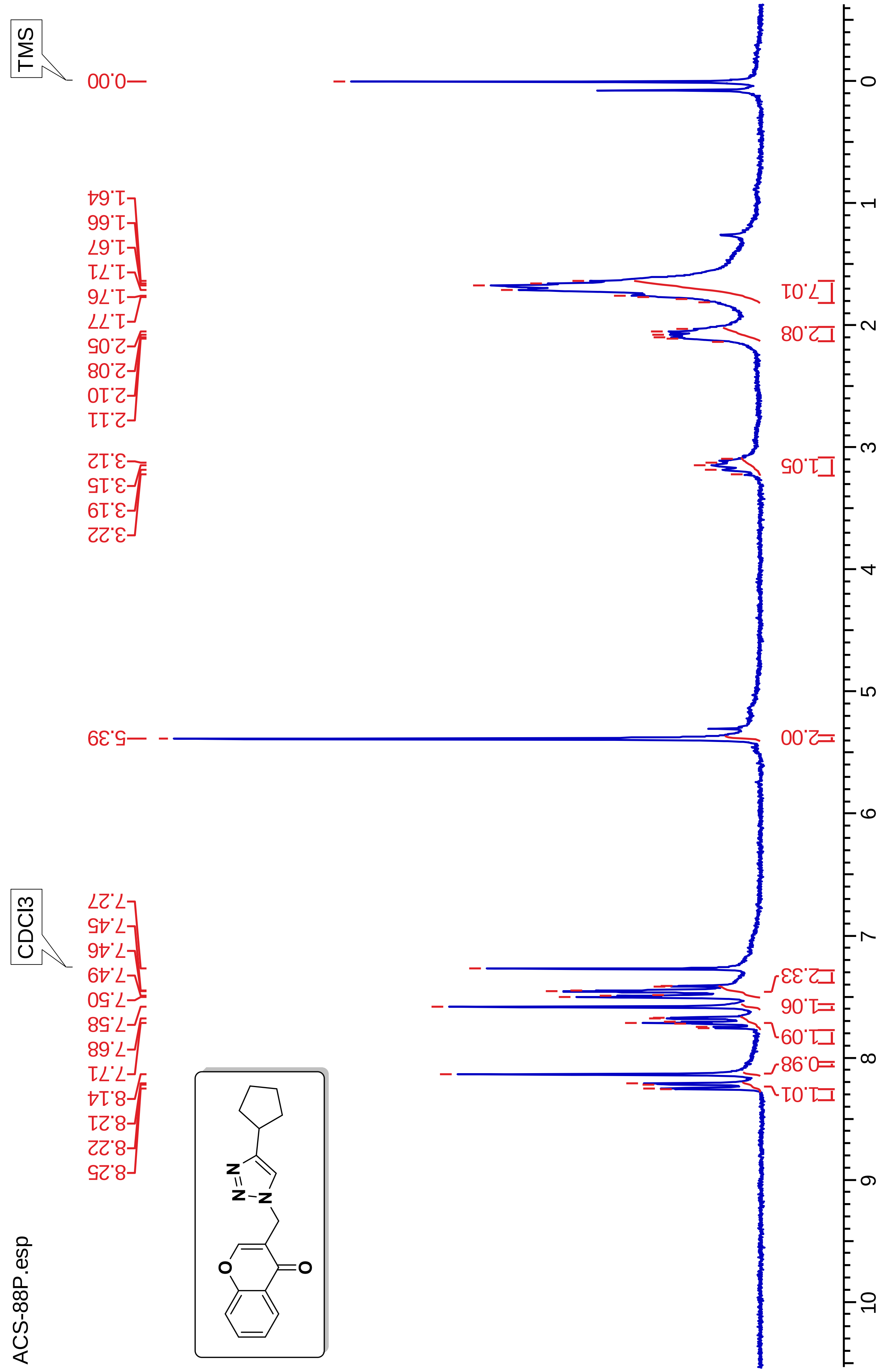

^{13}C NMR (100 MHz, $CDCl_3$) of compound ***6o***:

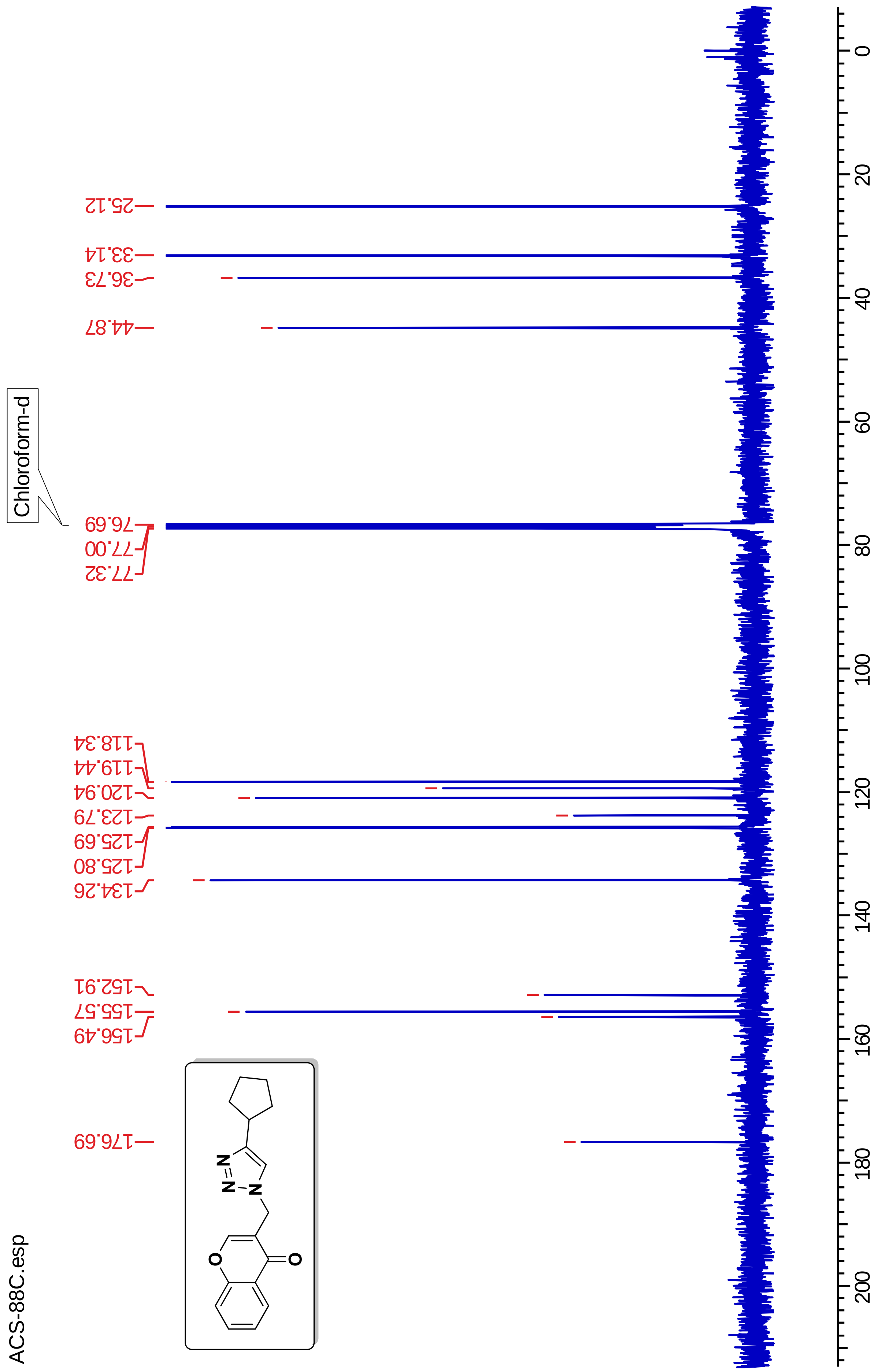

HRMS of compound ***6o***:

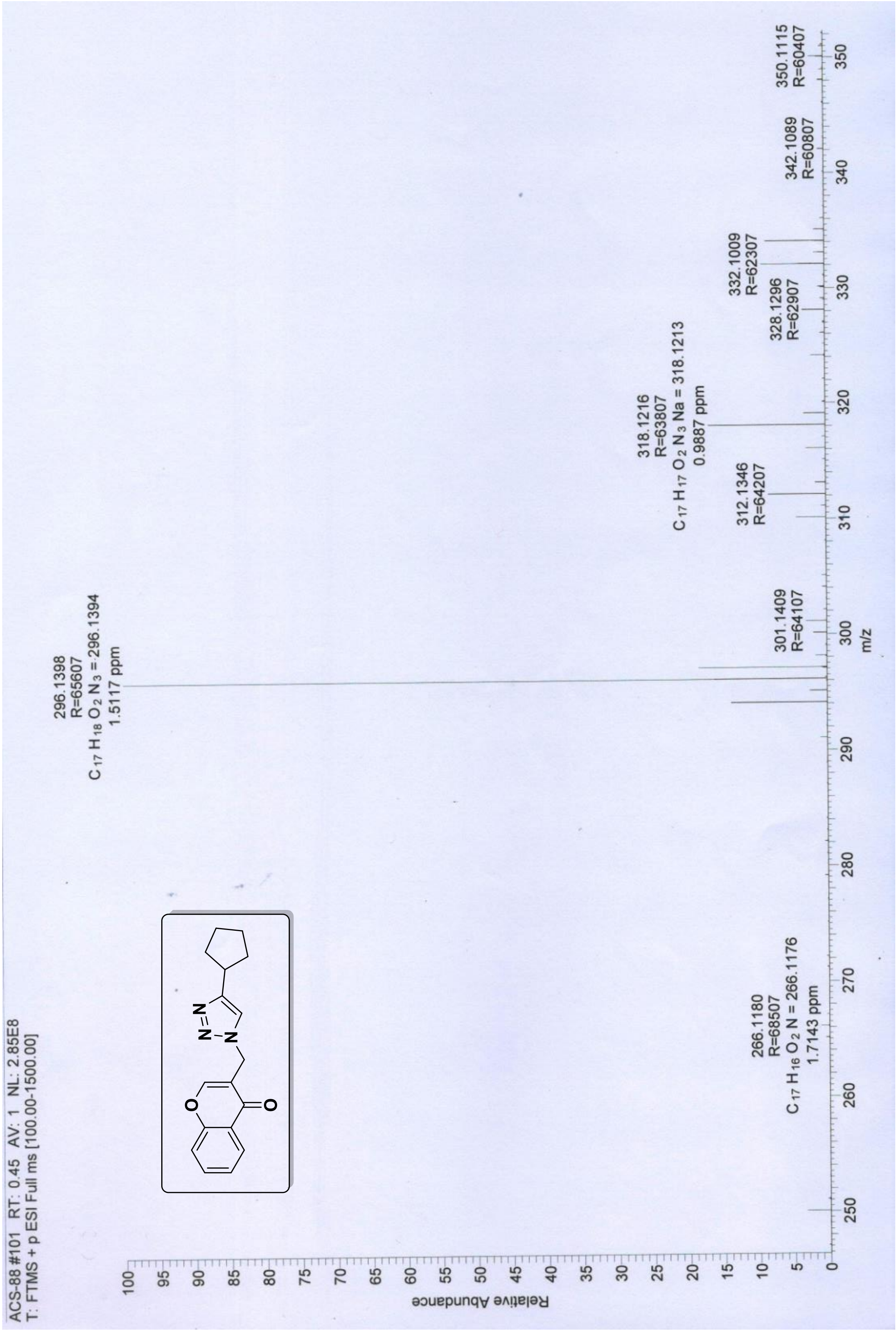

^{1}H NMR (400 MHz, $CDCl_3$) of compound ***6p***:

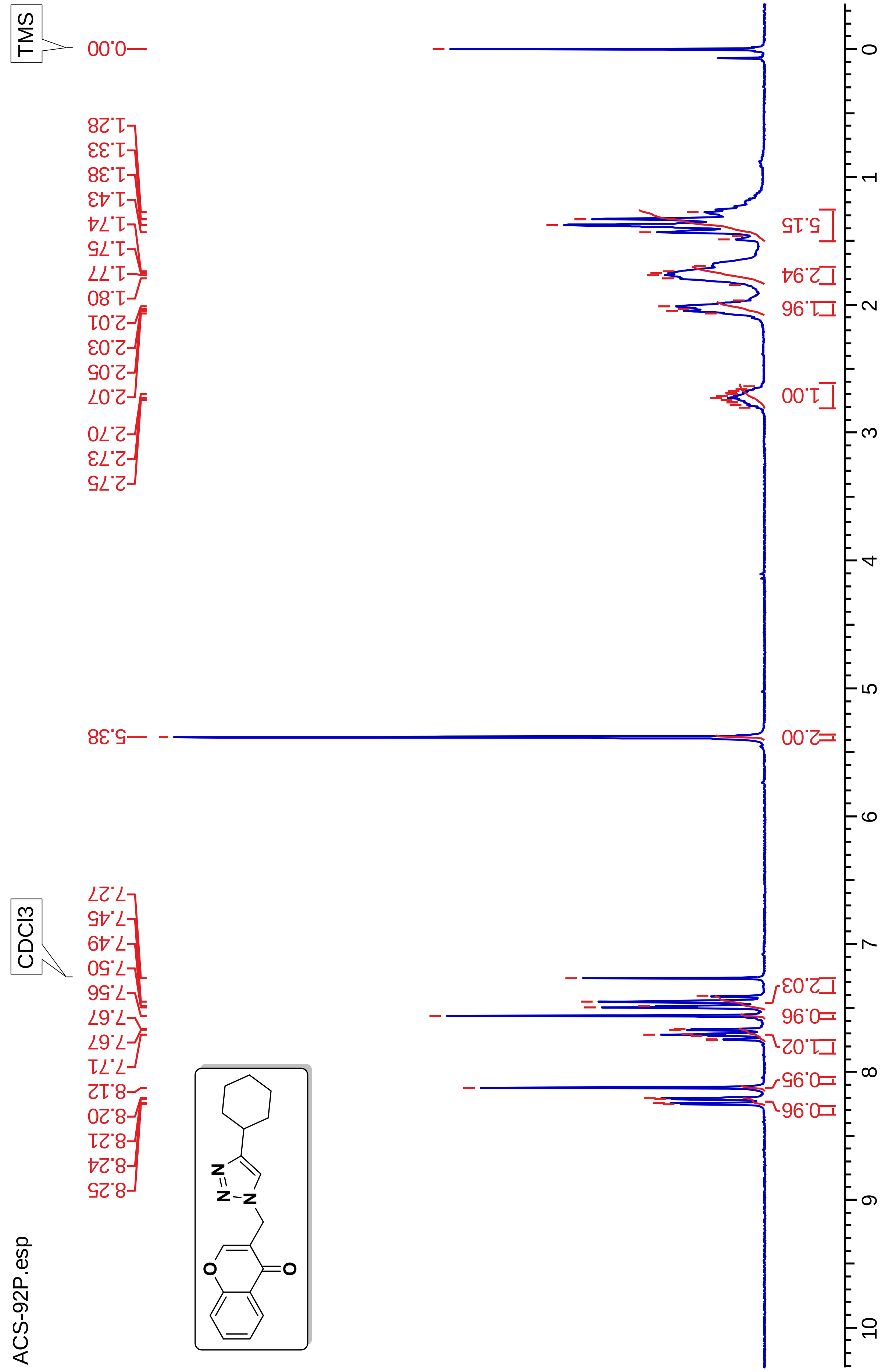

^{13}C NMR (100 MHz, $CDCl_3$) of compound ***6p***:

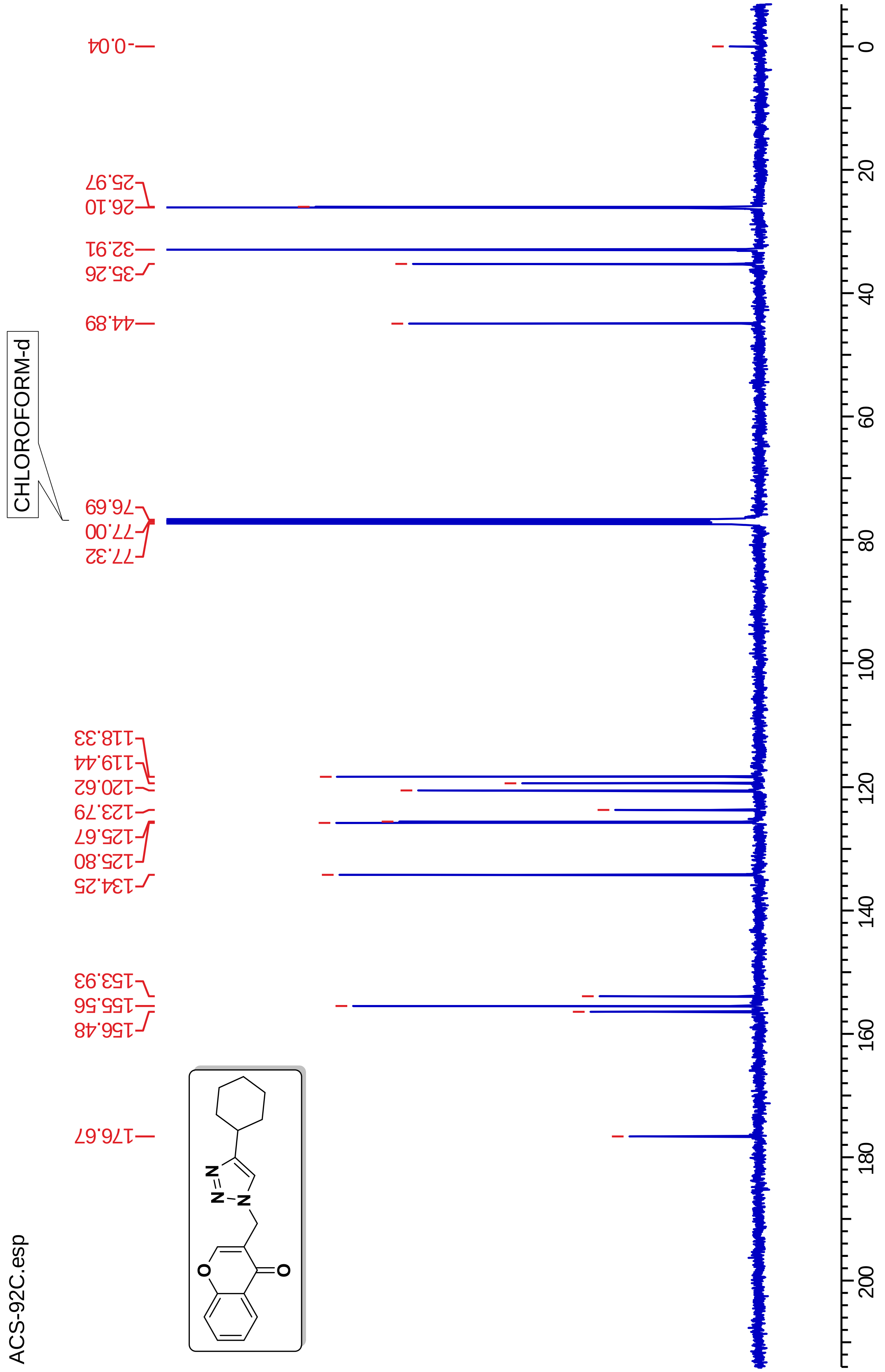

HRMS of compound ***6p***:

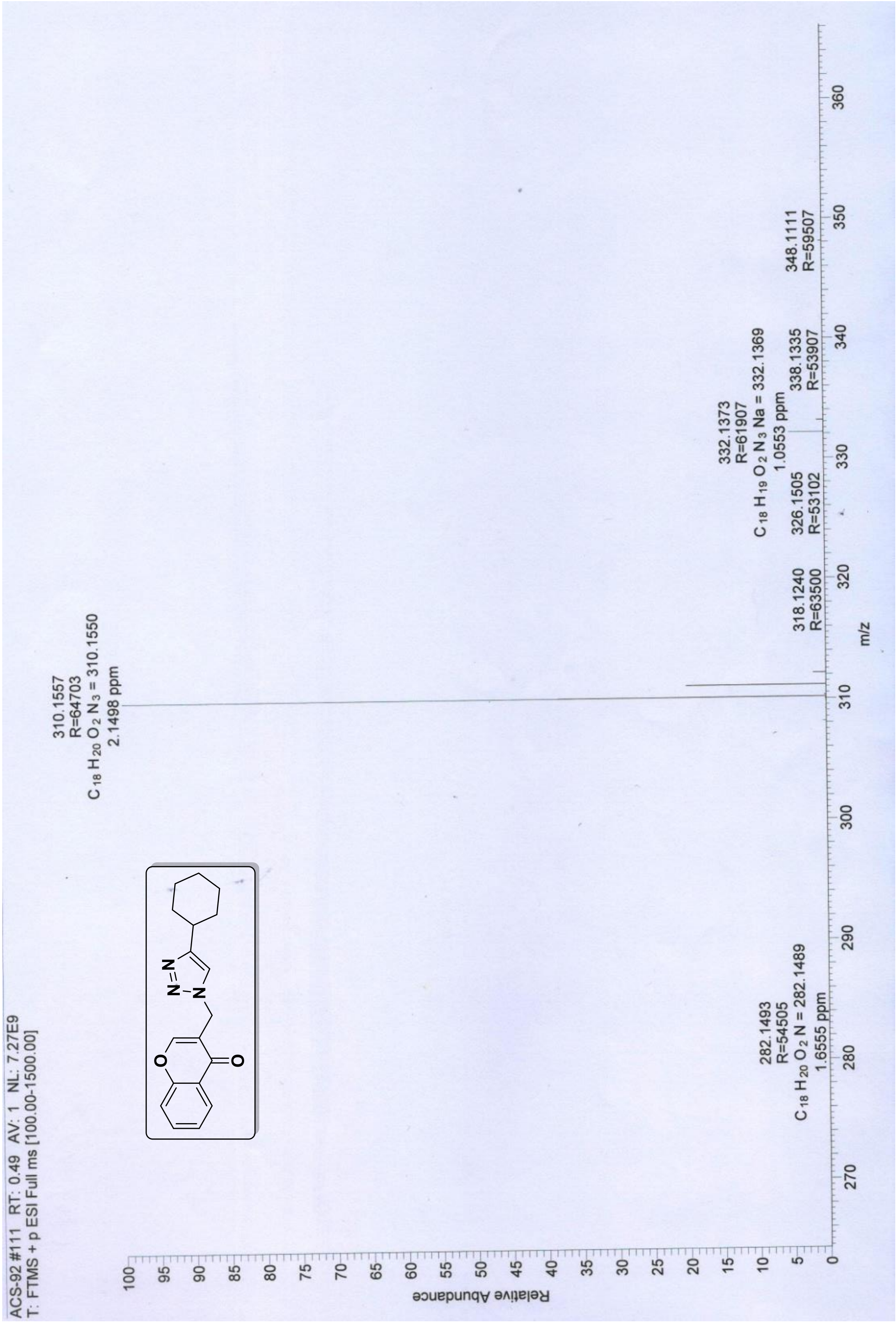

^{1}H NMR (400 MHz, $CDCl_3$) of compound ***6q***:

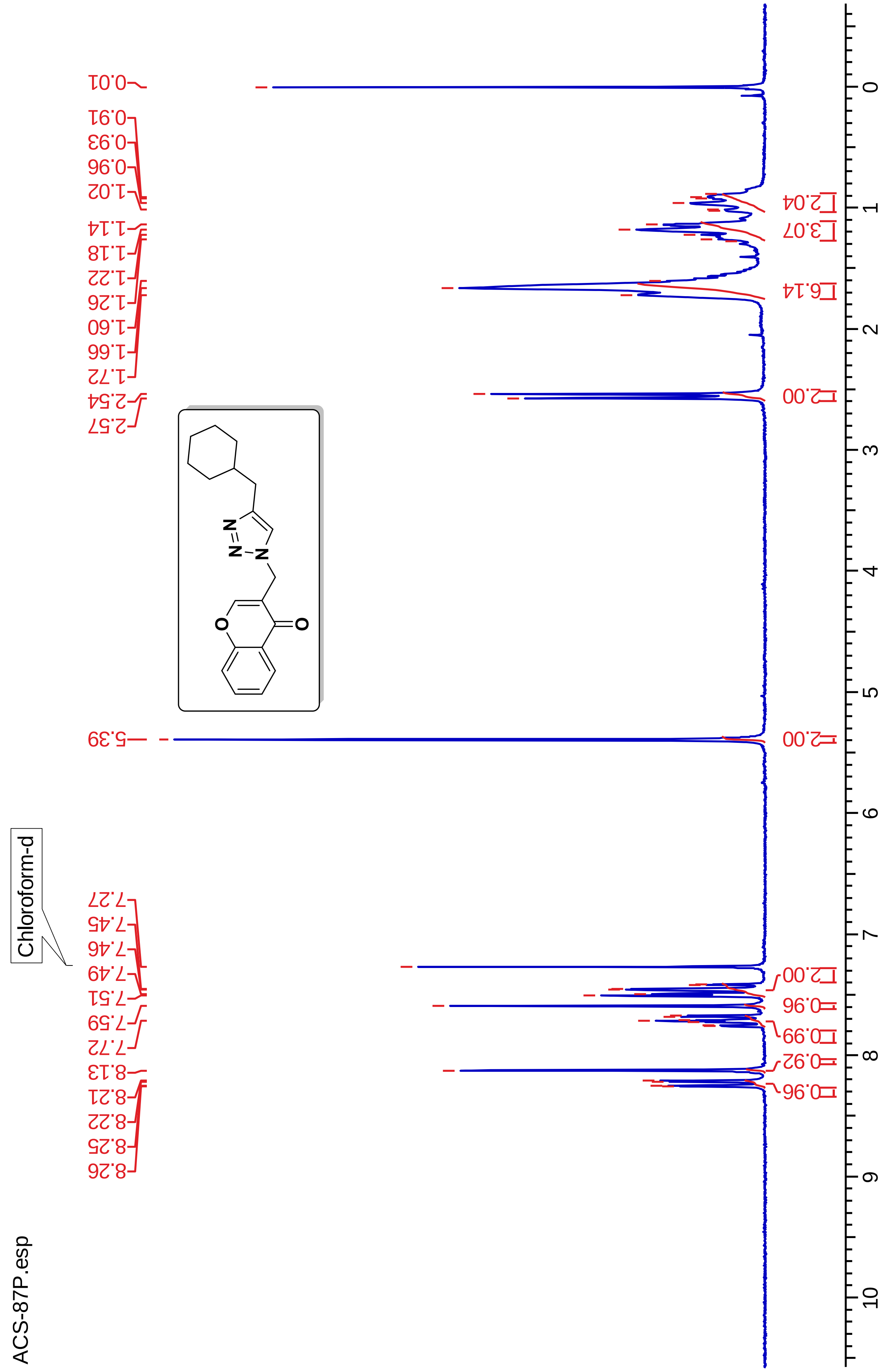

^{13}C NMR (100 MHz, $CDCl_3$) of compound ***6q***:

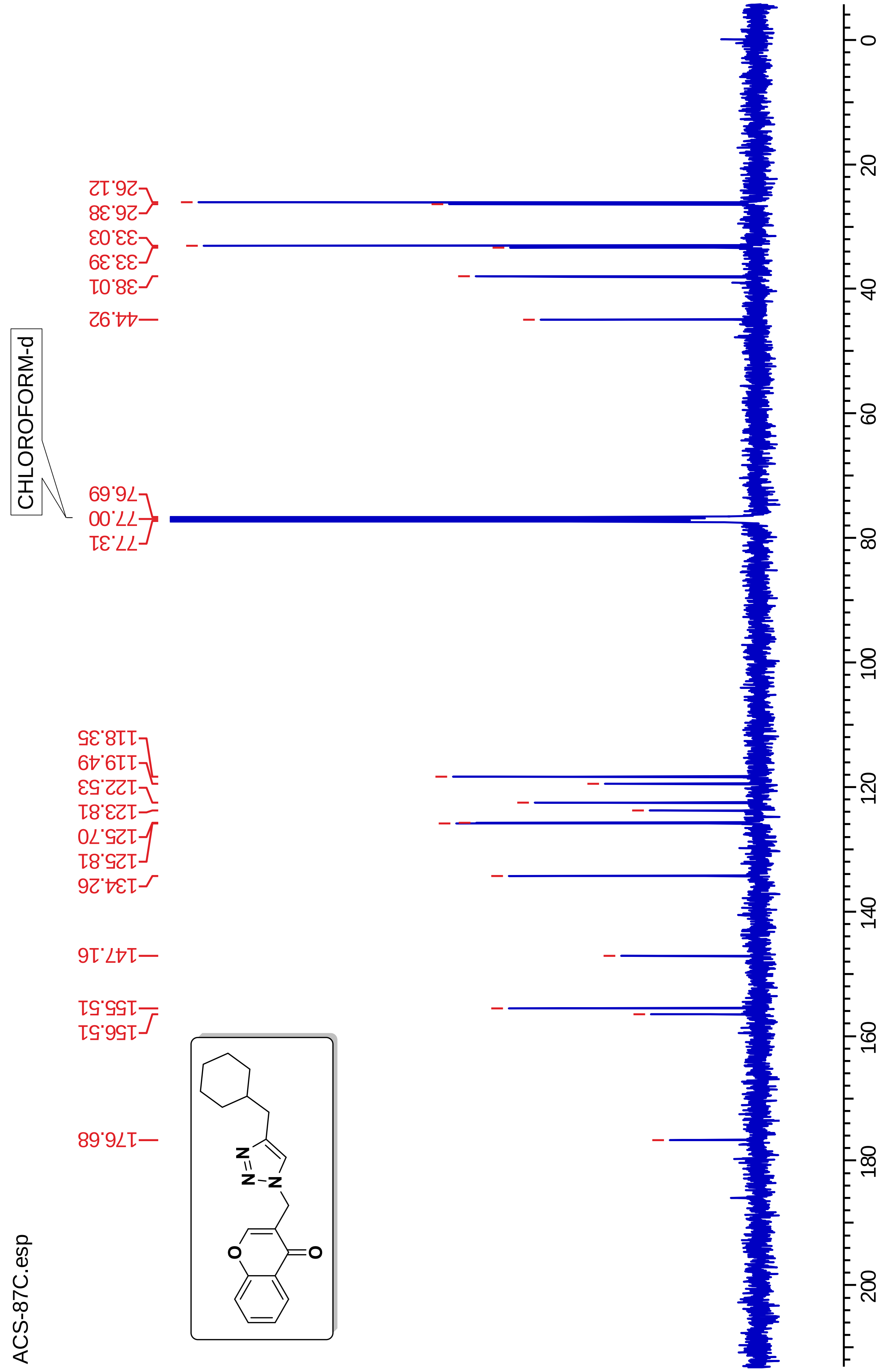

HRMS of compound ***6q***:

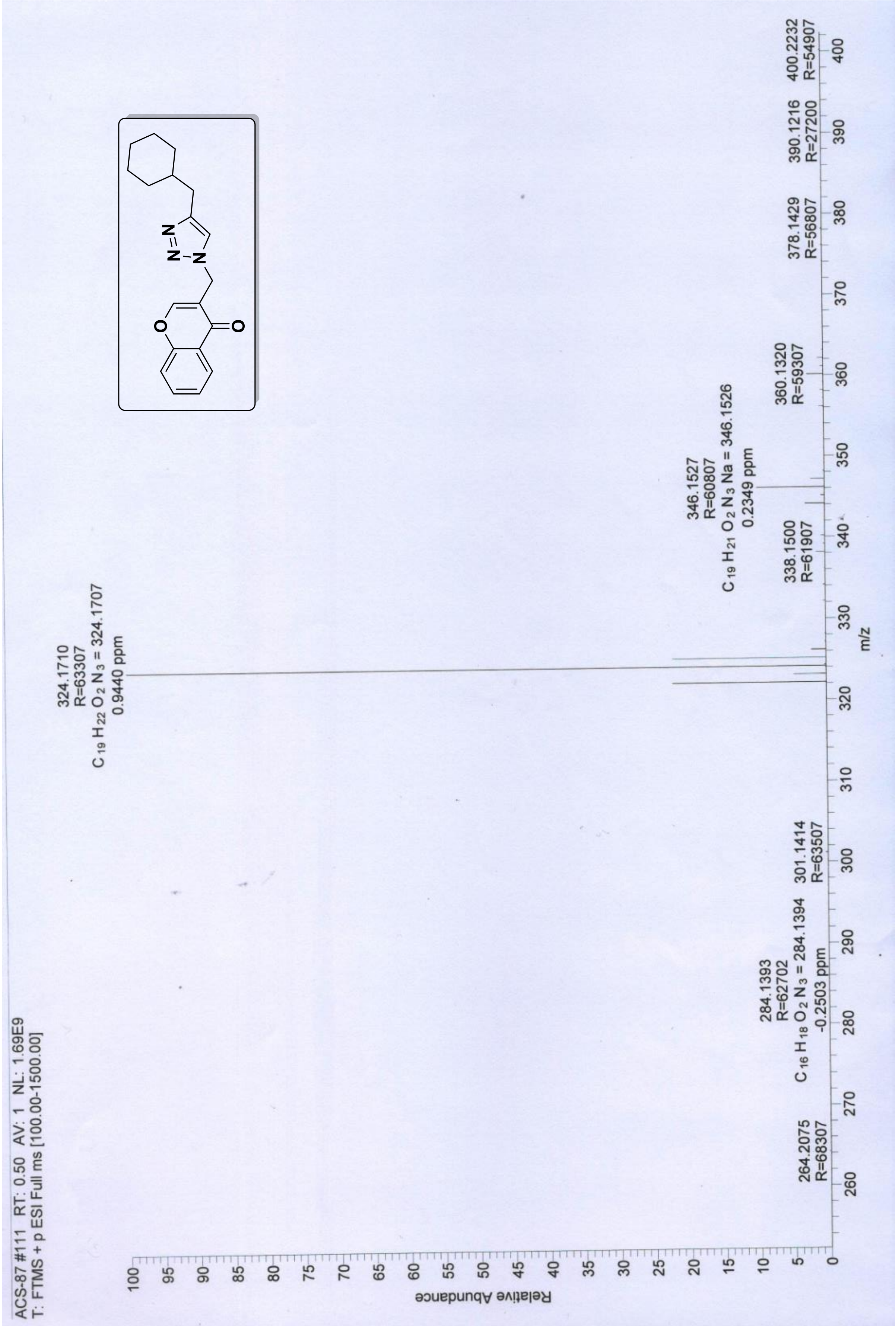

^{1}H NMR (400 MHz, $CDCl_3$) of compound ***6r***:

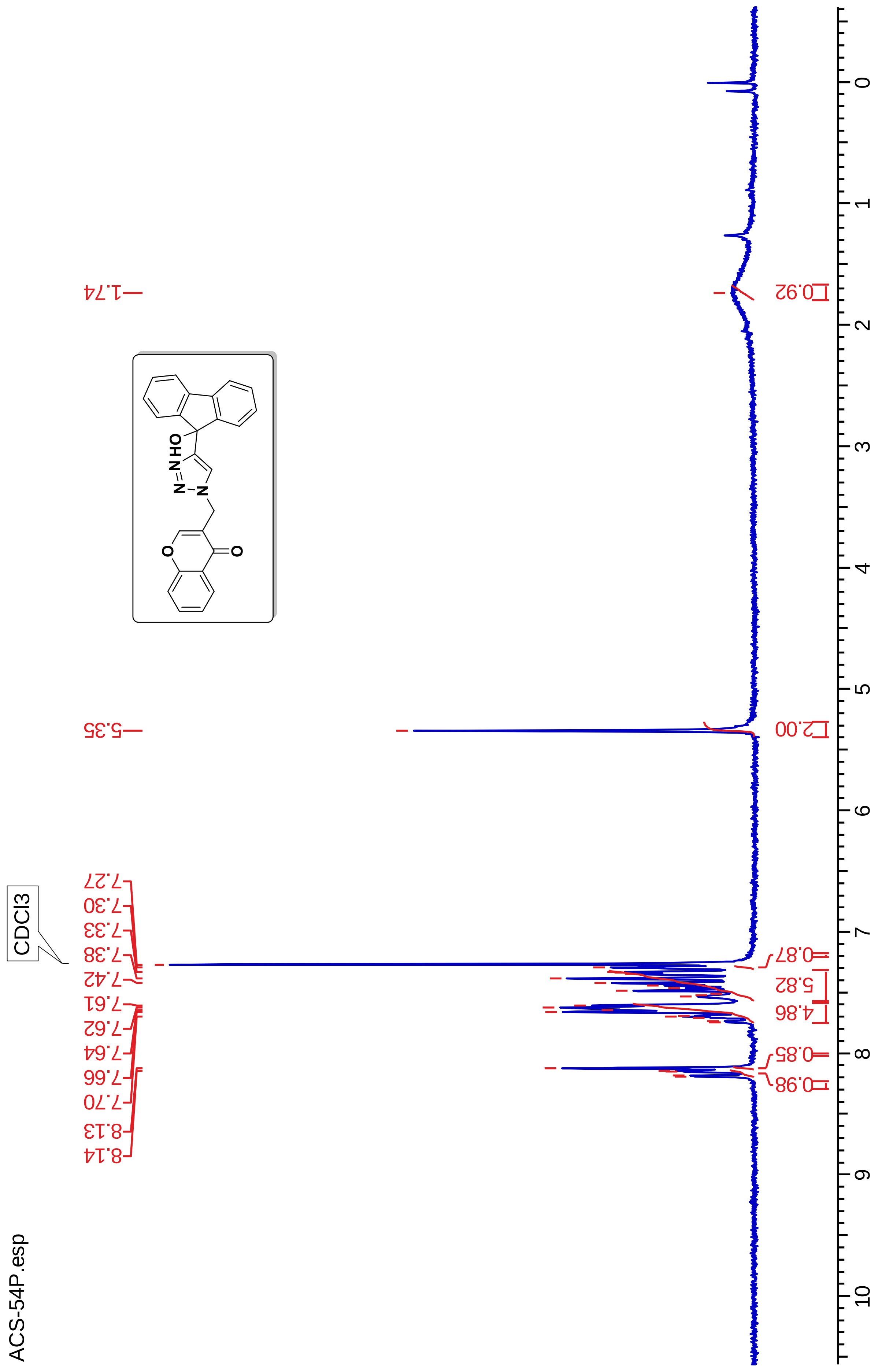

^{13}C NMR (100 MHz, $CDCl_3$) of compound ***6r***:

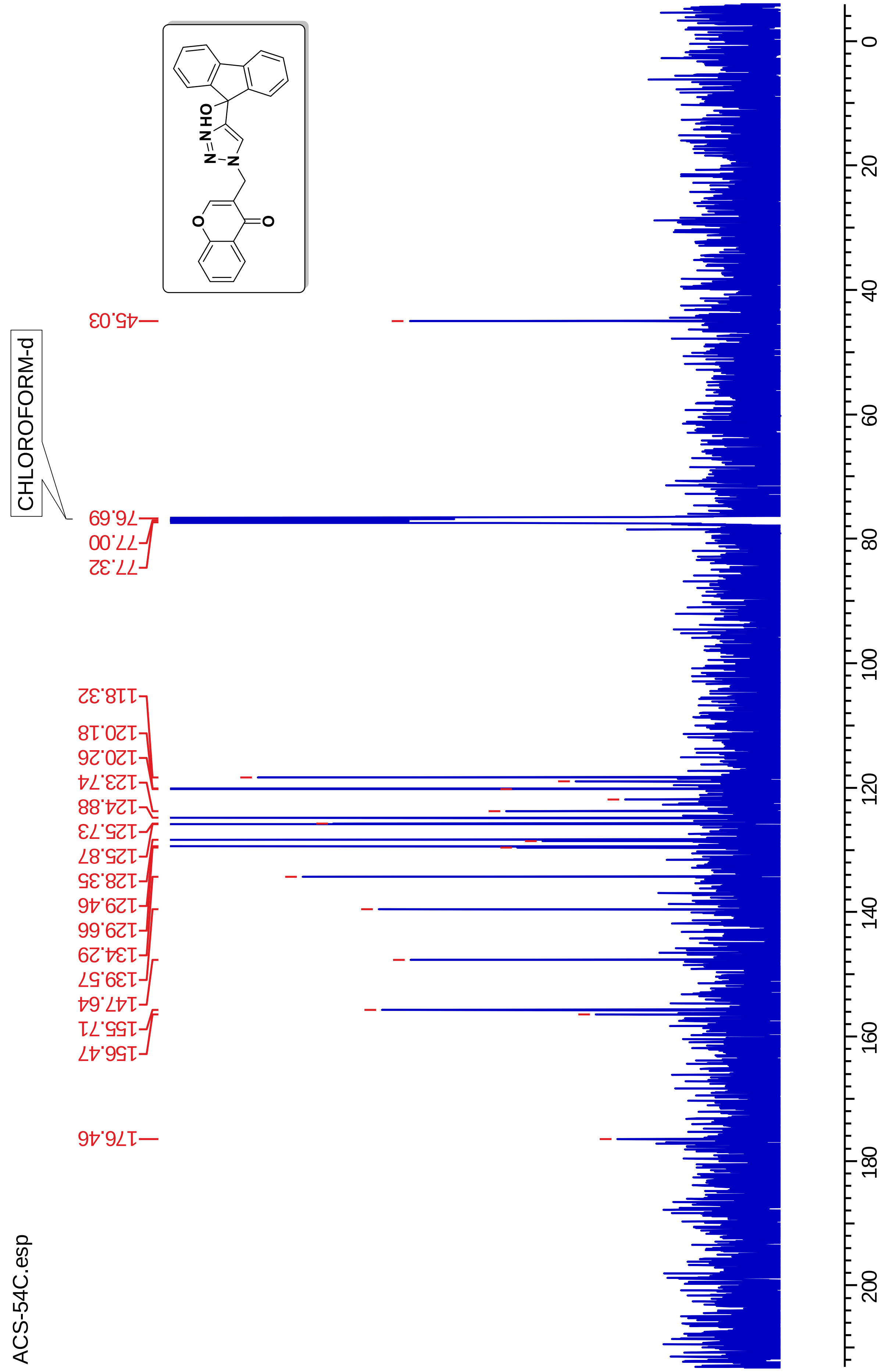

HRMS of compound ***6r***:

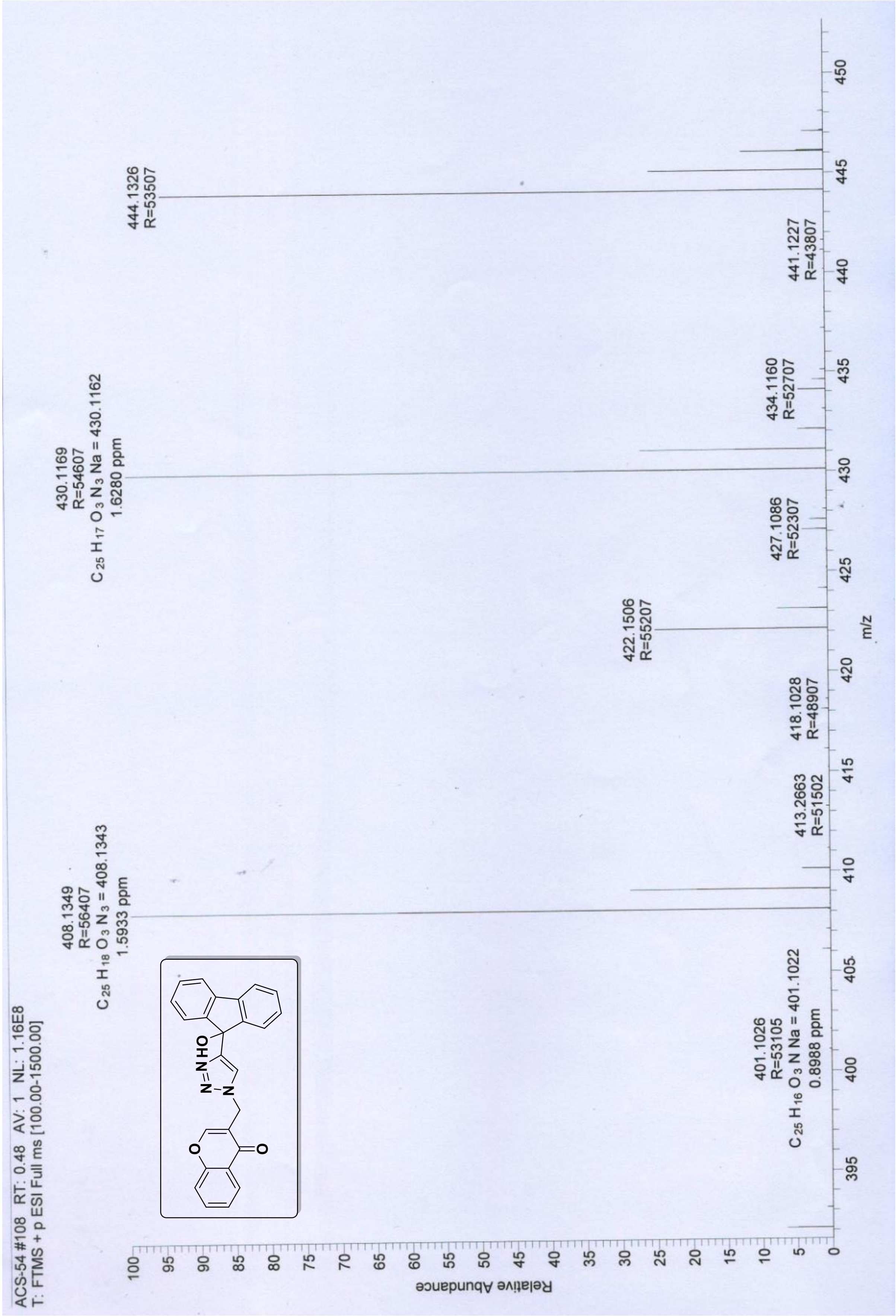

^{1}H NMR (400 MHz, $CDCl_3$) of compound ***6s***:

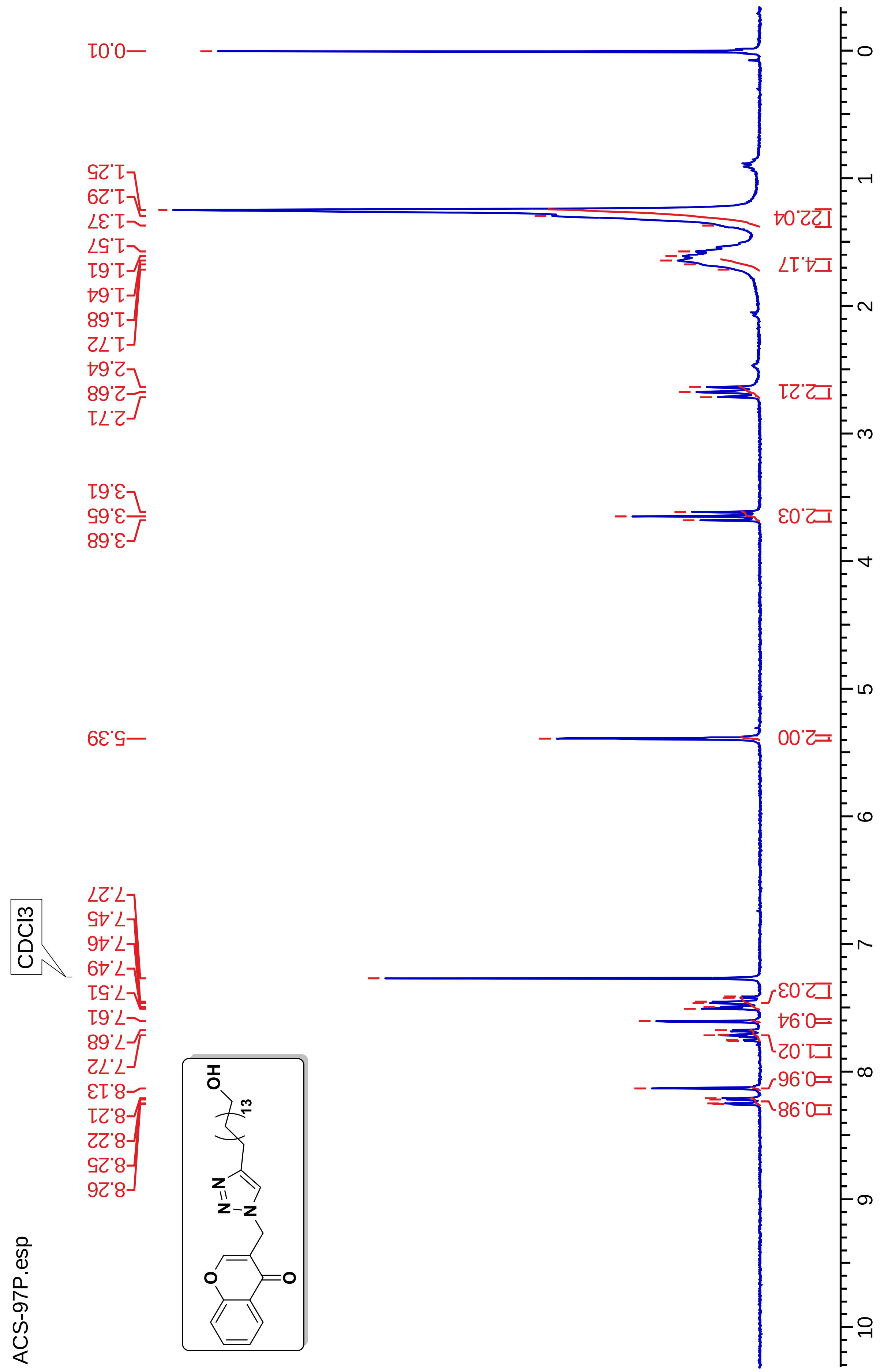

^{13}C NMR (100 MHz, $CDCl_3$) of compound ***6s***:

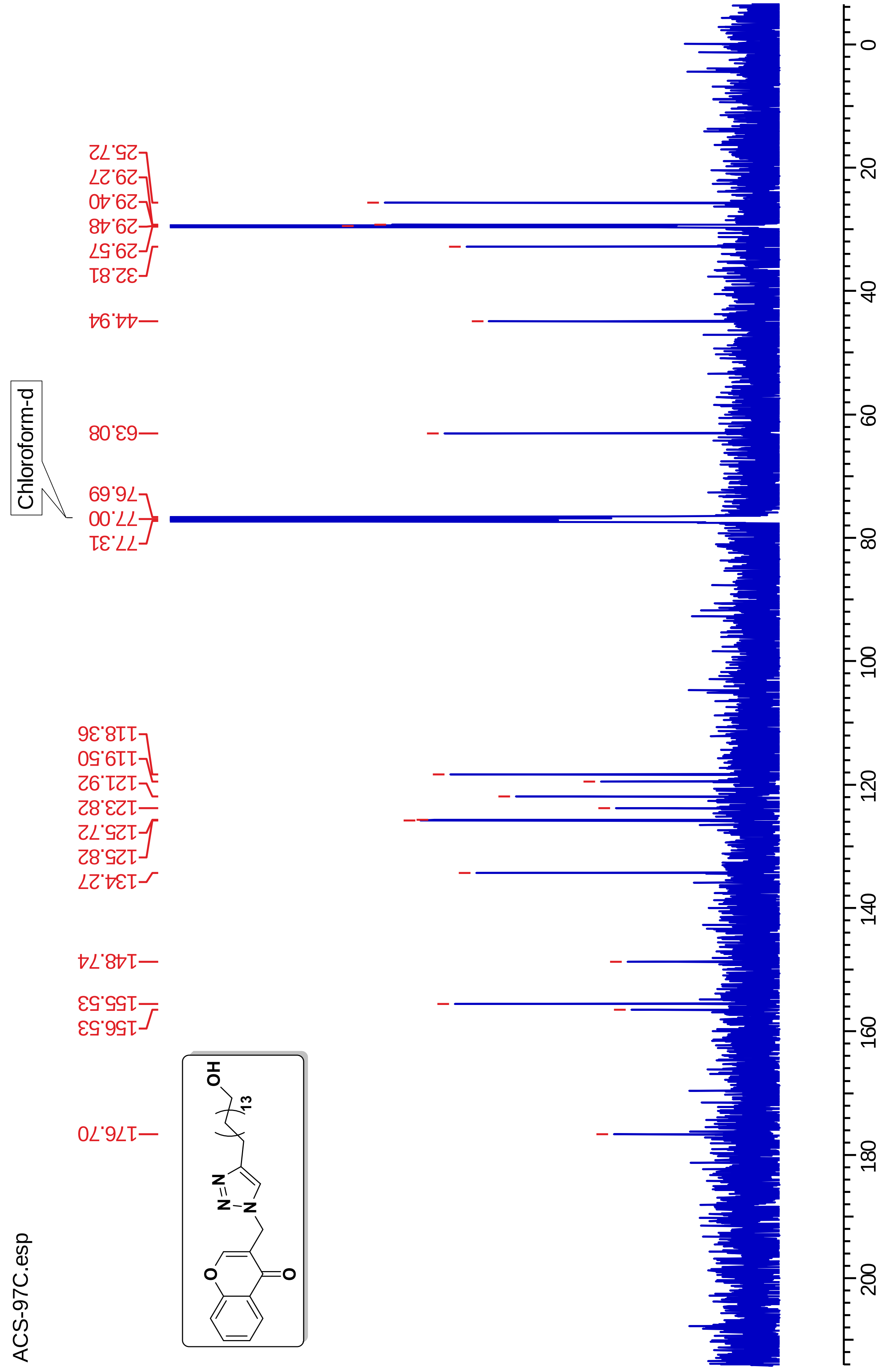

HRMS of compound ***6s***:

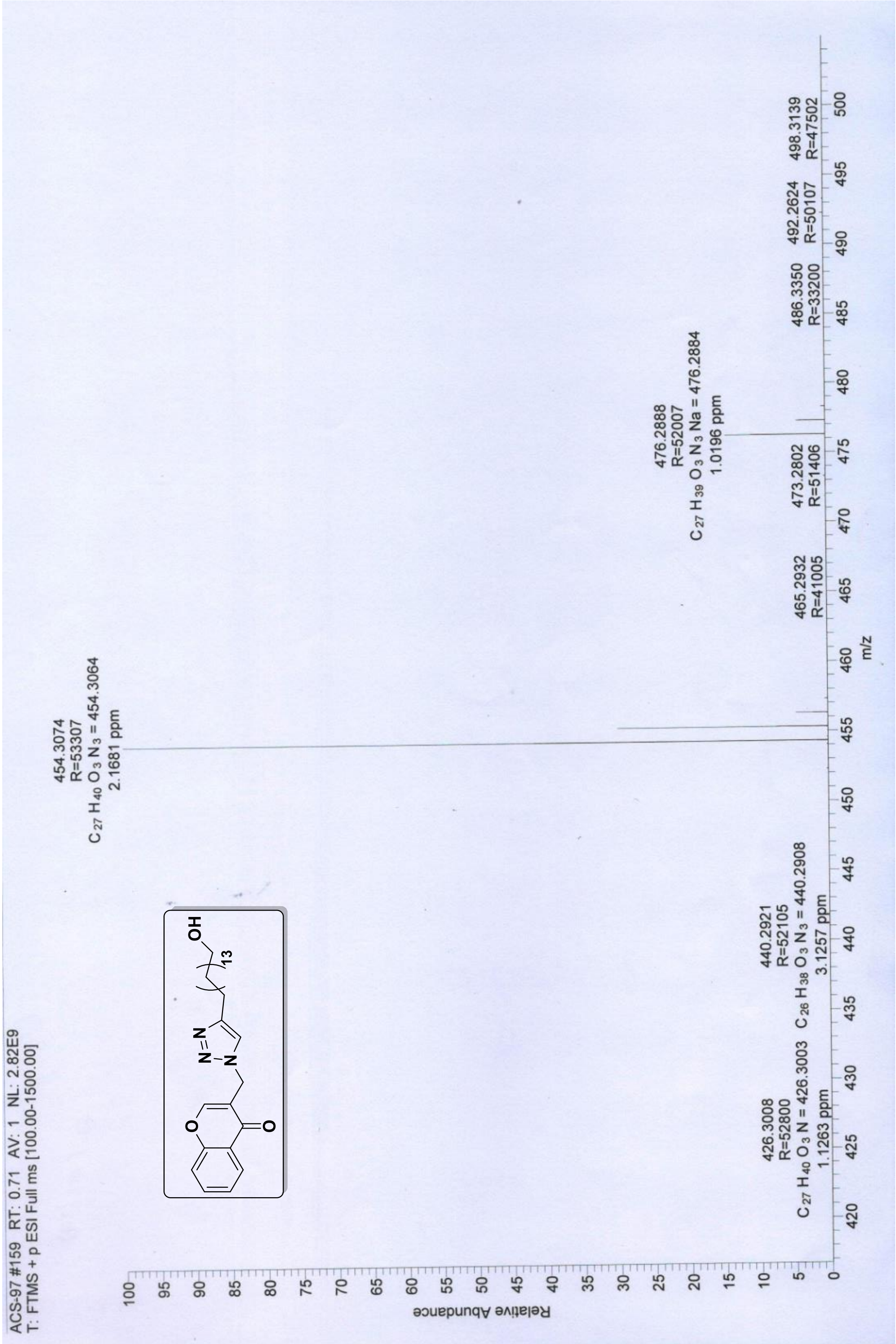

^{1}H NMR (400 MHz, $CDCl_3$) of compound ***6t***:

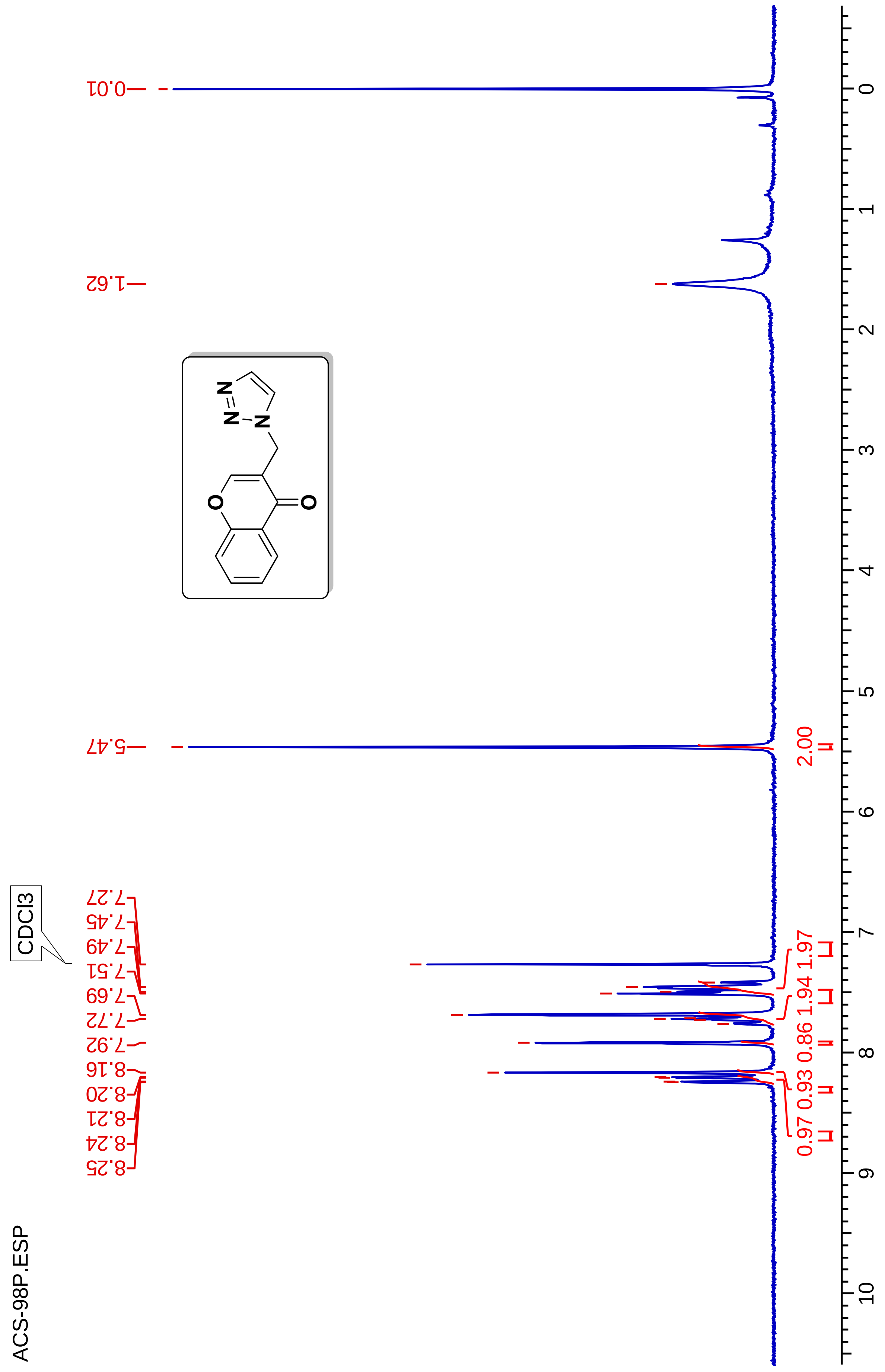

^{13}C NMR (100 MHz, $CDCl_3$) of compound ***6t***:

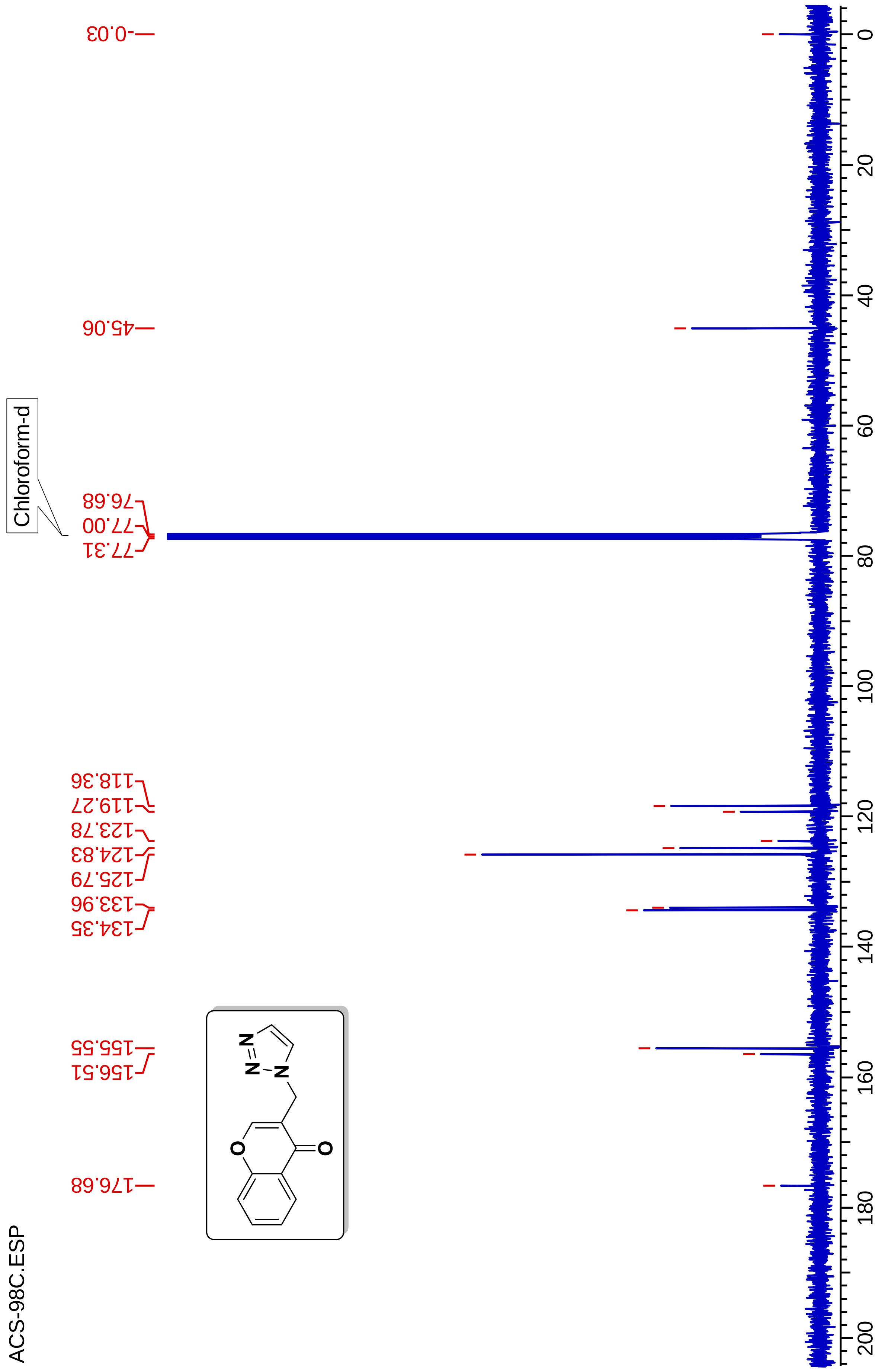

HRMS of compound ***6t***:

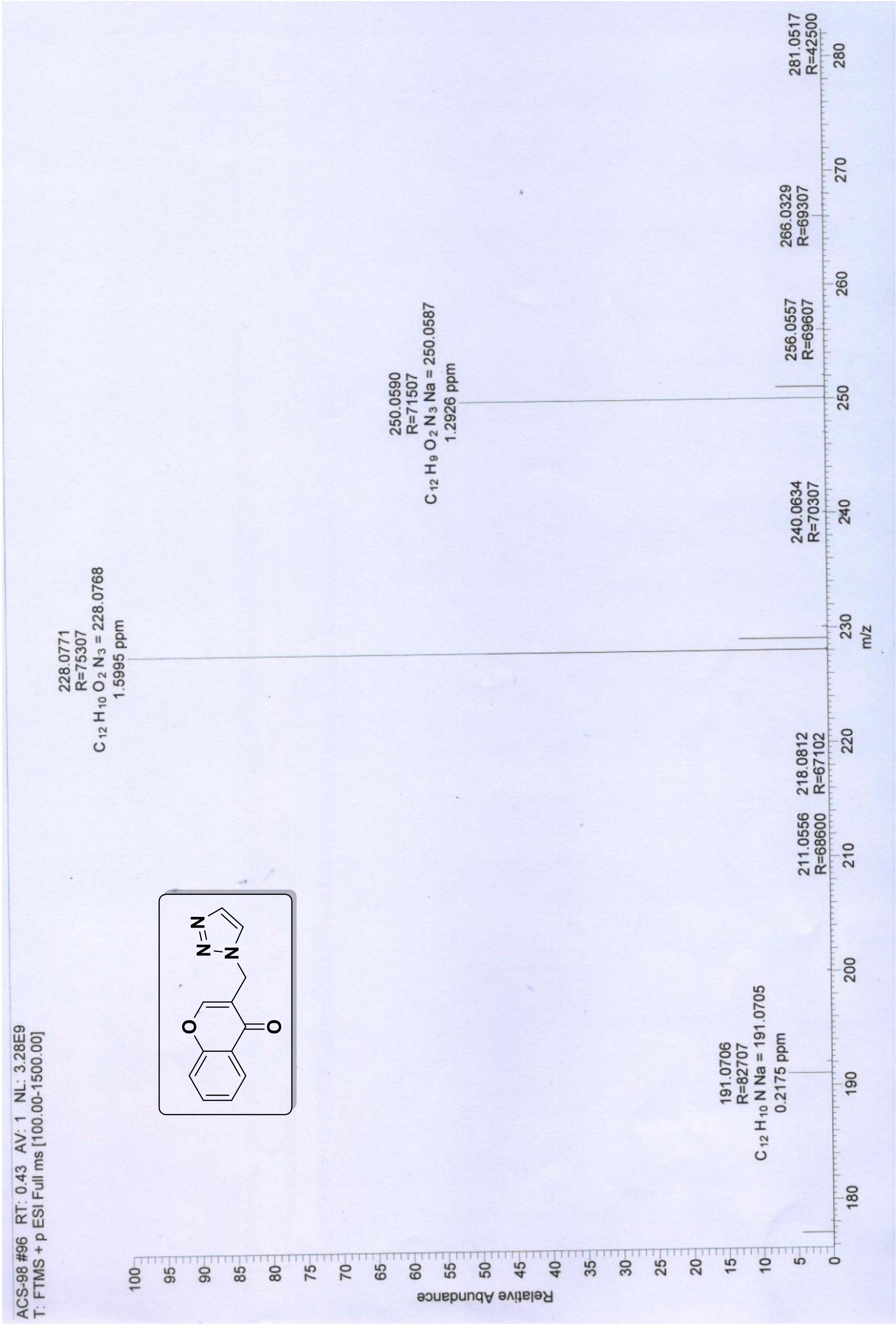

***In-vitro* MTB MABA assay**

Briefly, the inoculum was prepared from fresh LJ medium re-suspended in 7H9-S medium (7H9 broth, 0.1% casitone, 0.5% glycerol, supplemented oleic acid, albumin, dextrose, and catalase [OADC]), adjusted to a McFarland tube No. 1, and diluted 1:20; 100 µl was used as inoculum. Each drug stock solution was thawed and diluted in 7H9-S at four-fold the final highest concentration tested. Serial two-fold dilutions of each drug were prepared directly in a sterile 96-well microtiter plate using 100 µl 7H9-S. A growth control containing no antibiotic and a sterile control were also prepared on each plate. Sterile water was added to all perimetre wells to avoid evaporation during the incubation. The plate was covered, sealed in plastic bags and incubated at 37°C in normal atmosphere. After 7 days incubation, 30 ml of alamar blue solution was added to each well, and the plate was re-incubated overnight. A change in colour from blue (oxidised state) to pink (reduced) indicated the growth of bacteria, and the MIC was defined as the lowest concentration of drug that prevented this change in colour.

***In-vitro* cytotoxicity screening**

Some compounds were further examined for toxicity in a RAW 264.7 cell line at the concentration of 50 µM. After 72 h of exposure, viability was assessed on the basis of cellular conversion of MTT into a formazan product using the Promega Cell Titer 96 non-radioactive cell proliferation assay.

	MIC in µM
Rifampicin	0.24
Ethambutol	7.64

Correlation of bioactivity with respect to GLIDE Score Binding Energy (kcal/mol) of 1ZID (Enoyl-acyl carrier protein)

Figure:

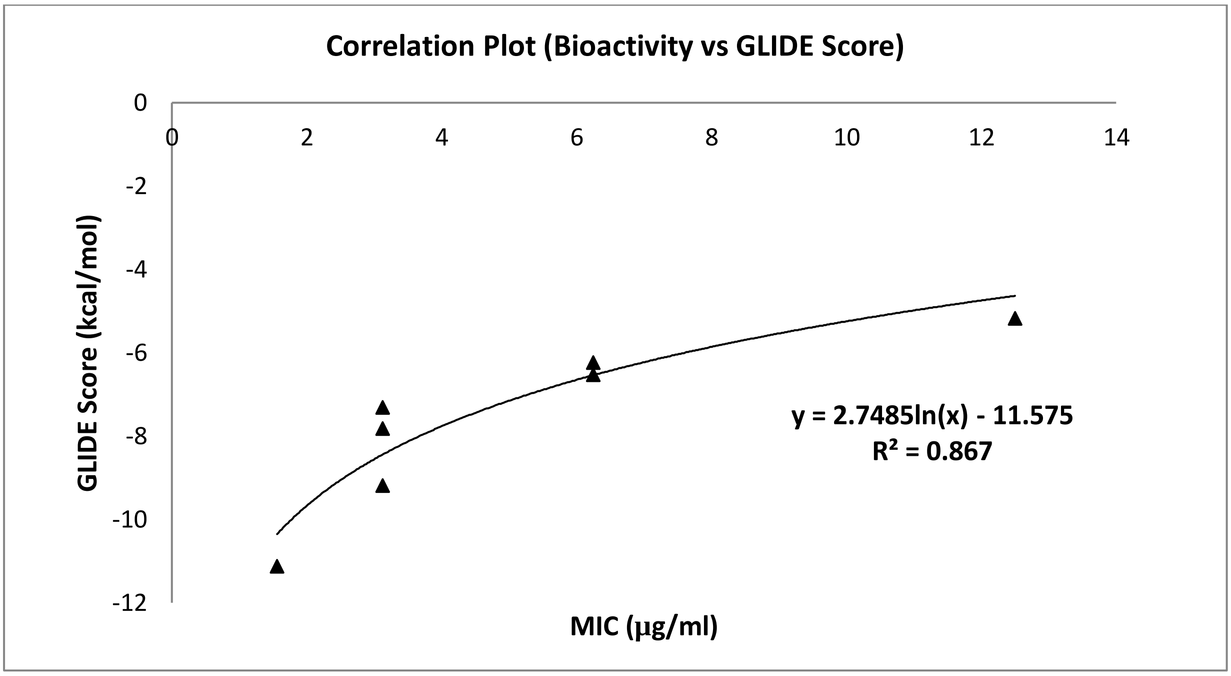

Table: In vitro anti-tubercular activity MIC (µg/ml) and GLIDE Score (kcal/mol)

Compound	MIC (µg/ml)	GLIDE Score (BE)
**6s**	1.56	-11.123
**6h**	3.125	-9.189
**6f**	3.125	-7.826
**6o**	3.125	-7.316
**6m**	6.25	-6.525
**6g**	6.25	-6.238
**6p**	12.5	-5.176
